# MicroRNA Profiling During Mulberry (*Morus atropurpurea* Roxb) Fruit Development and Regulatory Pathway of miR477 for Anthocyanin Accumulation

**DOI:** 10.3389/fpls.2021.687364

**Published:** 2021-09-08

**Authors:** Xiaonan Dong, Chaorui Liu, Yuqi Wang, Qing Dong, Yingping Gai, Xianling Ji

**Affiliations:** ^1^College of Forestry, Shandong Agricultural University, Tai’an, China; ^2^State Key Laboratory of Crop Biology, Shandong Agricultural University, Tai’an, China

**Keywords:** *Morus atropurpurea*, miRNA profiles, fruit ripening, miR477, anthocyanin

## Abstract

To understand the mechanism of small non-coding RNAs (miRNA)-mediated development and ripening of mulberry fruits, three small RNA libraries from mulberry fruits at different development stages were constructed, and 159 conserved miRNAs as well as 86 novel miRNAs were successfully identified. Among the miRNAs identified, there were 90 miRNAs which showed differential expression patterns at different stages of fruit development and ripening. The target genes of these differential expressed (DE) miRNAs were involved in growth and development, transcription and regulation of transcription, metabolic processes, and etc. Interestingly, it was found that the expression level of mul-miR477 was increased with fruit ripening, and it can target the antisense lncRNA (*Mul-ABCB19AS*) of the ATP binding cassette (ABC) transporter B 19 gene (*Mul-ABCB19*). Our results showed that mul-miR477 can repress the expression of *Mul-ABCB19AS* and increase the expression of *Mul-ABCB19*, and it acted as a positive regulator participating anthocyanin accumulation through the regulatory network of mul-miR477—*Mul-ABCB19AS*—*Mul-ABCB19*.

## Introduction

miRNAs are small non-coding RNAs, approximately 18–24 nucleotides in length, which modulate gene expression by cleaving or inhibiting the translation of their target genes (Yu et al., 2017). The research on the function and mechanism of miRNA has made great progress, and it has been demonstrated that miRNAs are involved in various biological processes of plants such as development, hormone response and signaling, stress responses and so on (Rogers and Chen, 2013; Ge et al., 2017; Betti et al., 2020). There are many evidences showed that miRNAs play significant roles in regulating fruit development and ripening (Zeng et al., 2015; Guo et al., 2018; Deepika et al., 2020). In the tomato fruit, it was showed that miR156 control the expression of the auxin response factor, *COLORLESS NON-RIPENING* encoding a SQUAMOSA promoter-binding protein (SBP), and affect tomato ripening (Moxon et al., 2008). It was also found that miR156b could target the *SBP* genes and affect the expression of genes associated with meristem maintenance. Moreover, overexpression of the miR156b precursor of Arabidopsis in tomato resulted in abnormal fruit morphology (Ferreirae Silva et al., 2014). In addition, it was showed that miR164 and miR169 can inhibit the expression of NAC and NF-YA transcription factors and participated in fruit ripening, respectively (Li et al., 2017), and miR172 regulates fruit ripening through targeting the *APETALA2a* (*AP2a*) gene which negatively regulates ethylene biosynthesis and signaling (Karlova et al., 2011). Apart from these conserved miRNAs, some non-conserved miRNAs, such as miR1917, miR4376, miR858, etc., were also found to be associated with tomato fruit ripening (Christopher et al., 2020). With the development of sRNA sequencing technology, many miRNAs associated with fruit development have been identified in different plant species, such as strawberry (*Fragaria ananassa*) (Xu et al., 2013), sweet orange (*Citrus sinensis*) (Liu et al., 2014), peach (*Pyrus bretschneideri*) (Wu et al., 2014), banana (*Musa acuminata*) (Bi et al., 2015), apple (*Malus* × *domestica*) (Qu et al., 2016), etc. Despite the fact that many miRNAs involved in fruit development and ripening have been identified, only a few miRNA targets have been experimentally proved for their biological functions and the roles of most miRNAs in fruit ripening still remain were unclear (Wang W.Q. et al., 2020). Although many miRNAs are highly conserved among different plant species, their functions may be species-specific (Lenz et al., 2011). In addition, each plant also has its own species-specific miRNAs, which may have different expression characteristics and functions in the same physiological process (Jeong et al., 2013). Therefore, identification of conserved and species-specific miRNAs is of great significance for understand the molecular regulation mechanism of fruit development and ripening.

Mulberry trees are planted all over the world, including Europe, Africa, Asia, and America, and they have been used to feed silkworms for more than 5,000 years (Jia et al., 2014). Mulberry is not only the sole feed of the silkworm, but also is an important fruit tree with important economic and nutritional values. Mulberry fruits have long been used as edible fruits and traditional medicines not only for their special moderate sweet and sour taste, but also for they are rich in nutritive and bioactive compounds, such as vitamins, amino acids, fatty acids, resveratrol, rutin, quercetin, polysaccharides, and especially anthocyanin pigments (When et al., 2019). The mulberry fruit development is a complex biological process, which can be divided into three sequential phases: expansion, veraison, and maturation accompanied by obvious physiological changes. The color of the fruits changes from green to red and finally to purple, and the content of flavor volatiles increased, but the firmness decreased gradually (Lee and Hwang, 2017). Different from other fleshy fruits developed from the ovary of flowers, mulberry fruits are developed from the thickened perianth and ovary wall of female flower. Though it was suggested that mulberry fruits are probably climacteric, its respiratory type has not been determined (Leng et al., 2014). Therefore, the development mechanism of mulberry fruit may be different from other fruits. Unfortunately, there is little research that has been conducted to reveal the miRNA-mediated mechanisms of mulberry fruit development and ripening.

In this study, the small RNA libraries of mulberry fruits at different development stages were constructed and sequenced to investigate the miRNA expression profiles during mulberry fruit development and identify the specific miRNAs for mulberry fruit. A combination of bioinformatics analysis and experimental verification was used to prove the roles of miRNAs in the regulation of fruit development. The findings will facilitate to understand the regulatory network of miRNA underlying fruit ripening.

## Materials and Methods

### Plant Materials

Mulberry fruits represent different development stages, including MG, green fruits, 15 days after bloom (DAB) in fruit expanding stage; MR, red fruits, 30 DAB in fruit color changing stage; MP, purple fruits, 45 DAB in fruit mature stage, were collected from ‘Dashi’ (*Morus atropurpurea* Roxb) trees and frozen and stored in liquid nitrogen. Seedlings of *Arabidopsis thaliana* (Col-0) were grown in a growth chamber maintained on a 16 h: 8 h light: dark cycle at 22°C with the relative humidity of 50–60%.

### sRNA Library Construction, Sequencing and miRNAs Identification

Total RNAs were isolated from mulberry fruit samples using TRIzol reagents and then were separated on 15% denaturing polyacrylamide gel. After that the RNA bands (18–30 nt) were selected and purified, and the purified small RNAs were ligated with 5′- and 3′-RNA adapters and further selected by size-fractionation and amplified by PCR to produce sRNA libraries for high-throughput sequencing using an Illumina HiSeq 2000 platform by Beijing Genomics Institute (BGI) (Shenzhen, China). Three biological replicate experiments for different development stage mulberry fruits, respectively.

After sequencing, the raw sequencing tags were filtered to remove the low quality tags and reads smaller than 15 nt as well as the cellular structural RNAs. The clean reads obtained were subjected to BLASTn searches against the miRBase database (v21.0) to determine conserved miRNA. All the other unmapped reads were blasted against our local mulberry transcriptome database using BLASTX, and the sequences containing hairpin secondary RNA structures were used to predict novel miRNA candidates by MIREAP software, and when the MFEI (minimum folding free energy index) of the RNA was more than 0.85, the RNA was accepted as a precursor of miRNA.

### Analysis of the Differential Expression Profile of miRNAs

The frequency of each miRNA in the libraries was normalized and the fold-change between samples was calculated as described by previously (Gai et al., 2018). *P*-value < 0.05 and |log2 (fold change)| ≥ 1 was regarded as a threshold for defining significantly differential expression.

### Prediction and Analysis of Target Gene Ontology and KEGG Pathways

The candidate target genes of DE miRNAs were predicted based on the mulberry fruit transcriptome dataset using the psRNATarget web server^1^. KEGG pathways analysis and Gene ontology annotation of the DE miRNA target gene candidates were performed using Blast2GO program.

### Target Validation of mul-miR477 Using 5′-RNA-Ligase-Mediated Rapid Amplification of cDNA Ends (5′-RLM-RACE)

Total RNAs extracted from mulberry fruits prepared as above was ligated to the RNA oligo adapter (5′-CGACUGGAGCACGAGG ACACUGACAUGGACUGAAGGAGUAGAAA-3′) and then was reverse transcribed to prepare cDNA template using the GeneRacer Kit (Invitrogen, United States) following the manufacturer’s instructions. The cDNA obtained was used for PCR-amplification with GeneRacer 5′ primer (5′-CGACT GGAGCACGAGGACACTGA-3′) and the specific outer primer (5′-GAAGAGGAGCATAACTCAAGCC-3′). The PCR product obtained was used for nested PCR amplification with GeneRacer 5′ nested primer (5′-GGACACTGACATGGACTG AAGGAGTA-3′) and the specific inner primer (5′-GATGTA AAATCCGCCATTGCTG-3′). The final PCR product was purified and cloned into the pMD18-T vector (Invitrogen, United States) for sequencing.

### Quantitative Real-Time PCR Analysis

Total RNAs of each sample were isolated using TRIzol^®^ reagent (Invitrogen) and then treated with DNase I. Quantitative real-time PCR (qRT-PCR) analyses for the selected mRNAs and miRNAs were performed using the SYBR^®^ *Premix Ex Taq*^TM^ kit (TaKaRa) and PrimeScript^TM^ miRNA qPCR starter kit ver. 2.0 (TaKaRa), respectively, following the manufacturer’s protocol on a Rotor-Gene 3000A system (Applied Biosystems, United States). The *ACTIN* and *U6* genes were amplified as internal references for mRNA and miRNA analysis, respectively. The relative expression levels of mRNAs or miRNAs were calculated according to the 2^–ΔΔCT^ method (Livak and Schmittgen, 2001). All PCR reactions were performed three times for each sample. All measurements were performed by three biological replicates and three technical replicates. The primers used for qRT-PCR are given in the Supplementary Tables 3, 4.

### Production of Transgenic Plant Lines and Hybrid Seedlings

Genomic DNA of ‘Dashi’ mulberry was isolated by CTAB (cetyltrimethyl-ammonium bromide) protocol (Zhang et al., 2013), and the mul-miR477-producing DNA region was cloned with the primers (Forward primer: 5′-GTTTCAAGGCAT TGGTTACTG-3′; Reverse primer: 5′-GTCTCCGGTATTGCC TGAAG-3′). The cDNA was synthesized with the total RNAs isolated above, and the *Mul-ABCB19* and *Mul-ABCB19AS* genes were cloned with the primers (For *Mul-ABCB19*: Forward primer: 5′-CAATGGCGGAGAGCGCAGAG-3′, Reverse primer: 5′-TTATCTCAAATGTGATGGTGTT-3′; For *Mul-ABCB19AS*: Forward primer: 5′-TTGGGCATTGAAGGCAGCAACT-3′, Reverse primer: 5′-AATGGCATTTTGAACAGCAACTC-3′). The genes cloned were subcloned into the binary vector pBI121 individually, and then *Agrobacterium tumefaciens* strain GV3101 was transformed with the constructed vectors and introduced into wild type Arabidopsis using floral-dip method. Transformants were screened and verified by PCR technology, and the homozygotes of T2-generations of independent transgenic lines were randomly chosen for subsequent experiments. For hybridization of *Arabidopsis thaliana*, anthers were collected from the male parent and smeared on the stigma of female parent with stamens removed. After pollination, the plant was covered for 24 h to maintain high air humidity. When the seeds are mature, they are harvested and screened on the medium and then identified by PCR.

### Production of Mulberry Plants With Transgenic Hairy Roots

The *Mul-MIR477* genes were cloned into the binary vector pROK2 (driven by the CaMV35S promoter) to produce vector *35S:Mul-MIR477*, and then the vector *35S:Mul-MIR477* was introduced into *A. rhizogenes* K599 strains, which were responsible for the development of hairy roots of susceptible mulberry seedlings. Two-week-old Guisang You 62 mulberry seedlings with the first two fully expanded true leaves were used for agro-infiltration as described by previously (Meng et al., 2019). The seedlings injected with *A. rhizogenes* strains harboring empty vectors pROK2 were used as control group (CK). All the seedlings were cultured in a growth chamber maintained on a 16 h: 8 h light: dark cycle at 25°C with the relative humidity of 50–60%. After about 4 weeks, the well-developed hairy roots were detected by PCR and the original roots were cut off.

### Determination of Anthocyanin

Samples were extracted overnight with the methanol solution consisting of 0.1% (v/v) HCl at room temperature. Then the solution was centrifuged at 15,000 *g* for 2 min and passed through a 0.2-μm filter. The supernatant was collected and the anthocyanin content was determined according to the pH differential method (Ge et al., 2018). Absorbance was measured at 510 and 700 nm and anthocyanin content (mg⋅g^–1^ FW) was calculated as [(A_543_ – A_700_) _pH 1.0_ – (A_543_ – A_700_) _pH 4.5_ × 465 × 10]/(29000 × 1). Data are reported as means for at least three replications.

## Results

### Sequence Analysis of miRNA Libraries of Mulberry Fruits

**FIGURE 1:**
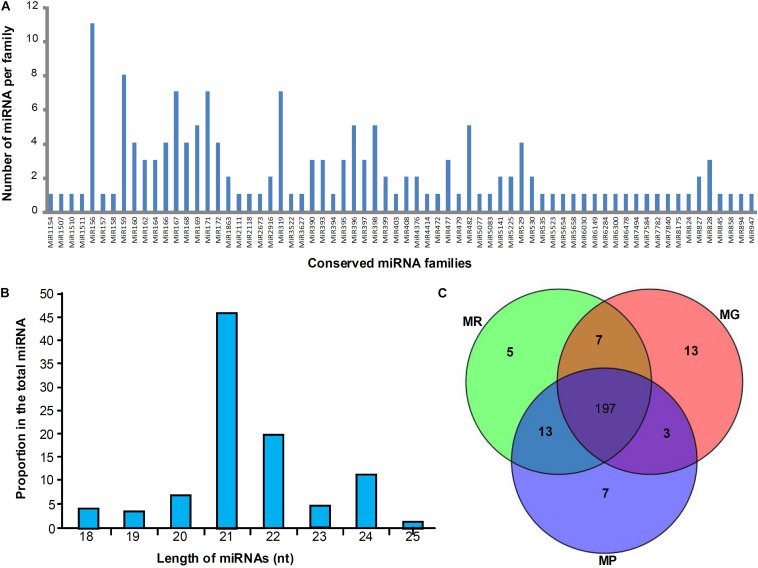
Distribution of conserved miRNAs in mulberry fruits. **(A,B)** Shows the number of miRNA members of different miRNA families and their length distribution, respectively, and **(C)** shows the Venn diagrams of miRNAs in the libraries of MG, MR, and MP.

Three miRNA libraries (MG, MR, and MP) were constructed using the samples from three different development stages to explore the expression profiles of miRNAs during mulberry fruit development and ripening, and 159 conserved miRNA members belonging to 68 families were identified. Among the 68 miRNA families, miR156 family containing 11 members is the largest, followed by miR159 family with 8 members, and the miR167, miR171 and miR319 families all containing seven members belonged to the third largest family (Figure 1A). It was found that the conserved miRNAs identified in length ranges from 18 to 25 nt, and the 21-nt miRNAs take the majority (46.51%), followed by the 22-nt (19.77%), 24-nt (11.63%), 20-nt (6.98%), 23-nt (5.43%), 18-nt (4.26%), 19-nt (3.49%), and 25-nt (1.94%) miRNAs (Figure 1B). To predict the novel miRNAs in mulberry fruits, all non-annotated sRNAs were mapped to our local mulberry transcriptome database, and 86 candidate novel miRNAs (designated mul-miRn01–mul-miRn86), which may be specific to mulberry were identified (Supplementary Table 1). Among the novel miRNAs, 25 were identified with complementary miRNA^∗^s. It was found that the novel miRNAs identified usually showed a very weak expression levels compared with the conserved miRNAs. Of the 86 novel miRNA candidates, only 9 miRNAs have been sequenced more than 500 times. Among all the miRNAs identified, 197 miRNAs were detected in all the libraries, whereas 13, 5, and 7 miRNAs were detected only in MG, MR, and MP library, respectively, suggesting that they might have specific roles in regulation of fruit ripening (Figure 1C).

### miRNA Expression Profiles During Mulberry Fruit Development and Ripening

**FIGURE 2:**
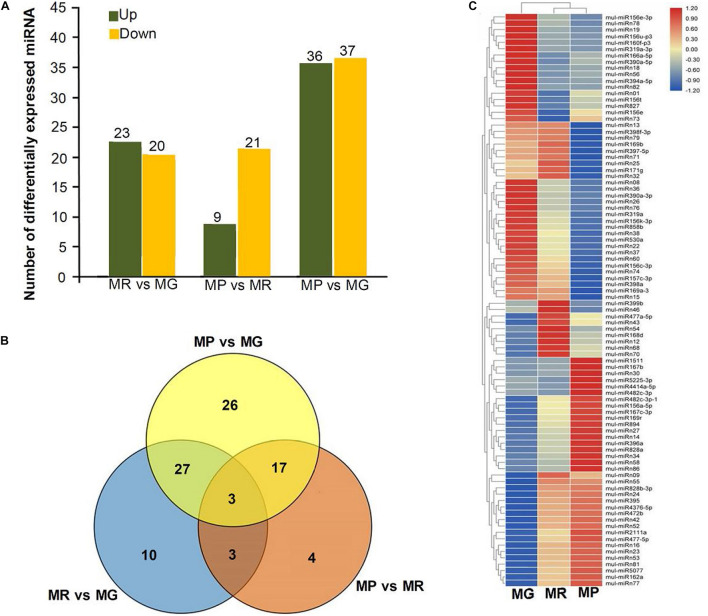
Statistical analysis of DE miRNAs. **(A)** Numbers of DE miRNAs between MR vs. MG, MP vs. MR, and MP vs. MG. **(B)** Venn diagrams of common and specific DE miRNAs among MR vs. MG, MP vs. MR, MP vs. MG, and MG vs. MR vs. MP. **(C)** Heat map of the normalized expression level of the DE miRNAs during ripening process. Heatmap shows the up- and down-regulated miRNAs in MG, MR, and MP stages of mulberry fruit. Scale represents log2 transformed expression value. Each column is a stage, while each row represents a miRNA. Red to blue indicates that the expression level is from high to low. miRNAs showing a similar developmental profile are clustered together.

**FIGURE 3:**
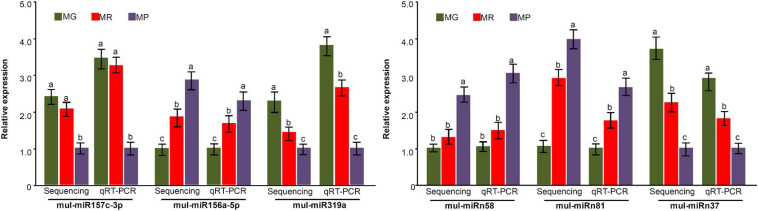
miRNA expression level validation by qRT-PCR. Three biological replicates per treatment were assayed and three technical replicates were performed for each sample. The error bars represent standard errors, and the different letters above the columns indicate statistically significant differences (*P* < 0.05) according to Duncan’s multiple range test.

To explore the miRNA expression profiles during mulberry fruit development and ripening, the counts of reads generated by high-throughput sequencing were used to analyze the differential expression of miRNAs. A total of 90 miRNAs were found to be differentially expressed (DE) by comparing the expression levels of miRNAs between every two libraries of MG, MR, and MP (Table 1). Compared with the fruits at MG, there were 23 miRNAs up-regulated and 20 down-regulated in the fruits at MR, and 36 miRNAs up-regulated and 37 down-regulated in the fruits at MP. While compared with the fruits at MR, there were 9 miRNAs up-regulated and 21 down-regulated in the fruits at MP (Figure 2A). The Venn diagram showed that the miRNAs identified in the fruits at different stages were highly overlapped. In the three pairwise comparisons, only 40 DE miRNAs were specific to one of them, including 10 DE miRNAs between MR and MG, 26 DE miRNAs between MP and MG, and 4 DE miRNAs between MR and MP (Figure 2B). In order to explore the associations between these DE miRNAs, hierarchical clustering analysis was performed on them according to their expression patterns. DE miRNAs with the same or similar expression profiles were clustered together (Figure 2C). The results showed that these DE miRNAs were divided into two categories: the ones with higher expression in MG and MR, and the ones highly expressed in MP. At the same time, it was also observed that some members of the same miRNA family showed different expression profiles. For example, mul-miR156e was expressed at the highest level in the MG, while mul-miR156a was expressed at the highest level in the MP. These results suggested that even members of the same miRNA family might have diverse functions during development and ripening. To validate differential expression pattern of the identified miRNAs, six miRNAs including the up-regulated and down-regulated miRNAs during fruit development and ripening were randomly selected for analysis by qRT-PCR. As shown in Figure 3, all the qRT-PCR results were quite consistent with the results of high-throughput sequencing, which confirmed that the miRNA profiles detected by high-throughput sequencing are reliable.

**TABLE 1:** Expression profile of differentially expressed miRNAs during mulberry fruit ripening process.

**miRNA_name**	**miRNA sequence**	**Normalized reads**			***P*-value**
		**MG**	**MR**	**MP**	
mul-miR1511	ACTTAGCTCTGATACCATG	0	2	34	1.48E–11
mul-miR156a-5p	TTGACAGAAGAGAGTGAGCACTT	48	96	144	1.11E–05
mul-miR156c-3p	GCTCACTGCTCTTTCTGTCAGC	447	305	77	0.00E+00
mul-miR156e	CTTGACAGAAGAGAGAGAGCAC	66	28	49	8.02E–08
mul-miR156e-3p	GTATGCTATTGCTTTTGCGTT	5	1	0	2.32E–01
mul-miR156k-3p	GCTTTCTCTTCTTCTGTCAGC	179	88	25	8.57E–45
mul-miR156t	TTGACAGAAGAGAGAGAGCAC	5,764	2,824	3,765	0.00E+00
mul-miR156u-p3	TGGGAGTGTGCTTTCTCTTCTTCT	12	1	0	3.86E–07
mul-miR157c-3p	GCTCTCTATGCTTCTGTCATCC	89	75	36	1.74E–10
mul-miR160f-p3	TTGGCATGAGGGGAGTCATGC	68	27	24	2.18E–13
mul-miR162a	TCGATAAACCTCTGCATCCAGTT	134	411	548	5.12E–29
mul-miR166a-5p	GAATGTTGTCTGGCACGAGGT	10	4	5	2.81E–02
mul-miR167b	TGAAGCTGCCAGCATGATCTGC	855	1,032	3,203	0.00E+00
mul-miR167c-3p	GATCATGTGGTAGTTTCACC	39	99	161	5.81E–10
mul-miR168d	TCGCTTGGTGCAGGTCGGGAATT	0	7	2	3.39E–02
mul-miR169a-3	CGGCAAGTTGTTCTTGGCTAC	11	10	2	8.42E–03
mul-miR169b	CAGCCAAGGATGACTTGCCGC	33	40	10	7.75E–06
mul-miR169r	TGAGCCAAGAATGACTTGCCGA	105	167	241	2.96E–05
mul-miR171g	CGAGCCGAATCAATATCACTC	60	73	27	3.89E–07
mul-miR2111a	TAATCTGCATCCTGAGGTTTA	15	38	54	4.63E–03
mul-miR319a	TTGGACTGAAGGGAGCTCCTC	1,013	663	442	0.00E+00
mul-miR319a-3p	TTGGACTGAAGGGAGCTCCCT	206	59	47	5.65E–56
mul-miR390a-3p	CGCTATCTATCCTGAGTTTCA	185	118	93	8.70E–20
mul-miR390a-5p	AAGCTCAGGAGGGATAGCGCC	1,062	466	584	0.00E+00
mul-miR394a-5p	TGGAATTCTGTCCACCTCC	92	43	45	7.63E–13
mul-miR395	TGAAGTGTTTGGGGGAACTCC	22	52	59	3.56E–02
mul-miR396a	CACAGCTTTCTTGAACTT	16	27	49	5.54E–03
mul-miR397-5p	TCATTGAGTGCAGCGTTGATG	429	476	214	1.35E–38
mul-miR398a	TGTGTTCTCAGGTCGCCCCTG	211	163	40	2.71E–40
mul-miR398f-3p	CATGTGTTCTCAGGTCGCCCC	12	13	1	1.24E–03
mul-miR399b	TGCCAAAGGAGAATTGCCCTG	2	8	1	1.81E–01
mul-miR4376-5p	TACGCAGGAGAGATGACGCTGT	206	403	450	1.11E–06
mul-miR4414a-5p	AGCTGCTGACTCGTTGGTTCA	4	3	20	6.00E–04
mul-miR472b	TTTTCCCAACACCACCCA	52	185	217	2.32E–12
mul-miR477-5p	AACTCTCCCTCAAAGGCTTC	259	719	1001.154	1.06E–23
mul-miR477a-5p	ACTCTCCCTCAAGGGCTTC	295	661	474	2.67E–15
mul-miR482c-3p	TCTTTCCGAGACCTCCCATACC	5	3	32	1.80E–07
mul-miR482c-3p-1	TTCCCAAGGCCGCCCATTCC	136	247	351	3.04E–09
mul-miR5077	TTCCCGTCGGGTTCACCA	12	31	41	2.53E–02
mul-miR5225-3p	TCATCTCTCCTCGACTGAAGC	28	25	54	8.17E–03
mul-miR530a	AGGTGCAGATGCAGATGCAGG	18	12	7	5.19E–03
mul-miR827	TTAGATGACCATCAACGAACT	14	7	9	3.11E–02
mul-miR828a	TCTTGCTCAAATGAGTATTCCATT	0	2	6	2.38E–01
mul-miR828b-3p	CATTTGAGCAAGCAATGTTAC	18	44	47	3.18E–02
mul-miR858b	TTCGTTGTCTGTTCGACCTTG	1,612	1,102	720	0.00E+00
mul-miR894	TTCACGTCGGGTTCACCA	1,651	3,109	5,100	0.00E+00
mul-miRn01	CAATAACTCCAAATTCAAGATT	15	5	9	1.11E–02
mul-miRn08	TTTGTAGTTGAATTTGAAGACA	2,326	1,323	845	0.00E + 00
mul-miRn09	TCTTCAATTCGACTACAAAGG	381	900	769	7.75E–19
mul-miRn12	TCCATTTGAAATTTGGCATGTTCT	9	68	26	6.05E–10
mul-miRn13	TTTAAATGGTAATCTTACAACG	10	10	4	2.09E–02
mul-miRn14	AAACACCAAGCTTCGTAGAGAGAGA	1	4	10	4.44E–02
mul-miRn15	CGGGCCTGGGAGGTTTGGTAGG	279	257	136	4.91E–25
mul-miRn16	TTCCAAATCCACCCATGCCCAC	6,242	15,957	19,433	0.00E+00
mul-miRn18	AATGATGTCGCATCTGGTGGG	23	10	11	5.62E–04
mul-miRn19	TTTGTGAGTGAATCTAAAGCA	46	14	10	5.85E–13
mul-miRn22	GGGATAAATCTCCATCGGGACAAA	245	164	96	4.00E–31
mul-miRn23	TAATGCGGGGATGGCGTGCTT	180	381	459	1.57E–10
mul-miRn24	TGTAAGCACGTCATCCCCGCA	35	73	79	2.47E–02
mul-miRn25	TGAGAATAGCTGACTGAACTGT	9	14	0	8.37E–04
mul-miRn26	ATAGGGTAGCGATTGCAGTTCAGT	12	5	2	7.41E–04
mul-miRn27	AATGGGCTGTTTGGGAAGAAAT	73	111	177	3.43E–05
mul-miRn30	TTTTTTATTCAGATTGCAATC	603	704	1,825	0.00E+00
mul-miRn32	CTAAAGGATGCAGAGGTGTGA	65	79	34	1.52E–06
mul-miRn34	CTTCTACCCTATCACATCTTTC	5	12	30	7.33E–04
mul-miRn36	AACACGACACATGACACGAATTGT	16	6	1	5.21E–06
mul-miRn37	AATGAGGTTTGATCCGAAATC	158	95	42	3.28E–28
mul-miRn38	CTTGTTTCAACATCAACGCACT	13	8	3	3.21E–03
mul-miRn42	CGAACTTTCCTCTTCCCGACC	66	153	180	2.61E–05
mul-miRn43	ATTGGTCGGGATGATGGAAGTTCG	21	52	37	3.68E–02
mul-miRn46	CACAGCGTTTTGCACGTGCCC	5	11	3	2.92E–01
mul-miRn52	TGACACAAGATCATCCTCCAA	1	31	40	2.76E–06
mul-miRn53	TTTGGAGGATGATCTTGTGTC	5	59	81	6.01E–10
mul-miRn54	TAGGATCCTGGTTGAGTCCCA	11	134	30	4.85E–28
mul-miRn55	CGACCTTTGCGTTGCCGCTTC	0	97	94	1.05E–15
mul-miRn56	AAACAGTGAATGTAGAGAGTGACA	22	4	6	2.42E–07
mul-miRn58	TTCGCCCCATTCATGATTAGA	47	64	124	1.00E–05
mul-miRn60	AAAATCATGATTTGAGTTAGTTTA	27	20	11	6.81E–04
mul-miRn68	AGCATCCTCGTGTGGCATATGGAT	17	41	26	4.95E–02
mul-miRn70	TGTTAGGATCCTGGTTGAGTC	49	700	299	0.00E+00
mul-miRn71	AATTTTTTGATACCAACTCACATG	17	19	6	5.79E–03
mul-miRn73	AAATTGACAGTTTGAGACATG	14	2	10	2.95E–03
mul-miRn74	ATGACAAATTACTGCGTTGAGTGA	23	19	11	6.78E–03
mul-miRn76	GGCGTGACCCCTGAGAACACAAG	143	55	19	2.05E–40
mul-miRn77	TTTCTCTATCGCTCTCCTCGTA	1	13	19	5.65E–03
mul-miRn78	ATTGACGGTTTGAGACACGAGATT	34	17	13	6.45E–06
mul-miRn79	GCTCGCTACTCTTTCTGTCAGT	1,155	1,241	255	0.00E+00
mul-miRn81	GACAAGAAGATCAAGATCGGAATC	9	27	35	2.21E–02
mul-miRn82	AACGTCTTAGAATGAGTTCATGATC	20	8	8	2.60E–04
mul-miRn86	TGTAGAATCCATGGCTTCCT	7	12	29	5.58E–01

### Prediction and Analysis of the Target Genes of DE miRNAs

**FIGURE 4:**
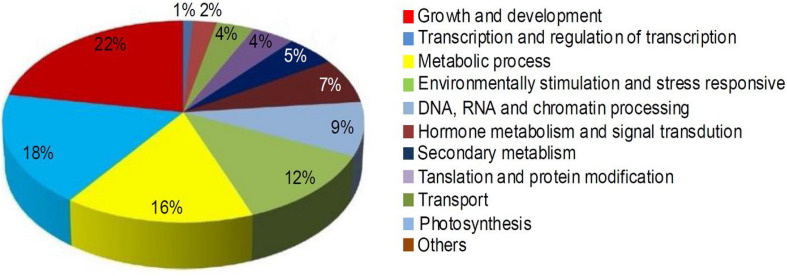
Percentage of the target genes of DE miRNAs in various categories.

To elucidate the roles of miRNAs in mulberry fruit ripening, the target genes of the DE miRNAs were predicted, and then Gene Ontology (GO) and KEGG analysis of the target genes was performed. A total of 119 potential target genes of 36 conserved DE miRNAs and 68 potential target genes of 34 novel DE miRNAs were identified. The ontologies and KEGG functional annotations of the predicted target genes were given in Supplementary Table 2, and the target genes were categorized into 11 classes based on their ontologies and KEGG functional annotations (Figure 4). The first category target genes of the DE miRNAs were involved in growth and development processes. Meanwhile, a large proportion of the target genes predicted were associated with transcription and regulation of transcription, and metabolic process. The other categories included environmental stimulation and stress responsive, hormone metabolism and signal transduction, secondary metabolism, and so on. It is worth noting that many miRNAs such as mul-miR156e, mul-miR319a, and mul-miR396a had multiple putative target genes. At the same time, there were some genes targeted by multiple miRNAs. These results indicated that the DE miRNAs during mulberry fruit ripening may be associated with a variety of biologic processes, and their regulatory networks involved in the ripening process were intricate.

**FIGURE 5:**
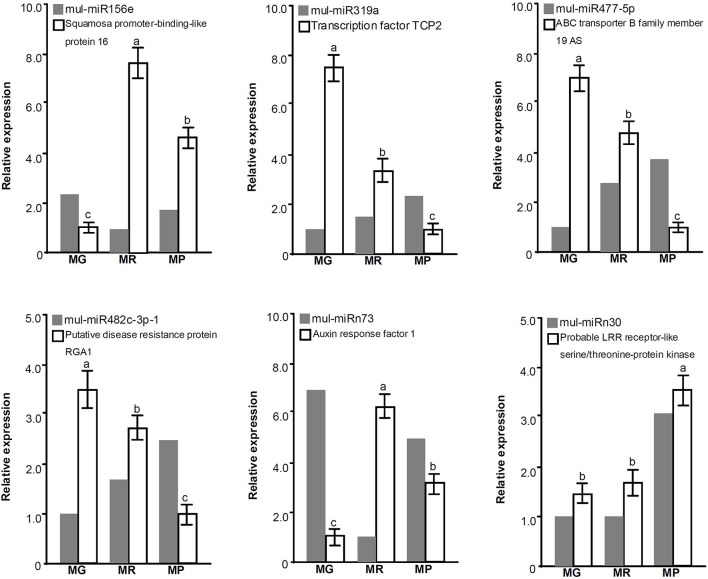
Expression analysis of the DE miRNAs and their target genes during mulberry fruit ripening. Actin is used as the internal control for target gene expression level assays. Three biological replicates per treatment were assayed and three technical replicates were performed for each sample. The error bars represent standard errors, and the different letters above the columns indicate statistically significant differences (*P* < 0.05) according to Duncan’s multiple range test.

To validate the predicted target genes of DE miRNAs, the expression profiles of some target genes during fruit ripening were also investigated (Figure 5). The qRT-PCR results showed that the expression trends of mul-miR156e, mul-miR319a, mul-miR477-5p, mul-miR482C-3p, and mul-miRn73 were opposite to those of their target genes, suggesting that these miRNAs may guide the cleavage of those target genes and repress their expressions. However, the mul-miRn30 and its target gene, the probable LRR receptor-like serine/threonine-protein kinase gene, showed the same expression patterns, suggesting that it may not be cleaved by mul-miRn30 or its expression is determined by translational repression and/or by multiple miRNAs.

### Silencing of the Antisense lncRNA of ABC Transporter B Family Member 19 by mul-miR477

**FIGURE 6:**
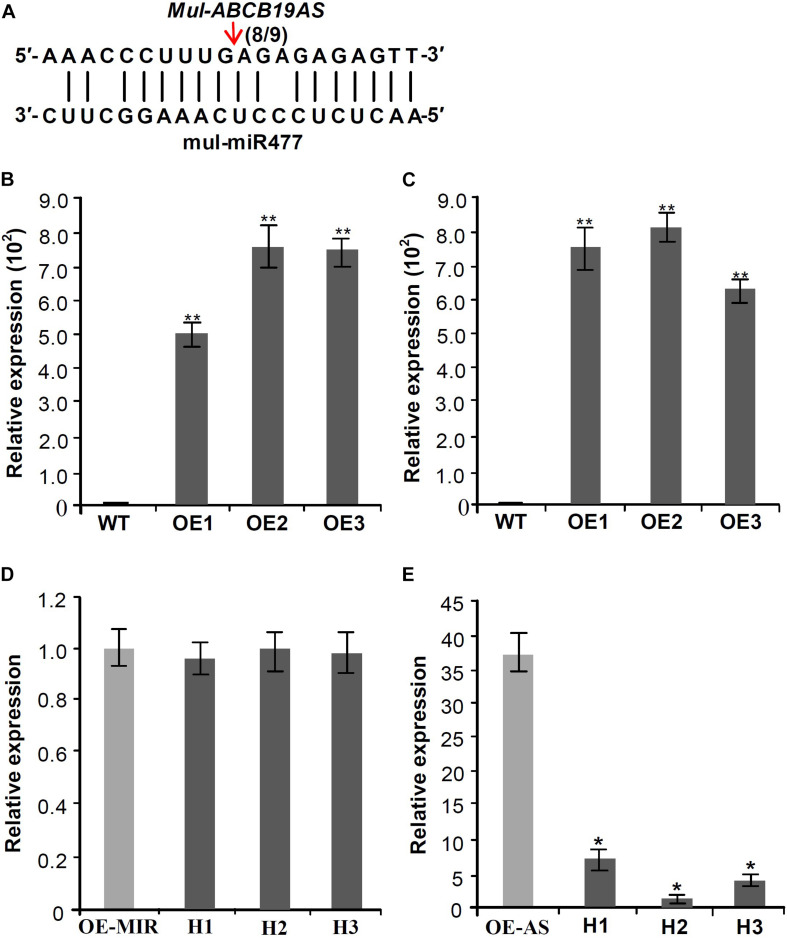
Validation of the silencing of *Mul-ABCB19AS* by mul-miR477. **(A)** Mapping of the cleavage sites of *Mul-ABCB19AS* using 5′RLM-RACE. **(B,C)** qRT-PCR analysis of the expressions of mul-miR477 in the transgenic *Mul-MIR477* Arabidopsis plants **(B)** and *Mul-ABCB19AS* in the transgenic *Mul-ABCB19AS* Arabidopsis plants **(C)**. **(D,E)** Expression analysis of mul-miR477 **(D)** and *Mul-ABCB19AS* **(E)** in the hybrid plants of transgenic *Mul-MIR477* and *Mul-ABCB19AS* plants by qRT-PCR. Arrow indicates the cleavage sites of mul-miR477 on *Mul-ABCB19AS*, and the number indicates the number of clones corresponding to the cleavage site. The *ACTIN* and *U6* genes were amplified as reference genes, respectively. Three biological replicates per treatment were assayed and three technical replicates were performed for each sample. Values are expressed as mean ± SD. Double asterisks indicate significant difference (*P* < 0.01) according to Student’s *t*-test. WT represents wild type Arabidopsis, and OE1-3 represent different transgenic lines. OE-MIR and OE-AS indicate the transgenic *Mul-MIR477* and *Mul-ABCB19AS* plants, respectively. H1-3 indicates different hybrid plants of transgenic *Mul-MIR477* and *Mul-ABCB19AS* plants.

Target prediction of the DE miRNAs indicated that mul-miR477 could target the antisense long non-coding RNA (designed as *Mul-ABCB19AS*) of the ATP binding cassette (ABC) transporter B 19 gene (designed as *Mul-ABCB19*). Furthermore, 5′-RLM-RACE was used to verify the mul-miR477-guided cleavage of *Mul-ABCB19AS*, and the result showed that *Mul-ABCB19AS* was cleaved by mul-miR477 at the 11th positions (Figure 6A). The ABCB19 protein, also known as multidrug resistance protein (MDR1) and *P*-glycoprotein 19 (PGP19), has been reported to be involved in auxin transport in Arabidopsis (Okamoto et al., 2016). In addition, the manipulation of expression of *ABCB19* was reported to affect the expression of the chalcone synthase (*CHS*) gene, which is a light-responsive gene and required for normal accumulation of anthocyanin in Arabidopsis (Lin and Wang, 2005). The sequence alignment showed that the amino acid sequences of Mul-ABCB19 shared 90.42% identity with AtMDR1 (Supplementary Figure 1), indicating that Mul-ABCB19 might be an ortholog of AtABCB19 and could be involved in the auxin transport and anthocyanin accumulation. To explore whether mul-miR477 can target *Mul-ABCB19AS*, the *Mul-ABCB19AS*, and *Mul-MIR477* genes were cloned and transformed into wild type Arabidopsis, independently. The results of PCR of genomic revealed that the *Mul-ABCB19AS* and *Mul-MIR477* genes were integrated into the genome of Arabidopsis, respectively (Supplementary Figures 2A,B). In addition, qRT-PCR results showed that Mul-ABCB19AS transcript and mature mul-miR477 were successfully expressed in the transgenic Arabidopsis plants (Figures 6B,C). Then the hybrid plants (designed as *Mul-ABCB19AS* × *Mul-MIR477*) between *Mul-ABCB19AS* and *Mul-MIR477* transgenic Arabidopsis were generated, and the PCR of genomic revealed that the *Mul-ABCB19AS* and *Mul-MIR477* genes were integrated into the genome of the hybrid plants (Supplementary Figures 2C,D), and qRT-PCR analysis results showed that the difference of the expression level of mul-miR477 between the *Mul-MIR477* transgenic and the hybrid plants was not statistically significant (Figure 6D). However, it was observed that the level of *Mul-ABCB19AS* in the *Mul-ABCB19AS* × *Mul-MIR477* plants was significantly lower than that in the plant overexpressing *Mul-ABCB19AS* (Figure 6E). These results indicated that mul-miR477 can target and cleave Mul-ABCB19AS transcript in the *Mul-ABCB19AS* × *Mul-MIR477* plants.

### *Mul-ABCB19AS* Suppresses the Expression of *Mul-ABCB19* to Modulate Anthocyanin Accumulation

**FIGURE 7:**
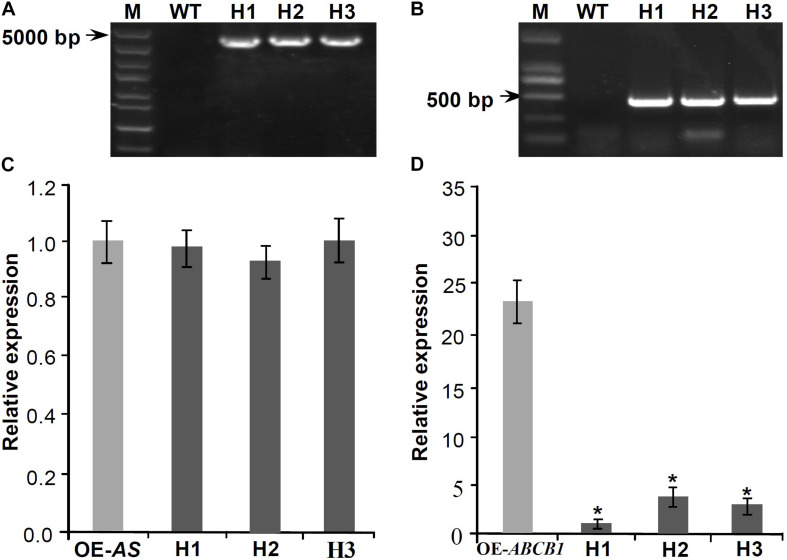
Expression analysis of *Mul-ABCB19AS* and *Mul-ABCB19* in the transgenic *Mul-ABCB19AS* or *Mul-ABCB19* plants and their hybrid plants. **(A,B)** Genome PCR detections of *Mul-ABCB19AS* **(A)** and *Mul-ABCB19* **(B)** genes in the hybrid plant genome. **(C,D)** Expressions analysis of *Mul-ABCB19AS* **(C)** and *Mul-ABCB19* by qRT-PCR **(D)**. The *ACTIN* gene was amplified as reference gene. Three biological replicates per treatment were assayed and three technical replicates were performed for each sample. Values are expressed as the mean ± SD, and the double asterisks indicate significant difference (*P* < 0.01) according to Student’s *t*-test. WT indicates wild type Arabidopsis, and H1-3 indicates hybrid lines of transgenic *Mul-ABCB19AS* and *Mul-ABCB19* plants. OE-AS and OE-ABCB19 indicate the transgenic *Mul-ABCB19AS* and *Mul-ABCB19* plants, respectively.

To analyze whether *Mul-ABCB19AS* can inhibit the expression of *Mul-ABCB19* gene, the transgenic *Mul-ABCB19AS* plants were hybridized with the transgenic *Mul-ABCB19* plants to produce the hybrid plants (designed as AS × 19). Genomic PCR results showed that the *Mul-ABCB19AS* and *Mul-ABCB19* were inserted into the genomes of Arabidopsis hybrid plants, respectively (Figures 7A,B). In addition, qRT-PCR analysis showed that the expression level of *Mul-ABCB19AS* between the transgenic *Mul-ABCB19AS* and hybrid plants was not significantly differential (Figure 7C), whereas the level of *Mul-ABCB19* in the AS × 19 plants was significantly lower than that in the *Mul-ABCB19* transgenic plants (Figure 7D). These results indicated that *Mul-ABCB19AS* can suppress the expression of *Mul-ABCB19* in the hybrid plants.

**FIGURE 8:**
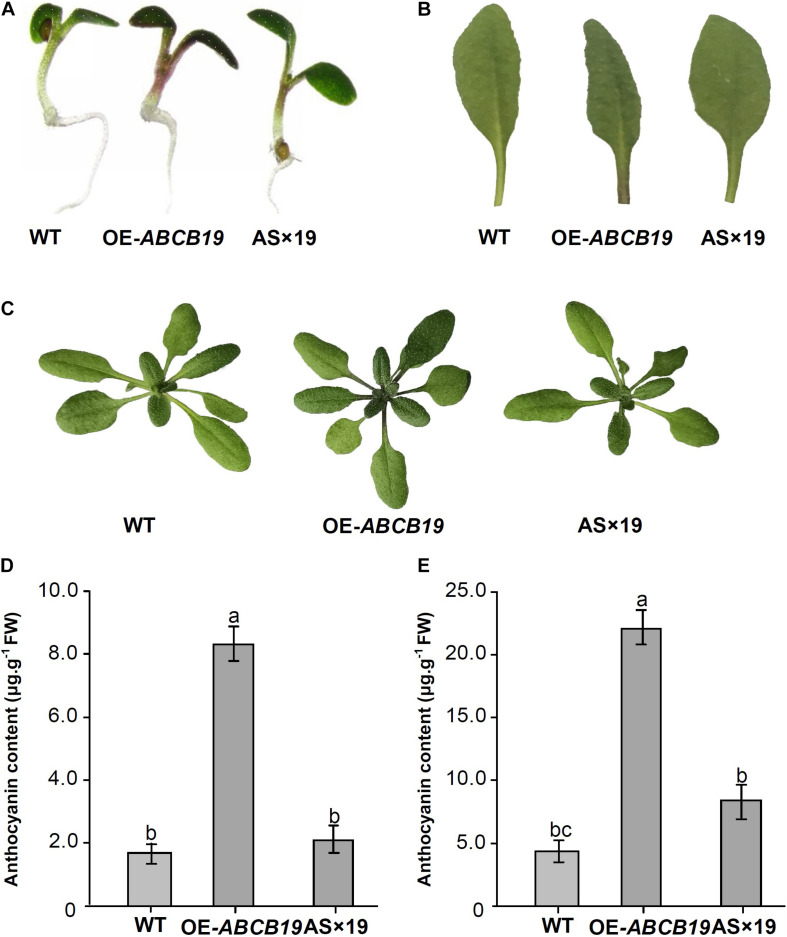
Phenotypes and anthocyanin content of the transgenic *Mul-ABCB19AS* and AS × 19 hybrid Arabidopsis plants. **(A)** Phenotypes of 1-week-old seedlings. **(B)** Accumulation of anthocyanin in the base site of petioles. **(C)** Phenotypes of 4-week-old seedlings. **(D,E)** Anthocyanin contents of 1-week-old **(D)** and 4-week-old seedlings **(E)**. The values are expressed as the mean ± SD, Significant differences by Duncan’s multiple range test (*P* < 0.05) are indicated by different letters.

Phenotypic analysis of transgenic *Mul-ABCB19*, AS × 19, and wild type Arabidopsis plants showed that there was obvious anthocyanin accumulation at the cotyledon–hypocotyl junction of 1-week-old transgenic *Mul-ABCB19* plants and at the base of the petioles of rosette leaves and the junction of petiole and stem of 4-week-old transgenic *Mul-ABCB19* plants, but there was no obvious anthocyanin accumulation in the same tissues of the wild type and AS × 19 seedlings (Figures 8A–C). Moreover, the anthocyanin accumulation levels of the transgenic *Mul-ABCB19* plants were significantly higher than those of AS × 19 hybrid and wild type plants (Figures 8D,E). These results indicated that overexpressing *Mul-ABCB19* confers the transgenic plants more anthocyanin accumulation, and *Mul-ABCB19AS* might modulate anthocyanin accumulation by suppressing the expression of *Mul-ABCB19* in the plants. These results indicated that mul-miR477 acts as a positive regulator in anthocyanin accumulation through the regulatory network of mul-miR477—*Mul-ABCB19AS*—*Mul-ABCB19*.

### Overexpression of *Mul-MIR477* Gene Enhances Anthocyanin Accumulation in Mulberry Hairy Roots

**FIGURE 9:**
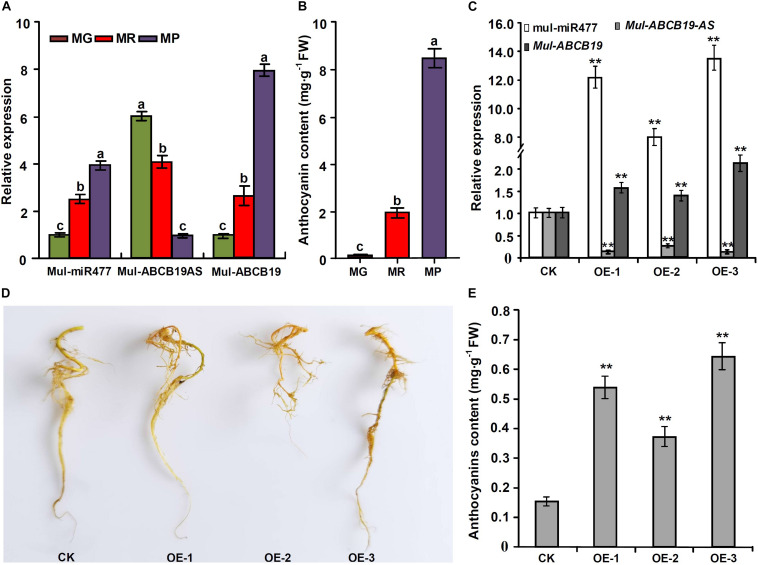
Expression levels of mul-miR477, *Mul-ABCB19AS* and *Mul-ABCB19* and anthocyanin content in the mulberry fruits at different development stages and the transgenic *Mul-MIR477* hairy roots. **(A)** qRT-PCR analysis of the expression levels of mul-miR477, *Mul-ABCB19AS*, and *Mul-ABCB19* in mulberry fruits at different development stages. **(B)** Anthocyanin content in mulberry fruits at different development stages. **(C)** qRT-PCR analysis of the expression levels of mul-miR477, *Mul-ABCB19AS* and *Mul-ABCB19* in mulberry hairy roots. **(D)** Phenotypes of the transgenic *Mul-MIR477* hairy roots. **(E)** Anthocyanin content in the transgenic *Mul-MIR477* and CK hairy roots. The expression levels are normalized to *U6* or *ACTIN* gene, and the values are expressed as the mean ± SD, and the double asterisks indicate significant difference (*P* < 0.01) according to Student’s *t*-test. MG, MR, and MP indicate green fruits, red fruits, and purple fruits, respectively. CK indicates transgenic empty vectors hairy roots, and OE1-3 indicates the transgenic *Mul-MIR477* hairy root lines.

To confirm the regulatory network of mul-miR477—*Mul-ABCB19AS*—*Mul-ABCB19* in mulberry, the correlation between the expression levels of mul-miR477, *Mul-ABCB19AS*, and *Mul-ABCB19*, as well as the anthocyanin content in mulberry fruits at MG, MR, and MP stages were detected. The results showed that the expression level of mul-miR477 was increased with mulberry fruit ripening, whereas the expression level of *Mul-ABCB19AS* was decreased in the process. This suggested that there was a very good negative correlation between their expression levels. However, the expression level of *Mul-ABCB19* and the anthocyanin content were increased with mulberry fruit ripening (Figures 9A,B). Therefore, the express profile of mul-miR477, *Mul-ABCB19AS*, and *Mul-ABCB19* during mulberry fruit ripening and their correlation with anthocyanin accumulation confirmed that the regulatory network of mul-miR477—*Mul-ABCB19AS*—*Mul-ABCB19* in anthocyanin accumulation.

At present, the efficient genetic transformation and regeneration system of mulberry has not been established, to verify the results obtained above, the transgenic *Mul-MIR477* mulberry hairy roots were generated. qRT-PCR showed that the mature mul-miR477 was highly expressed in the transgenic *Mul-MIR477* hairy roots. In addition, it was observed that the expression level of *Mul-ABCB19AS* in the transgenic *Mul-MIR477* hairy roots was significantly lower than that in the transgenic empty vectors pROK2 hairy roots, while the expression level of *Mul-ABCB19* was significantly higher than that in the transgenic empty vectors pROK2 hairy roots (Figure 9C). These results confirmed that mul-miR477 can target and cleave the transcript of *Mul-ABCB19AS* which can repress the expression of *Mul-ABCB19*. Phenotypically, the colors of transgenic *Mul-MIR477* hairy roots were light red, while those of transgenic empty vectors pROK2 hairy roots were yellow (Figure 9D). As expected, the anthocyanin content of transgenic *Mul-MIR477* hairy roots was significantly higher than that of transgenic empty vector hairy roots (Figure 9E). These results were consistent with those obtained in *Arabidopsis thaliana*. Therefore, mul-miR477 participates in anthocyanin accumulation as a positive regulator through the regulatory network of mul-miR477—*Mul-ABCB19AS*—*Mul-ABCB19*.

## Discussion

Mulberry fruit is fleshy berry fruits widely recognized for their flavor, nutritive and bioactive compounds (Lee and Hwang, 2017), and its ripening is regulated by various endogenous and exogenous factors (Mahmood et al., 2017). It is well documented that miRNAs are key regulators in fruit development and ripening (Zeng et al., 2015). However, the regulatory roles of miRNAs in mulberry fruit development and ripening has not been characterized yet. In this study, the miRNAs involved in mulberry fruit ripening were identified by small RNA sequencing, and a total of 159 conserved miRNAs belonging to 68 families and 86 novel miRNAs were identified. Among of these miRNAs identified, there were 90 miRNAs differentially expressed at different fruit development stages (Table 1). These results suggested that miRNAs mediate the complex regulatory network of mulberry fruit development and ripening.

It has been reported that plant hormones are the key regulators of fruit development and ripening (Fenn and Giovannoni, 2021), and auxin plays an important role in regulating the expansion of fruit cells and triggering the onset of ripening in various fruits (Yue et al., 2020). In this study, the mul-miRn73 was predicted to target the auxin regulator auxin response factor 1, which was proposed to be served as a transcription factor of auxin-responsive genes (Wang W.Q. et al., 2020; Wang Y.F. et al., 2020). Our data showed that the expression of mul-miRn73 was up-regulated during fruit development (Table 1), indicating that the signaling pathway of auxin might be inhibited during mulberry fruit ripening. Ethylene is another important phytohormone, which plays vital roles during fruit ripening (Liu et al., 2015; Li et al., 2019). Ethylene insensitive 3(EIN3)-binding F-box 1 can repress the effect of ethylene by directing the degradation of EIN3 which is a key positive regulator of ethylene signal transduction (Qiu et al., 2015). In this study, EIN3-binding F-box 1 was predicted to be the target gene of mul-miR828b-3p which accumulation was up-regulated with fruit ripening. In addition, one of the negative regulators of ethylene mediated signaling pathway, ethylene response sensor 1 was predicted as the target of mul-miRn86 which accumulation was also up-regulated with fruit ripening. These results suggested that the ethylene signal transduction pathway might be promoted during mulberry fruit ripening. Since auxin delays the fruit ripening and ethylene promotes fruit ripening (Böttcher et al., 2013), it was proposed that mul-miR828b-3p, mul-miRn73, and mul-miRn86 might promote fruit ripening by suppressing the auxin signaling pathway and promoting ethylene signaling pathway. In addition, some miRNAs such as mul-miR156e, mul-miR156t, mul-miR394a-5p, mul-miR894, and mul-miRn42 were predicted to target the genes involved in cytokinin or abscisic acid signaling pathway. Therefore, these miRNAs may play roles in controlling fruit development and ripening visa mediating hormonal crosstalk.

During fruit development, miRNAs play pivotal roles in controlling cell proliferation, expansion and differentiation. It was reported that growth-regulating factors (GRFs) play an important role in regulating fruit growth (Cao et al., 2016). In this study, it was found that the *GRF1* gene was predicted to be targeted by mul-miR396, and the expression level of mul-miR396 was increased while the expression level of *GRF1* was decreased as fruit ripening (Table 1 and Supplementary Table 2). In addition, some target genes of the DE miRNAs (mul-miR1511, mul-miR156e, mul-miR156t, and mul-miR5077) were also involved in cell division and cell growth. These miRNAs possibly act as regulators to adjust the expression of their target genes and control fruit development and ripening. Fruit softening is a sign of fruit ripening, which involves the changes of cell wall composition, structure and biochemical characteristics (Paniagua et al., 2017). In this study, the predicted target genes of some DE miRNAs (mul-miR156e-3p, mul-miR397-5p, mul-miR894, and mul-miRn52) might be associated with the formation and structure modification of cell wall (Supplementary Table 1). Therefore, these DE miRNAs possibly play important roles in controlling fruit firmness and softening during fruit ripening.

The quality and flavor of fruit are mainly determined by the content and composition of sugar and acid in fruit, and these are a variety of sugar and acid metabolic changes in the fruit ripening process (Lado et al., 2018). KEGG analysis of some target genes of the DE miRNAs were involved in starch, sucrose, fructose, and mannose metabolisms. For example, the novel miRNAs, mul-miRn23, mul-miRn30, and mul-miRn53 were predicted to target the probable fructokinase-1, glucan endo-1,3-beta-glucosidase-like gene, and galacturonosyltransferase 8 genes, respectively. In addition, some target genes of the DE miRNAs were involved in the metabolism of amino acid, lipid, fatty acid, and some secondary metabolites. Therefore, miRNAs might be master modulators of some metabolic process during fruit ripening.

The coloration of mulberry fruit during ripening is associated with the production of anthocyanin. In this study, some target genes of the DE miRNAs were involved in anthocyanin metabolism. It’s well documented that mul-miR156 can target the SQUAMOSA PROMOTER BINDING PROTEIN-LIKE (SPL) genes, which negatively regulates anthocyanin accumulation by depressing the expression of anthocyanin biosynthetic genes (Zhao et al., 2017; Chaves-Silva et al., 2018). Our data showed that the accumulation of mul-miR156e and mul-miR156t increased during fruit ripening (Table 1), and mul-miR156e was predicted to target the *SPL16* gene and mul-miR156t was predicted to target the *SPL4* and *SPL16* genes (Supplementary Table 2). In addition, the accumulation of mul-miR156e-3p decreased during fruit ripening, and the mul-miRNA was predicted to target the UDP-glycosyltransferase 75B1 gene, which was involved in the modification pathway of highly glycosylated anthocyanin (Wang et al., 2018). Therefore, the differential expression of mul-miR156e and mul-miR156t might promote the accumulation of anthocyanin, and this is consistent with the color change during fruit ripening.

The ATP-binding cassette (ABC) transporters are a class of major ATP-powered transporters, which are involved in a broad range of biological functions. Based on amino acid sequence, presence of transmembrane domains (TMDs), protein solubility, and function, plant ABC transporters were classified into eight subfamilies using the nomenclature: A, B, C, D, E, F, G, and I (Lopez-Ortiz et al., 2019). Although all nucleotide-binding domains (NBDs) of the ABC transporters share a common evolutionary origin and mechanism, the sequences of the coupled TMDs may be different and show important mechanistic differences. Therefore, the substrates captured by different ABC transporters may be diverse (Verrier et al., 2008). It was suggested that flavonoid movement may be mediated by ABCC proteins (Buer et al., 2007), and some members of ABCC transporters, such as maize MRP1, are involved in anthocyanin accumulation (Goodman et al., 2004). It has been demonstrated that the ABCB19 protein of *Arabidopsis thaliana* is involved in the auxin transport and plays roles in regulating the expression of *CHS* gene and required for normal accumulation of anthocyanin (Lin and Wang, 2005). Though Mul-ABCB19 is an ortholog of AtABCB19, there was no obvious phenotypic difference between overexpressing *Mul-ABCB19* and wild type *Arabidopsis thaliana* plants other than there was more anthocyanin accumulation in the overexpressing *Mul-ABCB19 Arabidopsis thaliana* plants (Figure 8). Moreover, it was found that the expression of *CHS* gene was higher (Supplementary Figure 3) in the overexpressing *Mul-ABCB19 Arabidopsis thaliana* plant than that in the wild type plants. Therefore, it appears that overexpression of *Mul-ABCB19* affects anthocyanin level via transcription regulation of *CHS* genes, which, in turn, is possibly regulated by altered auxin transport. Since some members of the ABC transporter subfamily B members have been reported to be involved in the transport of secondary metabolites and exogenous substrates (Verrier et al., 2008), the role of Mul-ABCB19 as a direct transporter of anthocyanin cannot be completely ruled out. Therefore, further investigations to confirm the function of *Mul-ABCB19* are required. The studies involving the development of knock-out lines of *Mul-ABCB19* and complementation experiments on *AtABCB19* mutant Arabidopsis, as well as *in vitro* transport assays using yeast or insect cells should provide detailed insights into the function of *Mul-ABCB19* in the future.

Interestingly, it was found that the antisense lncRNA, *Mul-ABCB19AS*, can suppress the expression of *Mul-ABCB19* and be targeted by mul-miR477. Therefore, *Mul-ABCB19AS* might modulate anthocyanin accumulation by suppressing the expression of *Mul-ABCB19*, whereas mul-miR477 acts as a positive regulator in anthocyanin accumulation by targeting the *Mul-ABCB19AS.* Based on these information obtained, it is speculated that there is a mul-miR477—*Mul-ABCB19AS*—*Mul-ABCB19* pathway, which may play an important role in regulating the accumulation of anthocyanin in mulberry, and the up-regulation of mul-miR477 during fruit ripening might promote mulberry fruit coloring.

## Conclusion

In conclusion, the dynamic regulation of miRNAs during mulberry fruit ripening was explored using high-throughput sequencing and molecular biology approaches. The DE miRNAs and their target genes during mulberry fruit ripening were identified, and their probable roles were discussed. Our results provided that mul-miR477 acts as a positive regulator for anthocyanin accumulation through the regulatory network of mul-miR477—*Mul-ABCB19AS*—*Mul-ABCB19*. The information provided in the current study is particularly useful for understanding the function and mechanisms of miRNAs during mulberry fruit development and ripening.

## Data Availability Statement

The datasets presented in this study can be found in online repositories. The names of the repository/repositories and accession number(s) can be found below: NCBI – PRJNA718973 (https://www.ncbi.nlm.nih.gov/sra/PRJNA718973).

## Author Contributions

XJ conceived and designed the experiments. XD and CL performed the experiments. YW and QD analyzed the data. YG wrote the manuscript. All the authors have read and approved the final manuscript.

## Conflict of Interest

The authors declare that the research was conducted in the absence of any commercial or financial relationships that could be construed as a potential conflict of interest.

## Publisher’s Note

All claims expressed in this article are solely those of the authors and do not necessarily represent those of their affiliated organizations, or those of the publisher, the editors and the reviewers. Any product that may be evaluated in this article, or claim that may be made by its manufacturer, is not guaranteed or endorsed by the publisher.

## Footnotes

1
http://plantgrn.noble.org/psRNATarget/

## Supplementary Material

Supplementary Figure 1 Alignment of the amino acid sequences of Mul-ABCB19 and AtMDR1. The amino acid sequences were aligned using DNAMAN software (version 6.0) and the identical amino acid residues were black shaded.

Supplementary Figure 2 Genome PCR detections of the *Mul-ABCB19AS* and *Mul-MIR477* genes in the transgenic and hybrid Arabidopsis plants. **(A,B)** Genome PCR detections of the *Mul-MIR477* **(A)** and *Mul-ABCB19AS* **(B)** genes in the transgenic Arabidopsis plants. **(C,D)** Genome PCR detections of *Mul-MIR477* **(C)** and *Mul-ABCB19AS* **(D)** genes in the hybrid Arabidopsis genome. WT represents wild type Arabidopsis, and OE1-3 represents different transgenic *Mul-MIR477* or *Mul-ABCB19AS* lines. H1-3 indicates different hybrid plants of transgenic *Mul-MIR477* and *Mul-ABCB19AS* plants.

Supplementary Figure 3 Expression analysis of chalcone synthase (*CHS*) gene in the transgenic *Mul-ABCB19* and the *Mul-ABCB19* × *Mul-ABCB19AS* hybrid plants. The *ACTIN* gene was amplified as reference gene. Three biological replicates per treatment were assayed and three technical replicates were performed for each sample. The values are expressed as the mean ± SD, Significant differences by Duncan’s multiple range test (*P* < 0.05) are indicated by different letters. OE-ABCB19 indicates the transgenic *Mul-ABCB19AS* and *Mul-ABCB19* plants, and AS × 19 indicates hybrid lines of transgenic *Mul-ABCB19AS* and *Mul-ABCB19* plants.

Supplementary Table 1 Novel identified miRNAs in mulberry fruits by sequencing.

Supplementary Table 2 Predicted target genes of the DE miRNAs and their Gene Ontology and KEGG analysis.

Supplementary Table 3 Forward primers used in miRNA qRT-PCR analysis.

Supplementary Table 4 Primers used for mRNA qRT-PCR analysis.
