# Supplementary figures and images for: MicroRNA Profiling During Mulberry (Morus atropurpurea Roxb) Fruit Development and Regulatory Pathway of miR477 for Anthocyanin Accumulation
Source: Front Plant Sci. 2021 Sep 8;12:687364. doi: 10.3389/fpls.2021.687364 (PMC8455890; doi:10.3389/fpls.2021.687364)

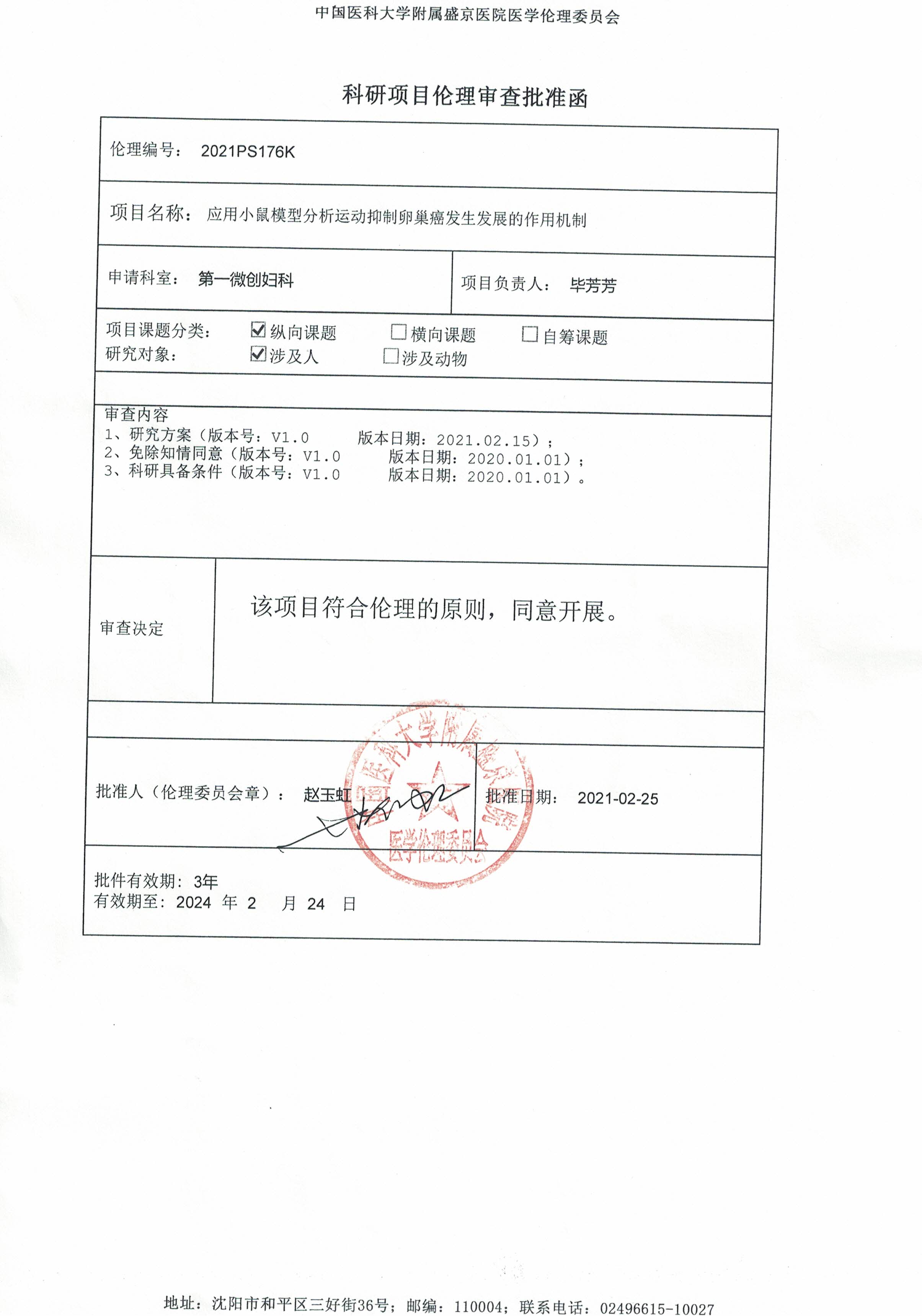

Supplement: Supplementary Figure 1 — Alignment of the amino acid sequences of Mul-ABCB19 and AtMDR1. The amino acid sequences were aligned using DNAMAN software (version 6.0) and the identical amino acid residues were black shaded. [file Image_1.JPEG]

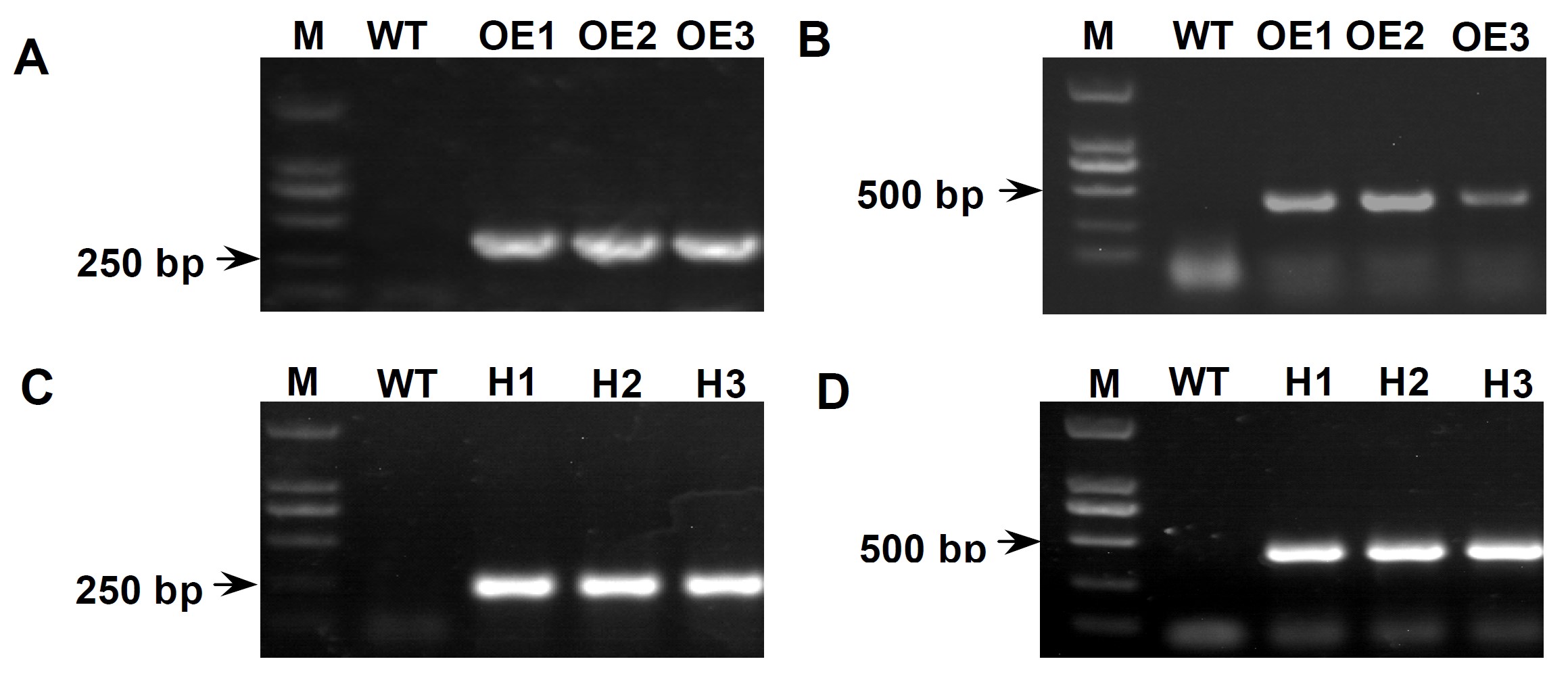

Supplement: Supplementary Figure 2 — Genome PCR detections of the Mul-ABCB19AS and Mul-MIR477 genes in the transgenic and hybrid Arabidopsis plants. (A,B) Genome PCR detections of the Mul-MIR477 (A) and Mul-ABCB19AS (B) genes in the transgenic Arabidopsis plants. (C,D) Genome PCR detections of Mul-MIR477 (C) and Mul-ABCB19AS (D) genes in the hybrid Arabidopsis genome. WT represents wild type Arabidopsis, and OE1-3 represents different transgenic Mul-MIR477 or Mul-ABCB19AS lines. H1-3 indicates different hybrid plants of transgenic Mul-MIR477 and Mul-ABCB19AS plants. [file Image_2.JPEG]

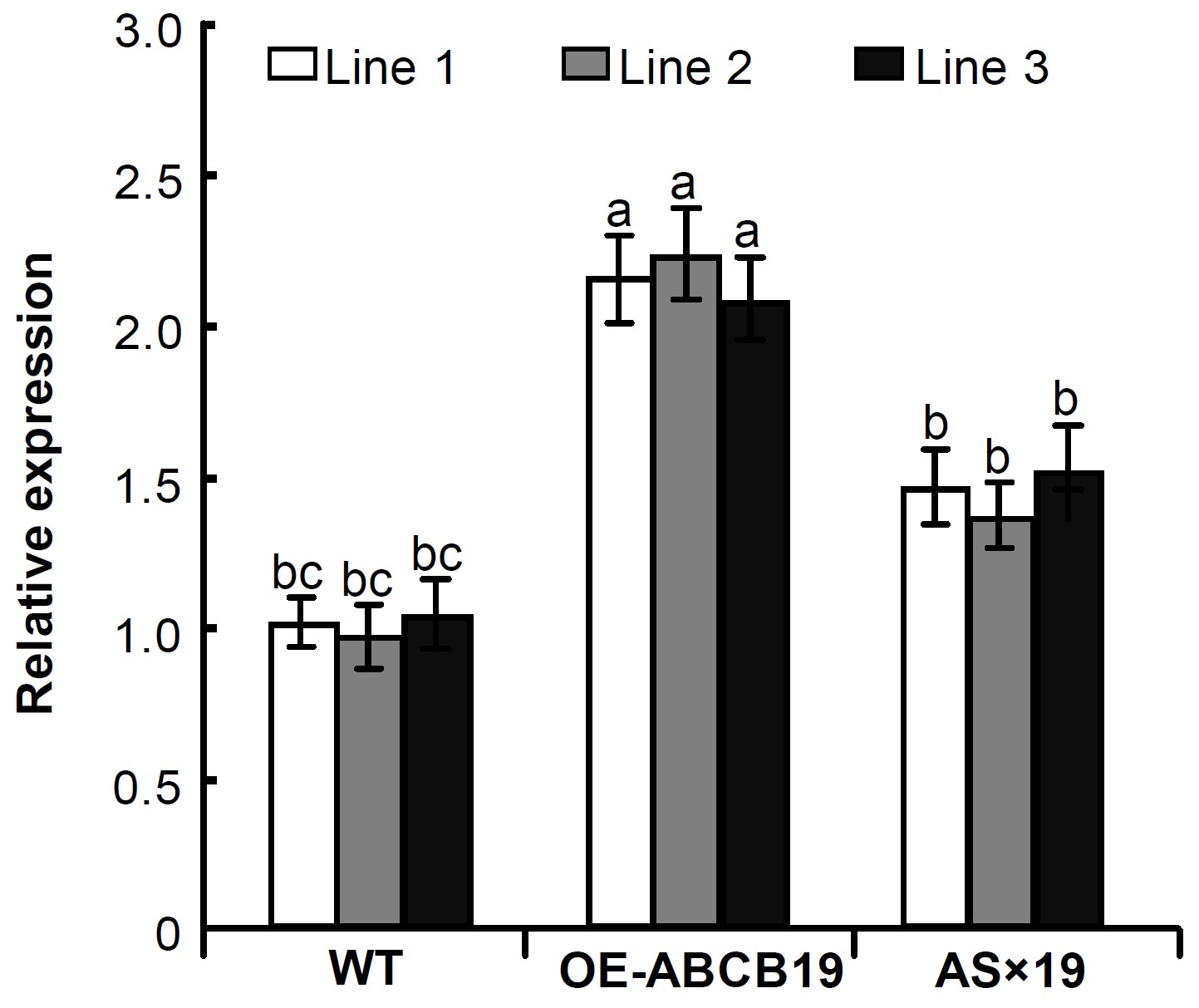

Supplement: Supplementary Figure 3 — Expression analysis of chalcone synthase (CHS) gene in the transgenic Mul-ABCB19 and the Mul-ABCB19 × Mul-ABCB19AS hybrid plants. The ACTIN gene was amplified as reference gene. Three biological replicates per treatment were assayed and three technical replicates were performed for each sample. The values are expressed as the mean ± SD, Significant differences by Duncan’s multiple range test (P < 0.05) are indicated by different letters. OE-ABCB19 indicates the transgenic Mul-ABCB19AS and Mul-ABCB19 plants, and AS × 19 indicates hybrid lines of transgenic Mul-ABCB19AS and Mul-ABCB19 plants. [file Image_3.JPEG]
