# Supplementary material for: MicroRNA Profiling During Mulberry (Morus atropurpurea Roxb) Fruit Development and Regulatory Pathway of miR477 for Anthocyanin Accumulation
Source: Front Plant Sci. 2021 Sep 8;12:687364. doi: 10.3389/fpls.2021.687364 (PMC8455890; doi:10.3389/fpls.2021.687364)
Supplement: Supplementary Table 1 — Novel identified miRNAs in mulberry fruits by sequencing. [file Table_1.DOC]

Supplementary Table 1. Novel identified miRNAs in mulberry fruits by Illumina sequencing

| **MiR_name** | **MiR_seq** | **Length** | **Pre-miRNA_seq** | **Type** | **Pre-miRNA length** | **CG**  **(%)** | **dG** | **MFEI** | **MG**  **(norm)** | **MR**  **(norm)** | **MP**  **(norm)** | **Expression level** |
| --- | --- | --- | --- | --- | --- | --- | --- | --- | --- | --- | --- | --- |
| mul-miR01 | CAATAACTCCAAATTCAAGATT | 22 | aacacaaaattcaagttcacatctcacaaatcatctaagtgaacacacaattcaagattcaaacaCAATTCCAATTAAACTCACTTTtttaactccaatcttgaatttggagttattgaaaagaacgcattcatagtattgactaaaataacatgcgagaaaaaagacttaaaagataacacttacacggaatctaaggttgcgttcttttCAATAACTCCAAATTCAAGATTggagttaaaaaaatgagtttaattggaattgtgtttgaaacttgaattgtgtgt | 3' | 245 | 29.60 | -133.60 | 1.60 | 51.85 | 43.61 | 30.34 | middle |
| mul-miR02 | CAATTCCAATTAAACTCATTTT | 22 | ctcaagttcacatctcacaaatcatccaagtgaacacacaattcaagtttcaaacaCAATTCCAATTAAACTCATTTTtttaactccaatcttgaatttggagttattgaaaagaacgcaaccttagattccgtgtaagtgttatcttttaagtcttttttctcgcatgttattttagtcaatactatgaatgcgttcttttcaataactccaaattcaagattggagttaaaaaagtgagtttaattggaattgtgTTTGAATCTTGAATTGTGTGT | 5' | 245 | 30.60 | -141.60 | 1.70 | 51.85 | 43.61 | 30.34 | middle |
| mul-miR03 | TTTGAATCTTGAATTGTGTGT | 21 | ctcaagttcacatctcacaaatcatccaagtgaacacacaattcaagtttcaaacaCAATTCCAATTAAACTCATTTTtttaactccaatcttgaatttggagttattgaaaagaacgcaaccttagattccgtgtaagtgttatcttttaagtcttttttctcgcatgttattttagtcaatactatgaatgcgttcttttcaataactccaaattcaagattggagttaaaaaagtgagtttaattggaattgtgTTTGAATCTTGAATTGTGTGT | 3' | 245 | 30.60 | -141.60 | 1.70 | 23.66 | 11.85 | 14.42 | middle |
| mul-miR04 | TGTAGGATCATGGCCCGCTCA | 21 | ggccaagatcctccgggctaggagtccatgtgctccaaacacgtctcagcctgtccaccgtgaaagtggaccgaactccttaagccatcggagtacacctgtttccagggccgcatctctgcggtattgggcccggggactttcctgggtcgaatccaatgtttTGTAGGATCATGGCCCGCTCAcctcaccacgagatgaagtgagcgggccatgatcctacaaaacgtaggattcggcccaggaaagtccccgggcccaataccgcagagatgcggccctggaaacaggtgtactccgatggcttaaggagttcggtccactttcacggTGGACAGGCTGAGACGTGTTTggagcacatggactcctagcccggaggatcttg | 5' | 382 | 57.40 | -409.80 | 1.90 | 63.43 | 81.05 | 72.61 | middle |
| mul-miR05 | TGGACAGGCTGAGACGTGTTT | 21 | ggccaagatcctccgggctaggagtccatgtgctccaaacacgtctcagcctgtccaccgtgaaagtggaccgaactccttaagccatcggagtacacctgtttccagggccgcatctctgcggtattgggcccggggactttcctgggtcgaatccaatgtttTGTAGGATCATGGCCCGCTCAcctcaccacgagatgaagtgagcgggccatgatcctacaaaacgtaggattcggcccaggaaagtccccgggcccaataccgcagagatgcggccctggaaacaggtgtactccgatggcttaaggagttcggtccactttcacggTGGACAGGCTGAGACGTGTTTggagcacatggactcctagcccggaggatcttg | 3' | 382 | 57.40 | -409.80 | 1.90 | 26.68 | 45.50 | 25.86 | middle |
| mul-miR06 | ATTGCGGAGCCGAATAACATTGCT | 24 | aatgtgaagtgtggtgcctctgtATTGCGGAGCCGAATAACATTGCTcttctaagtaattgagCAATGTTGCTTGGCTTCACAATATtggacacttcattctcacacg | 5' | 104 | 42.60 | -47.80 | 1.00 | 1171.86 | 1057.95 | 825.59 | middle |
| mul-miR07 | CAATGTTGCTTGGCTTCACAATAT | 24 | aatgtgaagtgtggtgcctctgtATTGCGGAGCCGAATAACATTGCTcttctaagtaattgagCAATGTTGCTTGGCTTCACAATATtggacacttcattctcacacg | 3' | 104 | 42.60 | -47.80 | 1.00 | 27.18 | 25.60 | 15.92 | middle |
| mul-miR08 | TTTGTAGTTGAATTTGAAGACA | 22 | tctcactcccatgtctttcTTTGTAGTTGAATTTGAAGACAtcacgatagcataactatcaactgatatcaaagccattcttgTCTTCAATTCGACTACAAAGGtggccatggaaagtgtgc | 5' | 116 | 39.30 | -45.10 | 0.90 | 2325.61 | 1323.38 | 845.49 | middle |
| mul-miR09 | TCTTCAATTCGACTACAAAGG | 21 | tctcactcccatgtctttcTTTGTAGTTGAATTTGAAGACAtcacgatagcataactatcaactgatatcaaagccattcttgTCTTCAATTCGACTACAAAGGtggccatggaaagtgtgc | 3' | 116 | 39.30 | -45.10 | 0.90 | 380.55 | 899.63 | 768.89 | middle |
| mul-miR10 | TTTCCTCTGGATCTAGGCAATT | 22 | tgctcagataataaaatagatgagttttggcataaaattggatctagtcaggtctaagtgaactgcctagatctgaaggaaagcgatgtccattttctattaggacacatattgactcggctcaagccagttcaaatcatcacctcatgtaaaacaaacacaccattatgtgtcctagtagaaaatggacatcatTTTCCTCTGGATCTAGGCAATTCACTtagatctgactatatctaattttatgccaaaactcatctcttttattacctgaccaacatctgtaatagaactcataaccccaaactaaagatctatacaacacatatcaattgtatcttgccatttacaacctccttcctttcagcctgattacaaaggatgatatctagtatttatggtgccaaacaaccttatatc | 5' | 277 | 35.70 | -176.10 | 1.20 | 18.12 | 16.12 | 11.94 | middle |
| mul-miR11 | TCTGGATCTAGGCAATTCACT | 21 | tgctcagataataaaatagatgagttttggcataaaattggatctagtcaggtctaagtgaactgcctagatctgaaggaaagcgatgtccattttctattaggacacatattgactcggctcaagccagttcaaatcatcacctcatgtaaaacaaacacaccattatgtgtcctagtagaaaatggacatcatTTTCCTCTGGATCTAGGCAATTCACTtagatctgactatatctaattttatgccaaaactcatctcttttattacctgaccaacatctgtaatagaactcataaccccaaactaaagatctatacaacacatatcaattgtatcttgccatttacaacctccttcctttcagcctgattacaaaggatgatatctagtatttatggtgccaaacaaccttatatc | 3' | 277 | 35.70 | -176.10 | 1.20 | 73.49 | 60.67 | 83.55 | middle |
| mul-miR12 | TCCATTTGAAATTTGGCATGTTCT | 24 | atTCCATTTGAAATTTGGCATGTTCTtatcataaatattaagaccatgttgaatTTTAAATGGTAACCTTACAACGaatgt | 5' | 60 | 27.20 | -26 | 1.20 | 9.06 | 68.25 | 25.86 | middle |
| mul-miR13 | TTTAAATGGTAATCTTACAACG | 22 | atTCCATTTGAAATTTGGCATGTTCTtatcataaatattaagaccatgttgaatTTTAAATGGTAACCTTACAACGaatgt | 3' | 60 | 27.20 | -26 | 1.20 | 10.07 | 10.43 | 3.98 | middle |
| mul-miR14 | AAACACCAAGCTTCGTAGAGAGAGA | 25 | gactcttccgcgcgcactccagtcaaagacaggagacaaacaaccataaaccagatcggaaccctagacgccatttttctctctctacgaagcttggtgttttggtgagtcctttctgcttgctcgctctggatcgtcgttttacgcttacacctccttaaaacccagacCCAGAGCGAGCAAGCAGAAAGgactcaccaAAACACCAAGCTTCGTAGAGAGAGAaaaatggcgtctagggttccgatctggtttatggttgtttgtctcctgtctttgactggagtgcgcgcggaagagtctgaatcgaaggagttcgttctcactttggaccactccaacttctccgacaccgtcggcaagcatgacttcgtcgtcgtcgagtt | 3' | 302 | 50.50 | -302.70 | 1.60 | 1.01 | 4.27 | 10.44 | middle |
| mul-miR15 | CGGGCCTGGGAGGTTTGGTAGG | 22 | tctctgagagaaagggaaagagagtttgagctttatgaagttgtCGGGCCTGGGAGGTTTGGTAGGagtaataagtaattaccatttagttttttgttcacttaattgatattataattgtatgttttaatttagttctccTTCCAAATCCACCCATGCCCACaatttcctcaggcttctctctttcccttattcatctcagtcaagtcatatat | 5' | 199 | 36.30 | -80.70 | 1.00 | 278.87 | 256.90 | 136.27 | middle |
| mul-miR16 | TTCCAAATCCACCCATGCCCAC | 22 | tctctgagagaaagggaaagagagtttgagctttatgaagttgtCGGGCCTGGGAGGTTTGGTAGGagtaataagtaattaccatttagttttttgttcacttaattgatattataattgtatgttttaatttagttctccTTCCAAATCCACCCATGCCCACaatttcctcaggcttctctctttcccttattcatctcagtcaagtcatatat | 3' | 199 | 36.30 | -80.70 | 1.00 | 6241.89 | 15957.34 | 19433.24 | high |
| mul-miR17 | CAGTCCTATGGCAACCTTGTC | 21 | cattaatgctgcgataaaactcatctcatgtcatatcctgagggtctctcacggtgttctcccaccagatgtgagcatcgtcgacgagCAGTCCTATGGCAACCTTGTCtaggagtcgacataatttaggaaactggtaacacttcacaagagctgattctagacttgtgatattgacatcgaatgctagacgaggttggccgtaggacaacttgtcAATGATGTCGCATCTGGTGGGagatcactgggagagaccctcaagatttgacatgagatgaattttatcgtaacattatcgctcgttttcct | 5' | 292 | 45.00 | -179.50 | 1.30 | 15.10 | 11.38 | 21.88 | middle |
| mul-miR18 | AATGATGTCGCATCTGGTGGG | 21 | cattaatgctgcgataaaactcatctcatgtcatatcctgagggtctctcacggtgttctcccaccagatgtgagcatcgtcgacgagCAGTCCTATGGCAACCTTGTCtaggagtcgacataatttaggaaactggtaacacttcacaagagctgattctagacttgtgatattgacatcgaatgctagacgaggttggccgtaggacaacttgtcAATGATGTCGCATCTGGTGGGagatcactgggagagaccctcaagatttgacatgagatgaattttatcgtaacattatcgctcgttttcct | 3' | 292 | 45.00 | -179.50 | 1.30 | 23.16 | 10.43 | 10.94 | middle |
| mul-miR19 | TTTGTGAGTGAATCTAAAGCA | 21 | tttgttttccatttatggcattaaagtttggtttccattgatgaggtcagagtaagaaggaggaggcatatttcaggTTTGTGAGTGAATCTAAAGCAacggcgaattccttgttttggggatttttcgtgggCATTAGATTCACCCACAAACTcgtaatcttccttttcatttttcttcttctttgaacttcccaattcaatttgatttgattgctcccggaaaatttg | 5' | 224 | 37.40 | -75.90 | 0.90 | 46.31 | 14.22 | 9.95 | middle |
| mul-miR20 | CATTAGATTCACCCACAAACT | 21 | tttgttttccatttatggcattaaagtttggtttccattgatgaggtcagagtaagaaggaggaggcatatttcaggTTTGTGAGTGAATCTAAAGCAacggcgaattccttgttttggggatttttcgtgggCATTAGATTCACCCACAAACTcgtaatcttccttttcatttttcttcttctttgaacttcccaattcaatttgatttgattgctcccggaaaatttg | 3' | 224 | 37.40 | -75.90 | 0.90 | 76.51 | 61.62 | 46.75 | middle |
| mul-miR21 | TTTTGTTCCGATGGAGATTTATCT | 24 | tctagacttcattttgaaaaaagaaaactctTTTTGTTCCGATGGAGATTTATCTtcatagatacaaaaaagtggactccacgcgataggattcatgtaaatccttcatgtgggacctatttgtgtgtctgatgGGGATAAATCTCCATCGGGACAAAtagtgttactttttaaaaaatgatagtttttc | 5' | 184 | 34.20 | -83.50 | 1.30 | 38.26 | 35.08 | 20.89 | middle |
| mul-miR22 | GGGATAAATCTCCATCGGGACAAA | 24 | tctagacttcattttgaaaaaagaaaactctTTTTGTTCCGATGGAGATTTATCTtcatagatacaaaaaagtggactccacgcgataggattcatgtaaatccttcatgtgggacctatttgtgtgtctgatgGGGATAAATCTCCATCGGGACAAAtagtgttactttttaaaaaatgatagtttttc | 3' | 184 | 34.20 | -83.50 | 1.30 | 244.64 | 164.00 | 96.48 | middle |
| mul-miR23 | TAATGCGGGGATGGCGTGCTT | 21 | actgctaacgtggtcgttagttcgggggaggggcaacagacgcctcatcctccTAATGCGGGGATGACGTGCTTacaagcagacgcgaggacttcagatgggtatggagctggtaattcacagtacgcagcatatcaatgacttcttgtgattagtgtgaattatcaactccatactcatctgaagccccctcgttcgtcTGTAAGCACGCCATCCCCGCActaggaggaagaggcgcccgtcgcccatctcccgaactagcgaccacgttagc | 5' | 271 | 54.00 | -189.10 | 1.30 | 180.21 | 381.09 | 458.55 | middle |
| mul-miR24 | TGTAAGCACGTCATCCCCGCA | 21 | actgctaacgtggtcgttagttcgggggaggggcaacagacgcctcatcctccTAATGCGGGGATGACGTGCTTacaagcagacgcgaggacttcagatgggtatggagctggtaattcacagtacgcagcatatcaatgacttcttgtgattagtgtgaattatcaactccatactcatctgaagccccctcgttcgtcTGTAAGCACGCCATCCCCGCActaggaggaagaggcgcccgtcgcccatctcccgaactagcgaccacgttagc | 3' | 271 | 54.00 | -189.10 | 1.30 | 35.24 | 72.99 | 78.58 | middle |
| mul-miR25 | TGAGAATAGCTGACTGAACTGT | 22 | tagctggctgaactgtaatcattatatagagaggggggagagagagagataatatggcTGAGAATAGCTGACTGAACTGTaatcactatcacatatatATAGGGTAGCGATTGCAGTTCAGTcaattgttctcagctagattatgtgatcatgattcacc | 5' | 96 | 39.40 | -58.50 | 0.90 | 9.06 | 14.22 | 0.00 | middle |
| mul-miR26 | ATAGGGTAGCGATTGCAGTTCAGT | 24 | tagctggctgaactgtaatcattatatagagaggggggagagagagagataatatggcTGAGAATAGCTGACTGAACTGTaatcactatcacatatatATAGGGTAGCGATTGCAGTTCAGTcaattgttctcagctagattatgtgatcatgattcacc | 3' | 96 | 39.40 | -58.50 | 0.90 | 12.08 | 4.74 | 1.99 | middle |
| mul-miR27 | AATGGGCTGTTTGGGAAGAAAT | 22 | aaatatcaaagaattattcgcttttgattagttgaataagaagaaaaatgagaagagggaaagggagatttgagctactggaaattgtaggAATGGGCTGTTTGGGAAGAAAGaaaataagggaaaaagaaaaaaaaaatagatcataagtttcttgtcaatctatattcttccTTCCCAAGGCCGCCCATTCCGAtgatttctagaaggtcatccctttcccttccatattctctctacctccatgataatactgttt | 5' | 186 | 35.10 | -88.40 | 1.00 | 73.49 | 110.91 | 177.05 | middle |
| mul-miR28 | TTCCCAAGGCCGCCCATTCCGA | 22 | aaatatcaaagaattattcgcttttgattagttgaataagaagaaaaatgagaagagggaaagggagatttgagctactggaaattgtaggAATGGGCTGTTTGGGAAGAAAGaaaataagggaaaaagaaaaaaaaaatagatcataagtttcttgtcaatctatattcttccTTCCCAAGGCCGCCCATTCCGAtgatttctagaaggtcatccctttcccttccatattctctctacctccatgataatactgttt | 3' | 186 | 35.10 | -88.40 | 1.00 | 7461.07 | 7908.05 | 11736.33 | high |
| mul-miR29 | TGTTGGACGAAATTTTGCACC | 21 | atgctggTGCAAAATTTCGTCCAACACCaaaagaacgtccttggactccgataattatttcttatttgtttttgcatagttcatggcgttcatctagccgatcacactatgtttttggttaaagtgtttatttgatccggattacacgttcaagaaaacgttattctggTGTTGGACGAAATTTTGCACCaaca | 3' | 193 | 37.60 | -73.50 | 1.00 | 23.66 | 16.59 | 28.85 | middle |
| mul-miR30 | TTTTTTATTCAGATTGCAATC | 21 | acacttgacaagcaatttatttagctgacgagggtttacaatgtgacagactctgttttagtaatctatgatcgcttttttgcgactcttgcaatgattaatcatcaaagatttaaaaatcaattagttttcgtgcgtaagtgatcatatttcttttttttattgttatttttttaatattgaatttgTTTTTTATTCAGATTGCAATCgatgtaaataaatcacctcatcaatgttaagttttaTATTTCGTTCCTGGACATATTactctttttaaggggatgattgccttgacggagggagagtgctctcatatatcttaaaagagcaatatgtccaggaacaaaatatgaagcttgacgttgaggaggtggtttattttcatcgatagcaatctgaataaaaaata | 5' | 223 | 31.10 | -154.30 | 1.20 | 602.54 | 703.87 | 1824.76 | middle |
| mul-miR31 | TATTTCGTTCCTGGACATATT | 21 | acacttgacaagcaatttatttagctgacgagggtttacaatgtgacagactctgttttagtaatctatgatcgcttttttgcgactcttgcaatgattaatcatcaaagatttaaaaatcaattagttttcgtgcgtaagtgatcatatttcttttttttattgttatttttttaatattgaatttgTTTTTTATTCAGATTGCAATCgatgtaaataaatcacctcatcaatgttaagttttaTATTTCGTTCCTGGACATATTactctttttaaggggatgattgccttgacggagggagagtgctctcatatatcttaaaagagcaatatgtccaggaacaaaatatgaagcttgacgttgaggaggtggtttattttcatcgatagcaatctgaataaaaaata | 3' | 223 | 31.10 | -154.30 | 1.20 | 240.11 | 236.05 | 451.09 | middle |
| mul-miR32 | CTAAAGGATGCAGAGGTGTGA | 21 | agacaaccgtgtgaccatcccagccagctaagctaaccgggaacgatcctctacagctgagatgatcttcgcagaccatcaagccgttgctcacgctctgatcacacggtcatgattcatccaccaggttgaggtcctgtctccttcgaaCTAAAGGATGCAGAGGTGTGActttacttgaacaggaTCTGTTCTGTAGATTCACACCtctgcatcctttagttcgaaggagacaggacctcaacctggtggatgaatcatgaccgtgtgatcagagcgtgagcaacggcttgatggtctgcgaagatcaactcagctgtagaggatcgttctcagctatttcctggctggtgtgcggtcacacggttgtctgacaggttagtcttttttctc | 5' | 372 | 50.40 | -340.40 | 1.70 | 65.44 | 78.68 | 33.82 | middle |
| mul-miR33 | TCTGTTCTGTAGATTCACACC | 21 | agacaaccgtgtgaccatcccagccagctaagctaaccgggaacgatcctctacagctgagatgatcttcgcagaccatcaagccgttgctcacgctctgatcacacggtcatgattcatccaccaggttgaggtcctgtctccttcgaaCTAAAGGATGCAGAGGTGTGActttacttgaacaggaTCTGTTCTGTAGATTCACACCtctgcatcctttagttcgaaggagacaggacctcaacctggtggatgaatcatgaccgtgtgatcagagcgtgagcaacggcttgatggtctgcgaagatcaactcagctgtagaggatcgttctcagctatttcctggctggtgtgcggtcacacggttgtctgacaggttagtcttttttctc | 3' | 372 | 50.40 | -340.40 | 1.70 | 1611.82 | 1587.87 | 1177.71 | middle |
| mul-miR34 | CTTCTACCCTATCACATCTTTC | 22 | aaagaaacaactaagcagcaaaacactacattttgacccTTTCTACCCTATCACATCTTTCcaattcatagaagtggaagacatttacaagaacgcaaactttgatgccacattgcgggcatcttcagataaaatttttcagaattatcatttctcaattggAAAGATGTGATAGGGTAGAAGGGGtcaaaatgttgtgttttattacctagcggtttccca | 5' | 216 | 36.50 | -98.10 | 1.20 | 5.03 | 11.85 | 29.84 | middle |
| mul-miR35 | AAAGTGATCGTGTCGTGTTCGTGT | 24 | gtAAAGTGATCGTGTCGTGTTCGTATtgtgatttttaaaaaattgtgtcacgagtcgtgttcgaattgactataaatttaaaaagaaacgatcaaaacacaatacgAACACGATACATGACACGAATTGTtatcccta | 5' | 118 | 33.30 | -40.60 | 0.90 | 5.03 | 11.85 | 29.84 | middle |
| mul-miR36 | AACACGACACATGACACGAATTGT | 24 | gtAAAGTGATCGTGTCGTGTTCGTATtgtgatttttaaaaaattgtgtcacgagtcgtgttcgaattgactataaatttaaaaagaaacgatcaaaacacaatacgAACACGATACATGACACGAATTGTtatcccta | 3' | 118 | 33.30 | -40.60 | 0.90 | 19.13 | 11.38 | 18.90 | middle |
| mul-miR37 | AATGAGGTTTGATCCGAAATC | 21 | gagaggaaaacaggtatgtggAATGAGGTTTGATCCGAAATCcctttgtcttcttctctaatagatgacattggctgtgatgttggaagaaattcagatggatttttcttggtttctattttccatgtttttgtttttcttCTTGTTTCAACATCAACGCACTgatctcggaccaggcttcattcctcacaccttgcttttcctaat | 5' | 201 | 38.60 | -71.10 | 0.90 | 16.11 | 5.69 | 0.99 | middle |
| mul-miR38 | CTTGTTTCAACATCAACGCACT | 22 | gagaggaaaacaggtatgtggAATGAGGTTTGATCCGAAATCcctttgtcttcttctctaatagatgacattggctgtgatgttggaagaaattcagatggatttttcttggtttctattttccatgtttttgtttttcttCTTGTTTCAACATCAACGCACTgatctcggaccaggcttcattcctcacaccttgcttttcctaat | 3' | 201 | 38.60 | -71.10 | 0.90 | 158.06 | 94.80 | 41.78 | middle |
| mul-miR39 | ATGAGTATTCCAGAAACAGTAGC | 23 | taatatatccacttgtaatgtttcttgctcaaATGAGTATTCCAGAAACAGTAGCattagtctatccgcgtgtatcaattaatTTGCTGCTGGTTTCTGAGATGctcatttgagcaagcaatgttacaagctggagat | 5' | 128 | 37.00 | -64.10 | 1.30 | 13.09 | 7.58 | 2.98 | middle |
| mul-miR40 | TAACACCACAAGAAATAGAAT | 21 | tatgtcatgattagtgatTAACACCACAAGAAATAGAATgtgggtccaacttatgaacgaatAAGTGAGAGCCACATCCTATTTGTtgtggcgttggtcactactagtgaccaa | 5' | 108 | 39.50 | -54.50 | 1.20 | 14.09 | 22.75 | 21.88 | middle |
| mul-miR41 | AAGTGAGAGCCACATCCTATTTGT | 24 | tatgtcatgattagtgatTAACACCACAAGAAATAGAATgtgggtccaacttatgaacgaatAAGTGAGAGCCACATCCTATTTGTtgtggcgttggtcactactagtgaccaa | 3' | 108 | 39.50 | -54.50 | 1.20 | 11.07 | 11.38 | 11.94 | middle |
| mul-miR42 | CGAACTTTCCTCTTCCCGACC | 21 | aactccttcaccaacaacattcaccttttccccaccgttattgaccaaaaaagttcCGAACTTTCCTCTTCCCGACCactttccgcaacttcttttgccttttctggaaATTGGTCGGGATGATGGAAGTTCGgaaccttcagtcaagaatggtggggaaaagatgaatgttgttgtggaggatgt | 5' | 185 | 44.60 | -106.40 | 1.30 | 66.45 | 152.62 | 180.04 | middle |
| mul-miR43 | ATTGGTCGGGATGATGGAAGTTCG | 24 | aactccttcaccaacaacattcaccttttccccaccgttattgaccaaaaaagttcCGAACTTTCCTCTTCCCGACCactttccgcaacttcttttgccttttctggaaATTGGTCGGGATGATGGAAGTTCGgaaccttcagtcaagaatggtggggaaaagatgaatgttgttgtggaggatgt | 3' | 185 | 44.60 | -106.40 | 1.30 | 21.14 | 52.14 | 36.80 | middle |
| mul-miR44 | TCATCCTCATAAATTCAGTCTC | 22 | acaacttgtcacggtgtcatatgattgcatcttaaatctatcaactttatgtatatacatttTCATCCTCATAAATTCAGTCTCaccagtctgtgattgggcctgtatcacaagctagagccacccactcaccttcatctatatcacaggctatgtgagactcaatttatgaagggtgaaaaggtatatatataaggtTGATAGATTTAGGATGCGATCatatgacaccgtgacaagctatgccgaat | 5' | 233 | 38.30 | -141.10 | 1.50 | 71.48 | 86.27 | 54.71 | middle |
| mul-miR45 | TGATAGATTTAGGATGCGATC | 21 | acaacttgtcacggtgtcatatgattgcatcttaaatctatcaactttatgtatatacatttTCATCCTCATAAATTCAGTCTCaccagtctgtgattgggcctgtatcacaagctagagccacccactcaccttcatctatatcacaggctatgtgagactcaatttatgaagggtgaaaaggtatatatataaggtTGATAGATTTAGGATGCGATCatatgacaccgtgacaagctatgccgaat | 3' | 233 | 38.30 | -141.10 | 1.50 | 158.06 | 157.36 | 107.43 | middle |
| mul-miR46 | CACAGCGTTTTGCACGTGCCC | 21 | ttgttgccgctgctcgtgcagattccctccaacgaCACAGCGTTTTGCACGTGCCCgttttgcttgctcacaacagtctccatttgtgtgggaaatgctttaagagttgtgtgtgtgtatataatatggagatgttggagctagagagagagagagagagtttagagagaggaaatccttactacccataaagacctttgATTTCCTCTCTCTAAACTCTCTctctctctctctagctccaacatctccatattatatacacacacacaactcttaaagcatttcccacacaaatggagactgttgtgagcaagcaaaacgggcacgtgcaaaacgctgtgtcgttggagggaatctgcacgagcagcggcaacaatgg | 5' | 376 | 45.90 | -357.90 | 2.10 | 5.03 | 10.90 | 3.48 | middle |
| mul-miR47 | ATTTCCTCTCTCTAAACTCTCT | 22 | ttgttgccgctgctcgtgcagattccctccaacgaCACAGCGTTTTGCACGTGCCCgttttgcttgctcacaacagtctccatttgtgtgggaaatgctttaagagttgtgtgtgtgtatataatatggagatgttggagctagagagagagagagagagtttagagagaggaaatccttactacccataaagacctttgATTTCCTCTCTCTAAACTCTCTctctctctctctagctccaacatctccatattatatacacacacacaactcttaaagcatttcccacacaaatggagactgttgtgagcaagcaaaacgggcacgtgcaaaacgctgtgtcgttggagggaatctgcacgagcagcggcaacaatgg | 3' | 376 | 45.90 | -357.90 | 2.10 | 7.55 | 12.80 | 24.37 | middle |
| mul-miR48 | CACCAACTTACCTGGCATAGAGT | 23 | tagtgctagggacgccaactcttctcgactattttgCACCAACTTACCTGGCATAGAGTtatcatcttttcattggtaggtcttcccaactattttgtaccAATAAAAAGATGACAACTCACATGccacgtaagtttgtacaaaatagttaggattagagttggtgtccctagcaaaa | 5' | 172 | 39.30 | -93.30 | 1.30 | 14.09 | 20.86 | 19.89 | middle |
| mul-miR49 | AATAAAAGGATGACAACTCACATG | 24 | tagtgctagggacgccaactcttctcgactattttgCACCAACTTACCTGGCATAGAGTtatcatcttttcattggtaggtcttcccaactattttgtaccAATAAAAAGATGACAACTCACATGccacgtaagtttgtacaaaatagttaggattagagttggtgtccctagcaaaa | 3' | 172 | 39.30 | -93.30 | 1.30 | 10.07 | 8.53 | 10.94 | middle |
| mul-miR50 | ATGTGTATGTTCCTTAATTGG | 21 | acaatgaagtttggttacagaatctccacccccatgtccagctttaaggacaatttgacagagaggtttaaaaatccatcaagaggATGTGTATATTCCTTAATTGGcaagcaagagacgaattcagcaaacttcattgccctgtcaaactttattgtgtggacatttaaaagggcgtcggatgtacttgctgaattcgtctcctgcttaccaattaaggaacatacacatcctcttaatggatttttaaacctctttgtcaaatagcccttaaagccggacaTGGGCGTGGAGATTCTGTAACCaa | 5' | 297 | 40.40 | -195.90 | 1.60 | 16.61 | 23.70 | 27.35 | middle |
| mul-miR51 | TGGGGGTGGAGATTCTGTAACC | 22 | acaatgaagtttggttacagaatctccacccccatgtccagctttaaggacaatttgacagagaggtttaaaaatccatcaagaggATGTGTATATTCCTTAATTGGcaagcaagagacgaattcagcaaacttcattgccctgtcaaactttattgtgtggacatttaaaagggcgtcggatgtacttgctgaattcgtctcctgcttaccaattaaggaacatacacatcctcttaatggatttttaaacctctttgtcaaatagcccttaaagccggacaTGGGCGTGGAGATTCTGTAACCaa | 3' | 297 | 40.40 | -195.90 | 1.60 | 44.30 | 39.82 | 36.80 | middle |
| mul-miR52 | TGACACAAGATCATCCTCCAA | 21 | acaTGACACAAGATCATCCTTCAAatagaaatagaaacaaatcaacactcaacaatcaaccgccatgttgtaactctataaacacccacaaaaccgcaaaacccctagacttccccgttatcttgatattatccctctaacttctaggcgtaagccaataagaataatcaaggtaaaccaatgatttgtttctgtttttaTTTGGAGGATGATCTTGTGTCataaagtgaaatgttaggtagttgaagtcattcagctatttaatgatttattatgtatttaaataccattcaaacgtt | 5' | 221 | 33.40 | -94.70 | 0.90 | 0.50 | 30.81 | 40.28 | middle |
| mul-miR53 | TTTGGAGGATGATCTTGTGTC | 21 | acaTGACACAAGATCATCCTTCAAatagaaatagaaacaaatcaacactcaacaatcaaccgccatgttgtaactctataaacacccacaaaaccgcaaaacccctagacttccccgttatcttgatattatccctctaacttctaggcgtaagccaataagaataatcaaggtaaaccaatgatttgtttctgtttttaTTTGGAGGATGATCTTGTGTCataaagtgaaatgttaggtagttgaagtcattcagctatttaatgatttattatgtatttaaataccattcaaacgtt | 3' | 221 | 33.40 | -94.70 | 0.90 | 5.03 | 59.25 | 80.57 | middle |
| mul-miR54 | TAGGATCCTGGTTGAGTCCCA | 21 | gtgtgtTAGGATCCTAGTTGAGTCCCAggactcgAGACTCAACCAGGATCCTAACAGAg | 5' | 53 | 50.80 | -31.10 | 1.00 | 11.07 | 133.67 | 29.84 | middle |
| mul-miR55 | CGACCTTTGCGTTGCCGCTTC | 21 | cttaattgccgggatcggcaatgtgagggtctgataCGACCTTTGCGTTGCCGCTTCaggtaattcgaccctgggggcaaga | 3' | 62 | 54.90 | -38.60 | 0.90 | 0.00 | 96.69 | 94.50 | middle |
| mul-miR56 | AAACAGTGAATGTAGAGAGTGACA | 24 | ataactttttctcactctctttgcacTTTCTGCATTCACTGTTTGACATatgggtttcactaacataatatattattaaaatgagatactagagtccacgtgtcAAATAGTGAATGTAGAGAGTGACAgaaaaagtgatgttagaattt | 3' | 127 | 31.50 | -53.50 | 1.10 | 22.15 | 3.79 | 5.97 | middle |
| mul-miR57 | TAAATTGAGTCTCACATAGCC | 21 | aaccattcggcatagcttgtcacggtgtcatatgatcgcatcctaaatctatcaaccttatatatataccttttcacccttcaTAAATTGAGTCTCACATAGCCtgtgatatagatgaaggtgagtgggtggctctagcttgtgatacaggcccaatcacagactggtgagactgaatttatgaggatgaaaatgtatatacataaagttgatagatTTAAGATGCAATCATATGACAccgtgacaagttgtattaaaggatcaaaatacttgttttatattttagagatggttaattagagg | 5' | 241 | 36.00 | -137.10 | 1.30 | 25.17 | 19.91 | 12.93 | middle |
| mul-miR58 | TTCGCCCCATTCATGATTAGA | 21 | aaatgttctgactcatgctgctgaagtgacatttacacctgaatatacacatcctcttaatgttctgactcatagtcaaatACATAGTCTGATCATGAATTgggtgaataacgtgttgtactagtcatactgctaaggttgTTCGCCCCATTCATGATTAGAccatgttttttgaccatggagcagaaaag | 3' | 126 | 38.20 | -62.60 | 0.90 | 47.32 | 64.46 | 124.34 | middle |
| mul-miR59 | AGTGTTACAGGGTGCTCTCAAATC | 24 | ttttggtaaattaccccctgaatttaataaAGTGTTACAGGGTGCTCTCAAATCaatgcagactaaaaatttagacgaaaacttttgtctacatgttacgtgacctcgtttgaaggaagagactattattctgcatgatgattggagcatgtcatatcacatgcatgaggacattttctttcaaattttaacatcattcattgatttggggggtactctgcAACATTTTATTATATTCTGGGAGTaatttgcca | 5' | 251 | 34.60 | -94.80 | 1.10 | 8.05 | 14.22 | 17.90 | middle |
| mul-miR60 | AAAATCATGATTTGAGTTAGTTTA | 24 | aaattatggtgattctaaattttagaatcagaatcataatttatgattttaagtttaaattatgatTCAGAATCGGAGATTTGAATCACCttaaattaggaggggggttgttttAAAATCATGATTTGAGTTAGTTAAaacatcacatcaaattctgaatcatcattcataatcactgtaaataaactaagatctagaattataattctaaatctatttactaaacgc | 3' | 91 | 25.00 | -52.40 | 0.90 | 27.18 | 19.91 | 10.94 | middle |
| mul-miR61 | TCTTTACTCTTCTGTCAACACA | 22 | cttcctttttgttgacagaagataGAGAGCACAACTGAGCATATACCcagggaattgttcgtgtatgagcagttttgtgcTCTTTACTCTTCTGTCAACACAatcttctgcc | 3' | 91 | 42.00 | -49.30 | 1.00 | 9.06 | 15.17 | 26.86 | middle |
| mul-miR62 | CAGGGAACGGACAGAGCATGG | 21 | ataaggCAGGGAACGGACAGAGCATGGatggagccttcaacagaagaaggaatgctgttgtggctctactcatgcactgcctcttccctggctgtg | 5' | 90 | 53.10 | -49.70 | 1.00 | 66.45 | 65.41 | 37.80 | middle |
| mul-miR63 | TAGTACACCTGGCGTATCGAT | 21 | cgatacgccagacgtacaagagaatcttttaaaaaattaataaaatgagaaaaaagggagattctcTAGTACACCTGGCGTATCGATaggtcaggtttttctaaccgttagatatgcgtacttttatgtgattttatgcaaatccgacagataaaaaacttagcctaccggtATGTTGGCGTACCGGAGAAGGTCTcaaaaaaattctcttatcattt | 5' | 85 | 36.20 | -70.80 | 0.90 | 10.07 | 10.43 | 10.94 | middle |
| mul-miR64 | CGAGACACGAGATTTGCACATG | 22 | taattttggcaaaatgcacgctgagctctaaaatcacttgctattgaagtccaagagctgatgttttttgtcaaattgatagttCGAGACACGAGATTTGCACATGacatggtttgaaatgtgacgagctttccctttcaTGTGCAATTCTCGTGTCTTAGAccataaatttgataacgttcattagcaattggacctcaatcgctactaattttaaagcatatggtgcattttgtcaaaagtg | 5' | 238 | 36.90 | -104.80 | 1.20 | 12.08 | 8.53 | 11.44 | middle |
| mul-miR65 | TTAAGAGGATGTGTATGTTCC | 21 | acaatgaagtttggttacagaatctccacgcccatgtccggctttaagggctatttgacaaagaggtttaaaaatccaTTAAGAGGATGTGTATGTTCCttaattggtaagcaggagacgaattcagcaagtacatccgacgcccttttaaatgtccacacaataaagtttgacagggcaatgaagtttgctgaattcgtctcttgcttgccaattaaggaatatacacatcctcttgatggatttttaaacctctctgtcaaattgtccttaaagctggacatgggggtggagattct | 5' | 281 | 40.50 | -203.20 | 1.70 | 323.67 | 315.68 | 260.11 | middle |
| mul-miR66 | AGGAGGTGAGCCAACAATAGTTTT | 24 | gacagttctgttaaagtataaaactactgttggtctatcttctaacgacttaaacttttaactatAGGAGGTGAGCTAACAATAGTTTTataatttaacaagttcttaa | 3' | 103 | 28.40 | -32.70 | 1.10 | 9.06 | 14.22 | 9.95 | middle |
| mul-miR67 | AGAGAATTCTGTGGTAGGGTA | 21 | gatgAGAGAATTCTGTGGTAGGGTAcgaaatattactctatatgttggacacaatctccaactgtcaaatcatgtcagaatcattatcgggttaagaaaatatggcataaccaaaattaacaagcatttgacgattaaagattgtgccttgctagtaggttattttttcataccctaccatagaattctcccaca | 5' | 191 | 35.40 | -66.20 | 1.00 | 4.03 | 12.32 | 4.97 | middle |
| mul-miR68 | AGCATCCTCGTGTGGCATATGGAT | 24 | atttgaaacggttatagggcacctagataaatcctaggaggcacAGCATCCTCGTGTGGCATATGGATgagtttggaccgcggtggggatgctgtgcatcatacatattatctatatgccACACTGGAATGCTGTGTCTCCcggaggttatctcggtctcctact | 5' | 149 | 48.50 | -80.10 | 1.00 | 17.11 | 40.76 | 25.86 | middle |
| mul-miR69 | TAAGATGTTTTCACCCAATTGC | 22 | gattcctccaagccgagtgaaggaggccaaggaaagactgaaaaagaaattcaatgctgaccttgctctttgcacaaacaccgatctcggatgggatccctaaaaaattgcccaacatgcacggacaaatattgggatgacttctgcaaggttagtacttttttttatgtacttctgcatatgtagttgcaTAAGATGTTTTCACCCAATTGCTAATTtccaagttgcgttgtaaacagacgcacaaatattgtagttgcatatgtagttattttgcccatttgtgtgtctgtttacaatgcaacttcaaaatcagcaattgggtgaaaacatcttatgcaactacatgtgcagaagtacataaaaaaatgtactaaccttgcagaagtcatcccaatattcgtccgtgcatg | 5' | 298 | 39.00 | -244.40 | 1.50 | 161.08 | 141.25 | 146.22 | middle |
| mul-miR70 | TGTTAGGATCCTGGTTGAGTC | 21 | actcTGTTAGGATCCTGGTTGAGTCtcgagtcctgggactcaacTAGGATCCTAACACACTCTTACTT | 5' | 53 | 47.10 | -40.70 | 1.30 | 49.33 | 699.61 | 299.40 | middle |
| mul-miR71 | AATTTTTTGATACCAACTCACATG | 24 | gcccatcttgtacgaaagaatagtggtggggatcccAATTTTTTGATACCAACTCACATGgtaatgagttgtcatctctccattggtagatgcaattttgtAATATTAACTTGAAAGGCCTAccaataaaagaatggtcgctcatataccatgtgaattgatattaaaaagtttgggatttctagcattattccgtacgaaatgaca | 5' | 201 | 36.20 | -84.80 | 1.10 | 17.11 | 18.96 | 5.97 | middle |
| mul-miR72 | TCGTCCCAAATATGAGCATCC | 21 | ggctttaagttgtagattaggatagagtttggaccgatacagtacgacgtagctcacgttccagaggagcataaggATGCTCATATTTGGGACGATATGCaaattaaacgttatgaggctcttgaggcttattaatgcagactagatgttgctatatagtttcaagagcctcataatatctaatctgcataTCATCCCAAATATGAGCATCCatatgctcctctggaacatgagctacgccgtactgtatcgatccaaactctattctattctgcatcttaaataa | 3' | 280 | 39.50 | -189.20 | 1.70 | 12.08 | 21.80 | 23.87 | middle |
| mul-miR73 | AAATTGACAGTTTGAGACATG | 21 | acagtcttccaattgctaacgagcattatTAATTGACAGTTTGAGACATGaaattcacacacgacgtagttcgaaaaatggcaagctttgcatttcatgtgttaatctcgTGTCTCAAACTGTCAATTTATcattctttagctattggaccttaattgctacc | 5' | 142 | 36.20 | -55.40 | 0.90 | 13.59 | 2.37 | 10.44 | middle |
| mul-miR74 | ATGACAAATTACTGCGTTGAGTGA | 24 | gaaaagttgatggctaacgtatcactcaacgcaatactttgtcatacacttaacctgtatacgctcctcgacattccaaacaactaatgttaaagtgtATGACAAATTACTGCGTTGAGTGAtacattagccatcaacttgtatggaaattttcaaacttcagagttcagatattgtattaatagtaacaagtattattgcgtgttttttaatttttatgtttcctatatgtcgaaggaatttggttttggttgagtttgcatgtgaaggaaatggaaTCTTGTAGTTTTCGCACG | 5' | 137 | 33.80 | -94.50 | 0.90 | 23.16 | 18.96 | 10.94 | middle |
| mul-miR75 | TTTTCTCTTCTCTTAAGACTTC | 22 | atagaaatggtTTTTCTCTTCTCTTAAGACTTCtacaaatttcgctccccgcaatttacttgatcatctgacggataaagatatttatagaagtgttaagagaagaaaaaaccatttcataaatttcgtaacacttctataaattttatatacataaataaagatgatttttcttTTCTTTTAACACTTCTATAAA | 5' | 115 | 26.00 | -48.10 | 0.90 | 6.04 | 15.17 | 9.95 | middle |
| mul-miR76 | GGCGTGACCCCTGAGAACACAAG | 23 | acaccccagaGGCGTGACCCCTGAGAACACAAGgcaggtttgccaattgccattgttgctacatatttgttgaatTAAGCCGGTATAAATATATGCatgggccatggctaatgtgccttgtgttctcaggtcacccctttggggcat | 5' | 141 | 48.30 | -72.90 | 1.00 | 142.96 | 54.98 | 18.90 | middle |
| mul-miR77 | TTTCTCTATCGCTCTCCTCGTA | 22 | aacagcaacaagctCAACATCGTTCCTCAGAAGAGCaaccctagccaccaaacgcccgatccttccaaatccgttgattccgatcttgatcttcttgtctgaagccattggagaaaagtagagcgagactgagattacgaggagagcgatagagaaatgatagagagaaaaagcgaatagttagtggtttctctctatcaTTTCTCTATCGCTCTCCTCGTAatctcagtctcgctctacttttctccaatggcttcagacaagaagatcaagatcggaatcaacggatttggaaggatcgggcgtttggtggctagggttgctcttctgaggaacgatgttgagcttgttgctgttaac | 3' | 357 | 45.60 | -347.10 | 2.10 | 1.01 | 13.27 | 18.90 | middle |
| mul-miR78 | ATTGACGGTTTGAGACACGAGATT | 24 | aaattgtaccttgaacgtttgaattggtagcaattaaggtccaatagctaaagaatgataaATTGACAGTTTGAGACACGAGATTaacacatgaaatgcaaagcttgccatttttcgaactacgtcgtgtgtgaatttcaTGTCTCAAACTGTCAATTAATaatgctcgttagcaattggaagactgtatgcgatcaatatcctcgttgttgaagtacaaata | 5' | 217 | 35.00 | -74 | 0.90 | 33.73 | 16.59 | 12.93 | middle |
| mul-miR79 | GCTCGCTACTCTTTCTGTCAGT | 22 | tattttgtgggacatagaaattgacagaagagagtgagcacacagaggactttagcataagagtatgctattgcttttgcgtGCTCGCTACTCTTTCTGTCAGTttccactgccggaaatttactccctttttcatcgtctttggttccggcctgcaatctttgttccattaatcttAAATATCTCTCTCTCTCTCTCt | 5' | 91 | 41.20 | -71.80 | 0.90 | 1154.75 | 1240.91 | 254.64 | middle |
| mul-miR80 | TTGCCAAAGGAGATCTGCCCAG | 22 | cgcattgcaggtgaattgcaGGGCAGTTTCCTTTGACAAGCtgtgctcaaatattatgcatgcaagaggttcatgattatattccagtgaagtagtacagtTTGCCAAAGGAGATCTGCCCAGtaattctagtcctgcaatatc | 3' | 138 | 42.40 | -60.10 | 1.00 | 65.44 | 96.69 | 68.63 | middle |
| mul-miR81 | GACAAGAAGATCAAGATCGGAATC | 24 | atccttccaaatccgttgattccgatcttgatcttcttgtctgaagccattggagaaaagtagagcgagactgagattacgaggagagcgatagagaaatgatagagagaaaaagcgaatagttagtggtttctctctatcatttctctatcgctctcctcgtaatctcagtctcgctctacttttctccaatggcTTCAGACAAGAAGATCAAGATCGGAATCaacggatttggaaggatcgggcgtttggtggctagggttgctcttctgaggaacgatgttgagcttgttgctgttaacgatccctttatcaacactgattacatgacctacatgttcaagttcgattccgtacacggacaatggaagcaccatgacgtcaaggtcaaggactccaagacccttcttttcggcgaga | 3' | 241 | 44.80 | -264.10 | 1.40 | 9.06 | 27.49 | 34.81 | middle |
| mul-miR82 | AACGTCTTAGAATGAGTTCATGATC | 25 | gaaattttgatcattgacttttaaAACGTCTTAGAATGAGTTCATGATCaaacaattatgcccttaaaattacaaaattaacttcaatggaaccgcacatagtcccacgcggcttttttcgttgaggttttaacaattgaatggacaaaagggactaatttgagataacttgatagattaggactcattttgagaCGTTTTAAAAGTCAAGGATGAaagtggg | 5' | 217 | 33.60 | -77.90 | 1.00 | 20.14 | 7.58 | 7.96 | middle |
| mul-miR83 | GTCAGCGCTGCACTCAATTAT | 21 | ctaaacgatccagataaatgaaacgtcattgagtgcagcgttgatgaatgttcaatttagaagtgatttccaatcttcatggttggctgtggctttgggaaattttcGTCAGCGCTGCACTCAATTATgtttctatccttttgggacattgttgct | 3' | 150 | 39.70 | -65.70 | 1.10 | 9.06 | 14.22 | 7.96 | middle |
| mul-miR84 | TGCTCTGTCCGTTCCCTGCCT | 21 | cacagccagggaagaggcagtgcatgagtagagccacaacagcattccttcttctgttgaaggctccatccaTGCTCTGTCCGTTCCCTGCCTtat | 3' | 90 | 53.10 | -43.60 | 0.90 | 17.11 | 21.80 | 40.78 | middle |
| mul-miR85 | TTATGAGTTGTGGTGTCGGGCACT | 24 | tgggcatcagtggtATCCGACACCACAATTCATATAGAgtaggtctcatataggtgcaactgcaacttatcttTTATGAGTTGTGGTGTCGGGCACTactgatatcctttaccatttttcttcaaagaattt | 3' | 106 | 39.40 | -58.40 | 1.10 | 10.07 | 7.58 | 8.95 | middle |
| mul-miR86 | TGTAGAATCCATGGCTTCCT | 20 | tgtccagtgatcgagaatcgctcgatTGCAGAGCTTCGCACGGCTTTgcgaaggcgatgaagaggacgaagaagaaggataagaagccccgactttgatagatttaaccggttttctggcttcgggatttcggcaaattgggtaggaagccatggattctttcagcttccgtgaatttccctcgcgttttgattcagagcTGTAGAATCCATGGCTTCCTacccaatttgccgaaatcccgaagccagaaaaccggttaaatctatcaaagtcggggcttcttatccttcttcttcgtcctcttcatcgccttcgcaaagccgtgcgaagctctgcaatcgagcgattctcgatcactggacaacg | 3' | 363 | 49.20 | -343.50 | 1.90 | 6.54 | 11.85 | 29.34 | middle |

HairpinLen: pre-miRNA length

CG%:GC Content

dG: Free energy

MFEI(Minimum folding free energy index): = -dG*100/mirLen/CG%.
