# Supplementary material for: MicroRNA Profiling During Mulberry (Morus atropurpurea Roxb) Fruit Development and Regulatory Pathway of miR477 for Anthocyanin Accumulation
Source: Front Plant Sci. 2021 Sep 8;12:687364. doi: 10.3389/fpls.2021.687364 (PMC8455890; doi:10.3389/fpls.2021.687364)
Supplement: Supplementary Table 2 — Predicted target genes of the DE miRNAs and their Gene Ontology and KEGG analysis. [file Table_2.DOC]

Supplementary Table 2. Predicted target genes of the DE miRNAs and their Gene Ontology and KEGG analysis

| **miRNA ID** | **Target transcript ID** | **Target gene annoation** | **Score** | **GO** | **KEGG** |
| --- | --- | --- | --- | --- | --- |
| mul-miR1511 | comp58427_c0 | Retrovirus-related Pol polyprotein from transposon TNT 1-94 | 3 | GO:0004190(aspartic-type endopeptidase activity);  GO:0003677(DNA binding);  GO:0004519(endonuclease activity);  GO:0003964(RNA-directed DNA polymerase activity);  GO:0008270(zinc ion binding);  GO:0015074(DNA integration) | |
|  | comp22084_c0 | Fe(2+) transport protein 1 | 4 | GO:0016021(integral to membrane);  GO:0005886(plasma membrane);  GO:0005385(zinc ion transmembrane transporter activity);  GO:0015691(cadmium ion transport);  GO:0006826(iron ion transport) | |
|  | comp55868_c0 | Ectonucleotide pyrophosphatase/phosphodiesterase  family member 1 | 4 | GO:0016323(basolateral plasma membrane);  GO:0009986(cell surface);  GO:0005615(extracellular space);  GO:0016021(integral to membrane);  GO:0050656(3'-phosphoadenosine 5'-phosphosulfate binding);  GO:0005524(ATP binding);  GO:0005158(insulin receptor binding);  GO:0046872(metal ion binding);  GO:0003676(nucleic acid binding);  GO:0047429(nucleoside-triphosphate diphosphatase activity);  GO:0004551(nucleotide diphosphatase activity);  GO:0004528(phosphodiesterase I activity);  GO:0030247(polysaccharide binding);  GO:0042803(protein homodimerization activity);  GO:0005044(scavenger receptor activity);  GO:0050427(3'-phosphoadenosine 5'-phosphosulfate metabolic process);  GO:0031214(biomineral tissue development);  GO:0030643(cellular phosphate ion homeostasis);  GO:0032869(cellular response to insulin stimulus);  GO:0006091(generation of precursor metabolites and energy);  GO:0006955(immune response);  GO:0030505(inorganic diphosphate transport);  GO:0030308(negative regulation of cell growth);  GO:0045599(negative regulation of fat cell differentiation);  GO:0046325(negative regulation of glucose import);  GO:0045719(negative regulation of glycogen biosynthetic process);  GO:0046627(negative regulation of insulin receptor signaling pathway);  GO:0031953(negative regulation of protein autophosphorylation);  GO:0009143(nucleoside triphosphate catabolic process);  GO:0006796(phosphate metabolic process);  GO:0030500(regulation of bone mineralization);  GO:0030730(sequestering of triglyceride);  GO:0006767(water-soluble vitamin metabolic process) | ko00230(Purine metabolism);  ko00500(Starch and sucrose metabolism);  ko00740(Riboflavin metabolism);  ko00760(Nicotinate and nicotinamide metabolism);  ko00770(Pantothenate and CoA biosynthesis) |
|  | comp60162_c0 | Copia protein | 4 |  |  |
| mul-miR156c-3p | comp242288_c0 | Proline-rich receptor-like protein kinase PERK2 | 3.5 | GO:0016021(integral to membrane);  GO:0005886(plasma membrane);  GO:0005524(ATP binding);  GO:0004674(protein serine/threonine kinase activity);  GO:0004872(receptor activity) | |
|  | comp49340_c0 | WD repeat-containing protein 44 | 3.5 | GO:0005829(cytosol);  GO:0010008(endosome membrane);  GO:0005794(Golgi apparatus);  GO:0048471(perinuclear region of cytoplasm) | |
|  | comp53064_c0 | Coiled-coil domain-containing protein 25 | 4 |  |  |
| mul-miR156e | comp8261_c0 | Squamosa promoter-binding-like protein 16 | 3 | GO:0005634(nucleus);  GO:0003677(DNA binding);  GO:0046872(metal ion binding);  GO:0006355(regulation of transcription, DNA-dependent);  GO:0006351(transcription, DNA-dependent) | |
|  | comp17312_c0 | Histidine-containing phosphotransfer protein 1 | 4 | GO:0005829(cytosol);  GO:0005634(nucleus);  GO:0009927(histidine phosphotransfer kinase activity);  GO:0043424(protein histidine kinase binding);  GO:0051301(cell division);  GO:0016049(cell growth);  GO:0009736(cytokinin mediated signaling pathway);  GO:0009553(embryo sac development);  GO:0080036(regulation of cytokinin mediated signaling pathway);  GO:0006950(response to stress);  GO:0000160(two-component signal transduction system ) | |
|  | comp48514_c0 | Cysteine synthase 2 | 4 | GO:0005739(mitochondrion);  GO:0004124(cysteine synthase activity);  GO:0030170(pyridoxal phosphate binding);  GO:0016740(transferase activity);  GO:0006535(cysteine biosynthetic process from serine) | ko00270(Cysteine and methionine metabolism);  ko00450(Selenoamino acid metabolism);  ko00920(Sulfur metabolism) |
| mul-miR156e-3p | comp59471_c0 | Mediator of RNA polymerase II transcription subunit 16 | 4 |  |  |
|  | comp37654_c0 | 30S ribosomal protein S17 | 3 | GO:0005840(ribosome);  GO:0019843(rRNA binding);  GO:0003735(structural constituent of ribosome);  GO:0006412(translation) | ko03010(Ribosome) |
|  | comp35348_c0 | 50S ribosomal protein L1, chloroplastic | 3.5 | GO:0009507(chloroplast);  GO:0015934(large ribosomal subunit);  GO:0019843(rRNA binding);  GO:0003735(structural constituent of ribosome);  GO:0006412(translation) | ko03010(Ribosome) |
|  | comp946856_c0 | BTB/POZ domain-containing protein | 3.5 |  |  |
|  | comp13603_c0 | UDP-glycosyltransferase 75B1 | 3.5 | GO:0005856(cytoskeleton);  GO:0048471(perinuclear region of cytoplasm);  GO:0009524(phragmoplast);  GO:0047215(indole-3-acetate beta-glucosyltransferase activity);  GO:0005515(protein binding);  GO:0080002(UDP-glucose:4-aminobenzoate acylglucosyltransferase activity);  GO:0009920(cell plate formation involved in plant-type cell wall biogenesis);  GO:0046482(para-aminobenzoic acid metabolic process);  GO:0009751(response to salicylic acid stimulus) | ko00942(Anthocyanin biosynthesis) |
|  | comp44787_c0 | UDP-galactose/UDP-glucose transporter 3 | 3.5 |  |  |
|  | comp35969_c0 | 40S ribosomal protein S8 | 4 | GO:0005840(ribosome);  GO:0003735(structural constituent of ribosome);  GO:0006412(translation) | ko03010(Ribosome) |
|  | comp80572_c0 | 40S ribosomal protein S8 | 4 | GO:0005840(ribosome);  GO:0003735(structural constituent of ribosome);  GO:0006412(translation) | ko03010(Ribosome) |
|  | comp76932_c0 | Superoxide dismutase [Mn] | 4 | GO:0005759(mitochondrial matrix);  GO:0046872(metal ion binding);  GO:0004784(superoxide dismutase activity);  GO:0006801(superoxide metabolic process) | |
|  | comp20159_c0 | DNA-directed RNA polymerase 1B | 4 | GO:0005739(mitochondrion);  GO:0003677(DNA binding);  GO:0003899(DNA-directed RNA polymerase activity) | |
|  | comp54271_c0 | Fimbrin-like protein 2 | 4 | GO:0005856(cytoskeleton);  GO:0005829(cytosol);  GO:0051015(actin filament binding);  GO:0030036(actin cytoskeleton organization);  GO:0009846(pollen germination);  GO:0009860(pollen tube growth) | |
|  | comp44485_c0 | Pyridoxal biosynthesis protein PDX2 | 4 | GO:0005829(cytosol);  GO:0004359(glutaminase activity);  GO:0046982(protein heterodimerization activity);  GO:0016740(transferase activity);  GO:0006541(glutamine metabolic process);  GO:0008615(pyridoxine biosynthetic process) | ko00750(Vitamin B6 metabolism) |
|  | comp533517_c0 | Upstream activation factor subunit spp27 | 4 | GO:0005829(cytosol);  GO:0000500(RNA polymerase I upstream activating factor complex);  GO:0005515(protein binding);  GO:0006355(regulation of transcription, DNA-dependent);  GO:0006360(transcription from RNA polymerase I promoter) | |
|  | comp46880_c0 | Gamma carbonic anhydrase 1, mitochondrial | 4 |  | ko00350(Tyrosine metabolism);  ko00624(1- and 2-Methylnaphthalene degradation);  ko00632(Benzoate degradation via CoA ligation);  ko00642(Ethylbenzene degradation);  ko00903(Limonene and pinene degradation) |
|  | comp59463_c0 | TMV resistance protein N | 4 | GO:0005737(cytoplasm);  GO:0031224(intrinsic to membrane);  GO:0005524(ATP binding);  GO:0005515(protein binding);  GO:0004888(transmembrane signaling receptor activity);  GO:0006915(apoptosis);  GO:0009626(plant-type hypersensitive response) | |
|  | comp54265_c0 | Threonine synthase 1 | 4 | GO:0009507(chloroplast);  GO:0005829(cytosol);  GO:0030170(pyridoxal phosphate binding);  GO:0004795(threonine synthase activity);  GO:0009088(threonine biosynthetic process) | ko00260(Glycine,ko serine and threonine metabolism);ko00750(Vitamin B6 metabolism) |
|  | comp58525_c0 | Beta-(1,2)-xylosyltransferase | 4 | GO:0032580(Golgi cisterna membrane);  GO:0005797(Golgi medial cisterna);  GO:0016021(integral to membrane);  GO:0050513(glycoprotein 2-beta-D-xylosyltransferase activity);  GO:0031204(posttranslational protein targeting to membrane, translocation);  GO:0006487(protein N-linked glycosylation) | ko00510(N-Glycan biosynthesis) |
|  | comp13392_c0 | Ribosome biogenesis protein BRX1 homolog | 4 | GO:0005730(nucleolus);  GO:0004812(aminoacyl-tRNA ligase activity);  GO:0005524(ATP binding);  GO:0042254(ribosome biogenesis) | |
| mul-miR156t | comp8261_c0 | Squamosa promoter-binding-like protein 16 | 2 | GO:0005634(nucleus);  GO:0003677(DNA binding);  GO:0046872(metal ion binding);  GO:0006355(regulation of transcription, DNA-dependent);  GO:0006351(transcription, DNA-dependent) | |
|  | comp40489_c0 | Squamosa promoter-binding-like protein 4 | 2.5 | GO:0005737(cytoplasm);  GO:0005634(nucleus);  GO:0003677(DNA binding);  GO:0046872(metal ion binding);  GO:0006355(regulation of transcription, DNA-dependent);  GO:0010321(regulation of vegetative phase change);  GO:0006351(transcription, DNA-dependent) | |
|  | comp48514_c0 | Cysteine synthase 2 | 3 | GO:0005739(mitochondrion);  GO:0004124(cysteine synthase activity);  GO:0030170(pyridoxal phosphate binding);  GO:0016740(transferase activity);  GO:0006535(cysteine biosynthetic process from serine) | ko00270(Cysteine and methionine metabolism);  ko00450(Selenoamino acid metabolism);  ko00920(Sulfur metabolism) |
|  | comp57347_c0 | Glyceraldehyde-3-phosphate dehydrogenase | 4 | GO:0005737(cytoplasm);  GO:0004365(glyceraldehyde-3-phosphate dehydrogenase );  GO:0051287(NAD binding);  GO:0006096(glycolysis) | ko00010(Glycolysis / Gluconeogenesis);  ko05010(Alzheimer's disease) |
|  | comp17312_c0 | Histidine-containing phosphotransfer protein 1 | 4 | GO:0005829(cytosol);  GO:0005634(nucleus);  GO:0009927(histidine phosphotransfer kinase activity);  GO:0043424(protein histidine kinase binding);  GO:0051301(cell division);  GO:0016049(cell growth);  GO:0009736(cytokinin mediated signaling pathway);  GO:0009553(embryo sac development);  GO:0080036(regulation of cytokinin mediated signaling pathway);  GO:0006950(response to stress);  GO:0000160(two-component signal transduction system ) | |
| mul-miR160f-3p | comp60442_c0 | Retrovirus-related Pol polyprotein from transposon 17.6 | 4 | GO:0004190(aspartic-type endopeptidase activity);  GO:0003677(DNA binding);  GO:0004519(endonuclease activity);  GO:0003723(RNA binding);  GO:0003964(RNA-directed DNA polymerase activity);  GO:0015074(DNA integration);  GO:0006278(RNA-dependent DNA replication) | |
| mul-miR166a-5p | comp57479_c0 | Mitogen-activated protein kinase kinase kinase YODA | 3.5 |  | ko04011(MAPK signaling pathway - yeast) |
|  | comp49725_c0 | Protein kinase APK1B | 4 | GO:0009507(chloroplast);  GO:0005886(plasma membrane);  GO:0005524(ATP binding);  GO:0004674(protein serine/threonine kinase activity);  GO:0004713(protein tyrosine kinase activity) | |
| mul-miR167b-5p | comp67450_c0 | Cysteine proteinase RD19a | 3 | GO:0005634(nucleus);  GO:0005773(vacuole);  GO:0004197(cysteine-type endopeptidase activity);  GO:0042742(defense response to bacterium);  GO:0006508(proteolysis);  GO:0009651(response to salt stress) | |
|  | comp41890_c0 | Xin actin-binding repeat-containing protein 2 | 3.5 | GO:0030054(cell junction);  GO:0003779(actin binding);  GO:0008270(zinc ion binding);  GO:0030036(actin cytoskeleton organization) | |
| mul-miR167c-3p | comp60317_c0 | Fanconi-associated nuclease 1 homolog | 3.5 | GO:0016818(hydrolase activity, acting on acid anhydrides);  GO:0004518(nuclease activity);  GO:0003676(nucleic acid binding);  GO:0008270(zinc ion binding) | |
| mul-miR168d | comp41958_c0 | Protein argonaute 1 | 3.5 | GO:0005829(cytosol);  GO:0005634(nucleus);  GO:0030529(ribonucleoprotein complex);  GO:0004521(endoribonuclease activity);  GO:0035198(miRNA binding);  GO:0035197(siRNA binding);  GO:0048830(adventitious root development);  GO:0009850(auxin metabolic process);  GO:0009793(embryo development ending in seed dormancy);  GO:0035195(gene silencing by miRNA);  GO:0009965(leaf morphogenesis);  GO:0006355(regulation of transcription, DNA-dependent);  GO:0006417(regulation of translation);  GO:0009733(response to auxin stimulus);  GO:0010218(response to far red light);  GO:0016246(RNA interference);  GO:0048864(stem cell development);  GO:0006351(transcription, DNA-dependent);  GO:0009616(virus induced gene silencing) | |
| mul-miR169a-3p | comp21843_c0 | Phosphatidylinositol 4-kinase beta 1 | 3.5 | GO:0030659(cytoplasmic vesicle membrane);  GO:0005829(cytosol);  GO:0005634(nucleus);  GO:0035619(root hair tip);  GO:0004430(1-phosphatidylinositol 4-kinase activity);  GO:0043424(protein histidine kinase binding);  GO:0017137(Rab GTPase binding);  GO:0048015(phosphatidylinositol-mediated signaling);  GO:0009860(pollen tube growth);  GO:0048768(root hair cell tip growth) | ko00562(Inositol phosphate metabolism);  ko04070(Phosphatidylinositol signaling system) |
|  | comp58368_c0 | Protein kinase and PP2C-like domain-containing protein | 3.5 | GO:0005524(ATP binding);  GO:0046872(metal ion binding);  GO:0004721(phosphoprotein phosphatase activity);  GO:0004674(protein serine/threonine kinase activity) | |
| mul-miR169b | comp34325_c0 | Nuclear transcription factor Y subunit A-1 | 3 | GO:0016602(CCAAT-binding factor complex);  GO:0003677(DNA binding);  GO:0003700(sequence-specific DNA binding transcription factor activity);  GO:0048510(regulation of timing of transition from vegetative to reproductive phase) | ko04612(Antigen processing and presentation) |
|  | comp43087_c0 | Nuclear transcription factor Y subunit A-3 | 3.5 | GO:0016602(CCAAT-binding factor complex);  GO:0003677(DNA binding);  GO:0003700(sequence-specific DNA binding transcription factor activity) | ko04612(Antigen processing and presentation) |
|  | comp9026_c0 | Nuclear transcription factor Y subunit A-8 | 4 | GO:0016602(CCAAT-binding factor complex);  GO:0003677(DNA binding);  GO:0003700(sequence-specific DNA binding transcription factor activity) | ko04612(Antigen processing and presentation) |
| mul-miR169r | comp48621_c0 | Nuclear transcription factor Y subunit A-7 | 4 | GO:0005634(nucleus);  GO:0003677(DNA binding);  GO:0003700(sequence-specific DNA binding transcription factor activity);  GO:0045892(negative regulation of transcription, DNA-dependent) | ko04612(Antigen processing and presentation) |
|  | comp34325_c0 | Nuclear transcription factor Y subunit A-1 | 4 | GO:0016602(CCAAT-binding factor complex);  GO:0003677(DNA binding);  GO:0003700(sequence-specific DNA binding transcription factor activity);  GO:0048510(regulation of timing of transition from vegetative to reproductive phase) | ko04612(Antigen processing and presentation) |
| mul-miR2111a | comp52451_c0 | Palmitoyl-acyl carrier protein thioesterase | 3.5 |  | ko00061(Fatty acid biosynthesis) |
| mul-miR319a | comp57886_c0 | Transposon Ty3-G Gag-Pol polyprotein | 2 | GO:0005737(cytoplasm);  GO:0005634(nucleus);  GO:0004190(aspartic-type endopeptidase activity);  GO:0005524(ATP binding);  GO:0003677(DNA binding);  GO:0003887(DNA-directed DNA polymerase activity);  GO:0004523(ribonuclease H activity);  GO:0003723(RNA binding);  GO:0003964(RNA-directed DNA polymerase activity);  GO:0008270(zinc ion binding);  GO:0015074(DNA integration);  GO:0006310(DNA recombination);  GO:0006278(RNA-dependent DNA replication);  GO:0046797(viral procapsid maturation) | |
|  | comp1035934_c0 | Transcription factor PCF5 | 3 | GO:0005634(nucleus);  GO:0003677(DNA binding);  GO:0007275(multicellular organismal development);  GO:0006355(regulation of transcription, DNA-dependent);  GO:0006351(transcription, DNA-dependent) | |
|  | comp131416_c0 | Transcription factor TCP2 | 3.5 | GO:0005634(nucleus);  GO:0003677(DNA binding);  GO:0048366(leaf development);  GO:0006355(regulation of transcription, DNA-dependent);  GO:0006351(transcription, DNA-dependent) | |
|  | comp59876_c0 | Probable receptor-like protein kinase | 4 | GO:0016021(integral to membrane);  GO:0005886(plasma membrane);  GO:0005524(ATP binding);  GO:0004674(protein serine/threonine kinase activity) | ko04210(Apoptosis);  ko04620(Toll-like receptor signaling pathway);  ko04722(Neurotrophin signaling pathway);  ko05140(Leishmaniasis);  ko05142(Chagas disease) |
|  | comp58149_c0 | Transposon Ty3-I Gag-Pol polyprotein | 4 | GO:0005737(cytoplasm);  GO:0005634(nucleus);  GO:0004190(aspartic-type endopeptidase activity);  GO:0005524(ATP binding);  GO:0003677(DNA binding);  GO:0003887(DNA-directed DNA polymerase activity);  GO:0004523(ribonuclease H activity);  GO:0003723(RNA binding);  GO:0003964(RNA-directed DNA polymerase activity);  GO:0008270(zinc ion binding);  GO:0015074(DNA integration);  GO:0006310(DNA recombination);  GO:0006278(RNA-dependent DNA replication);  GO:0046797(viral procapsid maturation) | |
| mul-miR319a-3p | comp57886_c0 | Transposon Ty3-G Gag-Pol polyprotein | 2 | GO:0005737(cytoplasm);  GO:0005634(nucleus);  GO:0004190(aspartic-type endopeptidase activity);  GO:0005524(ATP binding);  GO:0003677(DNA binding);  GO:0003887(DNA-directed DNA polymerase activity);  GO:0004523(ribonuclease H activity);  GO:0003723(RNA binding);  GO:0003964(RNA-directed DNA polymerase activity);  GO:0008270(zinc ion binding);  GO:0015074(DNA integration);  GO:0006310(DNA recombination);  GO:0006278(RNA-dependent DNA replication);  GO:0046797(viral procapsid maturation) | |
|  | comp131416_c0 | Transcription factor TCP2 | 2.5 | GO:0005634(nucleus);  GO:0003677(DNA binding);  GO:0048366(leaf development);  GO:0006355(regulation of transcription, DNA-dependent);  GO:0006351(transcription, DNA-dependent) | |
|  | comp1035934_c0 | Transcription factor PCF5 | 3 | GO:0005634(nucleus);  GO:0003677(DNA binding);  GO:0007275(multicellular organismal development);  GO:0006355(regulation of transcription, DNA-dependent);  GO:0006351(transcription, DNA-dependent) | |
|  | comp58149_c0 | Transposon Ty3-I Gag-Pol polyprotein | 3 | GO:0005737(cytoplasm);  GO:0005634(nucleus);  GO:0004190(aspartic-type endopeptidase activity);  GO:0005524(ATP binding);  GO:0003677(DNA binding);  GO:0003887(DNA-directed DNA polymerase activity);  GO:0004523(ribonuclease H activity);  GO:0003723(RNA binding);  GO:0003964(RNA-directed DNA polymerase activity);  GO:0008270(zinc ion binding);  GO:0015074(DNA integration);  GO:0006310(DNA recombination);  GO:0006278(RNA-dependent DNA replication);  GO:0046797(viral procapsid maturation) | |
|  | comp13131_c0 | 60S ribosomal protein L27 | 4 | GO:0005840(ribosome);  GO:0003735(structural constituent of ribosome);  GO:0006412(translation) | ko03010(Ribosome) |
|  | comp40675_c0 | Transcription factor UNE12 | 4 | GO:0005634(nucleus);  GO:0003677(DNA binding);  GO:0009567(double fertilization forming a zygote and endosperm);  GO:0007275(multicellular organismal development);  GO:0031347(regulation of defense response);  GO:0006355(regulation of transcription, DNA-dependent);  GO:0006351(transcription, DNA-dependent) | |
| mul-miR390a-5p | comp55997_c0 | Probable protein phosphatase 2C 74 | 4 | GO:0005634(nucleus);  GO:0046872(metal ion binding);  GO:0004721(phosphoprotein phosphatase activity) | |
| mul-miR394a-5p | comp50906_c0 | Lipid phosphate phosphatase 2 | 3.5 | GO:0016021(integral to membrane);  GO:0005886(plasma membrane);  GO:0008195(phosphatidate phosphatase activity);  GO:0009738(abscisic acid mediated signaling pathway) | ko00561(Glycerolipid metabolism);  ko00564(Glycerophospholipid metabolism);  ko00565(Ether lipid metabolism);  ko00600(Sphingolipid metabolism);  ko04666(Fc gamma R-mediated phagocytosis) |
|  | comp180321_c0 | Protein SPIRAL1-like 5 | 4 |  |  |
|  | comp49286_c0 | F-box only protein 6 | 4 |  |  |
|  | comp59046_c2 | Protein ARABIDILLO 1 | 4 | GO:0005634(nucleus);  GO:0005515(protein binding);  GO:0048527(lateral root development) | |
|  | comp51017_c0 | F-box protein SKIP5 | 4 |  |  |
| mul-miR395 | comp41203_c0 | Sulfate transporter 2.2 | 2.5 | GO:0016021(integral to membrane);  GO:0008271(secondary active sulfate transmembrane transporter activity);  GO:0015293(symporter activity) | |
|  | comp50104_c0 | ATP sulfurylase 1 | 3 | GO:0009570(chloroplast stroma);  GO:0005886(plasma membrane);  GO:0005524(ATP binding);  GO:0004781(sulfate adenylyltransferase );  GO:0046686(response to cadmium ion);  GO:0000103(sulfate assimilation) | ko00230(Purine metabolism);  ko00450(Selenoamino acid metabolism);  ko00920(Sulfur metabolism) |
|  | comp55415_c1 | Heat shock 70 kDa protein 5 | 4 |  | ko03040(Spliceosome);  ko04010(MAPK signaling pathway);  ko04144(Endocytosis);  ko04612(Antigen processing and presentation) |
| mul-miR396a | comp53296_c1 | Glucan endo-1,3-beta-glucosidase-like protein | 2 | GO:0031225(anchored to membrane);  GO:0005886(plasma membrane);  GO:0009506(plasmodesma) | |
|  | comp1315001_c0 | Putative pentatricopeptide repeat-containing protein | 2.5 | GO:0005488(binding) |  |
|  | comp14222_c0 | Dual specificity protein kinase splB | 2.5 | GO:0005524(ATP binding);  GO:0004715(non-membrane spanning protein tyrosine kinase activity) | |
|  | comp59414_c0 | U-box domain-containing protein 3 | 3 | GO:0000151(ubiquitin ligase complex);  GO:0005488(binding);  GO:0004842(ubiquitin-protein ligase activity) | |
|  | comp10967_c0 | Pentatricopeptide repeat-containing protein | 3 | GO:0005739(mitochondrion) |  |
|  | comp52261_c0 | Eukaryotic translation initiation factor NCBP | 3.5 |  | ko04150(mTOR signaling pathway);  ko04910(Insulin signaling pathway) |
|  | comp229213_c0 | Probable WRKY transcription factor 21 | 3.5 | GO:0005634(nucleus);  GO:0005516(calmodulin binding);  GO:0043565(sequence-specific DNA binding);  GO:0003700(sequence-specific DNA binding transcription factor activity) | |
|  | comp43599_c0 | Annexin-like protein RJ4 | 3.5 | GO:0005509(calcium ion binding);  GO:0005544(calcium-dependent phospholipid binding) | |
|  | comp52103_c0 | Sulfite oxidase | 3.5 | GO:0005739(mitochondrion);  GO:0005777(peroxisome);  GO:0009055(electron carrier activity);  GO:0030151(molybdenum ion binding);  GO:0008482(sulfite oxidase activity);  GO:0015994(chlorophyll metabolic process);  GO:0010477(response to sulfur dioxide);  GO:0006790(sulfur compound metabolic process) | ko00920(Sulfur metabolism) |
|  | comp47361_c0 | Guanine deaminase | 3.5 | GO:0008892(guanine deaminase activity);  GO:0008270(zinc ion binding);  GO:0006144(purine base metabolic process) | ko00230(Purine metabolism) |
|  | comp49698_c0 | Indole-3-acetic acid-induced protein ARG7 | 3.5 |  |  |
|  | comp8661_c0 | Threonylcarbamoyladenosine tRNA methylthiotransferase | 3.5 | GO:0016021(integral to membrane);  GO:0051539(4 iron, 4 sulfur cluster binding);  GO:0046872(metal ion binding);  GO:0016740(transferase activity);  GO:0009451(RNA modification) | |
|  | comp1491414_c0 | Floral homeotic protein AGAMOUS | 3.5 | GO:0005634(nucleus);  GO:0043565(sequence-specific DNA binding);  GO:0003700(sequence-specific DNA binding transcription factor activity) | |
|  | comp58130_c0 | U5 small nuclear ribonucleoprotein 200 kDa helicase | 3.5 | GO:0071013(catalytic step 2 spliceosome);  GO:0005654(nucleoplasm);  GO:0005682(U5 snRNP);  GO:0005524(ATP binding);  GO:0008026(ATP-dependent helicase activity);  GO:0003676(nucleic acid binding);  GO:0000354(cis assembly of pre-catalytic spliceosome) | |
|  | comp1560109_c0 | Putative pentatricopeptide repeat-containing protein | 4 | GO:0005739(mitochondrion);  GO:0005488(binding) |  |
|  | comp1068401_c0 | Pentatricopeptide repeat-containing protein | 4 | GO:0009507(chloroplast);  GO:0005488(binding);  GO:0016556(mRNA modification) | |
|  | comp124848_c0 | Uncharacterized protein | 4 | GO:0009507(chloroplast);  GO:0046872(metal ion binding) |  |
|  | comp24467_c0 | Growth-regulating factor 1 | 4 |  |  |
|  | comp11910_c0 | E3 ubiquitin-protein ligase RFWD2 | 4 | GO:0005813(centrosome);  GO:0005829(cytosol);  GO:0005925(focal adhesion);  GO:0016607(nuclear speck);  GO:0016874(ligase activity);  GO:0005515(protein binding);  GO:0008270(zinc ion binding);  GO:0006977(DNA damage response, signal transduction by p53 class mediator resulting in cell cycle arrest) | ko04115(p53 signaling pathway);  ko04120(Ubiquitin mediated proteolysis) |
|  | comp55445_c1 | Probable 2-oxoglutarate/Fe(II)-dependent dioxygenase | 4 | GO:0005506(iron ion binding);  GO:0016706(oxidoreductase activity, acting on paired donors, with incorporation or reduction of molecular oxygen, 2-oxoglutarate as one donor, and incorporation of one atom each of oxygen into both donors);  GO:0016702(oxidoreductase activity, acting on single donors with incorporation of molecular oxygen, incorporation of two atoms of oxygen);  GO:0016740(transferase activity) | ko00270(Cysteine and methionine metabolism) |
|  | comp150889_c0 | Oryzain alpha chain | 4 | GO:0004197(cysteine-type endopeptidase activity);  GO:0006508(proteolysis) | |
|  | comp44090_c0 | Formin-like protein 20 | 4 | GO:0003779(actin binding);  GO:0004721(phosphoprotein phosphatase activity);  GO:0030036(actin cytoskeleton organization) | ko00564(Glycerophospholipid metabolism);  ko00565(Ether lipid metabolism);  ko04144(Endocytosis);  ko04666(Fc gamma R-mediated phagocytosis);  ko04912(GnRH signaling pathway) |
|  | comp46113_c0 | 60S ribosomal protein L14-2 | 4 | GO:0009507(chloroplast);  GO:0022625(cytosolic large ribosomal subunit);  GO:0005783(endoplasmic reticulum);  GO:0005730(nucleolus);  GO:0009506(plasmodesma);  GO:0005774(vacuolar membrane);  GO:0003735(structural constituent of ribosome);  GO:0006412(translation) | ko03010(Ribosome) |
|  | comp36466_c0 | Uncharacterized membrane protein | 4 | GO:0005789(endoplasmic reticulum membrane);  GO:0016021(integral to membrane) | |
|  | comp50748_c0 | Clustered mitochondria protein | 4 |  |  |
|  | comp50710_c0 | Reticulon-like protein B22 | 4 | GO:0005789(endoplasmic reticulum membrane);  GO:0016021(integral to membrane) | |
|  | comp70654_c0 | gamous-like MADS-box protein AGL80 | 4 | GO:0005634(nucleus);  GO:0005515(protein binding);  GO:0043565(sequence-specific DNA binding);  GO:0003700(sequence-specific DNA binding transcription factor activity) | |
|  | comp36219_c0 | Pentatricopeptide repeat-containing protein | 4 | GO:0009507(chloroplast);  GO:0005488(binding) |  |
|  | comp59756_c0 | Eukaryotic translation initiation factor 3 subunit C | 4 | GO:0005829(cytosol);  GO:0005852(eukaryotic translation initiation factor 3 complex);  GO:0005634(nucleus);  GO:0005515(protein binding);  GO:0003743(translation initiation factor activity) | |
|  | comp35872_c0 | Thiol protease aleurain | 4 | GO:0005773(vacuole);  GO:0004197(cysteine-type endopeptidase activity);  GO:0007568(aging);  GO:0006508(proteolysis) | ko04142(Lysosome) |
|  | comp8966_c0 | Putative zinc metalloprotease slr1821 | 4 | GO:0016021(integral to membrane);  GO:0005886(plasma membrane);  GO:0046872(metal ion binding);  GO:0004222(metalloendopeptidase activity);  GO:0006508(proteolysis) | |
|  | comp41166_c0 | Peroxiredoxin-2F | 4 | GO:0005759(mitochondrial matrix);  GO:0004601(peroxidase activity);  GO:0051920(peroxiredoxin activity);  GO:0046686(response to cadmium ion);  GO:0006979(response to oxidative stress) | |
|  | comp38675_c0 | High mobility group B protein 14 | 4 | GO:0005634(nucleus);  GO:0003677(DNA binding) |  |
|  | comp25041_c0 | Serine/threonine-protein kinase PEPKR2 | 4 | GO:0005524(ATP binding);  GO:0004674(protein serine/threonine kinase activity) | |
|  | comp35171_c0 | Inactive protein kinase SELMODRAFT_444075 | 4 | GO:0005524(ATP binding);  GO:0004672(protein kinase activity) | |
|  | comp49543_c0 | Cysteine proteinase RD21a | 4 | GO:0048046(apoplast);  GO:0009507(chloroplast);  GO:0009506(plasmodesma);  GO:0005773(vacuole);  GO:0004197(cysteine-type endopeptidase activity);  GO:0005515(protein binding);  GO:0006508(proteolysis) | |
|  | comp59374_c0 | Activating signal cointegrator 1 complex subunit 3 | 4 |  |  |
|  | comp22781_c0 | U-box domain-containing protein 25 | 4 | GO:0000151(ubiquitin ligase complex);  GO:0005488(binding);  GO:0004842(ubiquitin-protein ligase activity);  GO:0010200(response to chitin) | |
|  | comp13166_c0 | Methyl-CpG-binding domain-containing protein 10 | 4 | GO:0005829(cytosol);  GO:0005634(nucleus);  GO:0003677(DNA binding);  GO:0006355(regulation of transcription, DNA-dependent);  GO:0006351(transcription, DNA-dependent) | |
|  | comp56076_c0 | G-type lectin S-receptor-like serine/threonine-protein kinase | 4 | GO:0016021(integral to membrane);  GO:0044459(plasma membrane part);  GO:0005524(ATP binding);  GO:0005516(calmodulin binding);  GO:0004674(protein serine/threonine kinase activity);  GO:0004872(receptor activity);  GO:0005529(sugar binding);  GO:0048544(recognition of pollen) | |
|  | comp58311_c0 | Probable lysine-specific demethylase ELF6 | 4 | GO:0005634(nucleus);  GO:0003676(nucleic acid binding);  GO:0016702(oxidoreductase activity, acting on single donors with incorporation  of molecular oxygen, incorporation of two atoms of oxygen);  GO:0008270(zinc ion binding);  GO:0033169(histone H3-K9 demethylation);  GO:0048579(negative regulation of long-day photoperiodism, flowering);  GO:0048577(negative regulation of short-day photoperiodism, flowering);  GO:0006355(regulation of transcription, DNA-dependent);  GO:0009741(response to brassinosteroid stimulus);  GO:0006351(transcription, DNA-dependent);  GO:0009826(unidimensional cell growth) | |
|  | comp59012_c0 | Probable LRR receptor-like serine/threonine-protein kinase | 4 | GO:0016021(integral to membrane);  GO:0005524(ATP binding);  GO:0004674(protein serine/threonine kinase activity);  GO:0004872(receptor activity) | ko04210(Apoptosis);  ko04620(Toll-like receptor signaling pathway);  ko04722(Neurotrophin signaling pathway);  ko05140(Leishmaniasis);  ko05142(Chagas disease) |
|  | comp48133_c0 | Splicing factor 3B subunit 1 | 4 | GO:0071013(catalytic step 2 spliceosome);  GO:0016607(nuclear speck);  GO:0005689(U12-type spliceosomal complex);  GO:0005488(binding);  GO:0000398(nuclear mRNA splicing, via spliceosome) | |
| mul-miR397-5p | comp24369_c0 | Laccase-4 | 2 | GO:0048046(apoplast);  GO:0005507(copper ion binding);  GO:0052716(hydroquinone:oxygen oxidoreductase activity);  GO:0008471(laccase activity);  GO:0009809(lignin biosynthetic process);  GO:0046274(lignin catabolic process);  GO:0009834(secondary cell wall biogenesis) | ko00910(Nitrogen metabolism) |
|  | comp41524_c0 | Zinc finger CCCH domain-containing protein 24 | 2.5 | GO:0003677(DNA binding);  GO:0008270(zinc ion binding) |  |
|  | comp1199328_c0 | Laccase-4 | 3 | GO:0048046(apoplast);  GO:0005507(copper ion binding);  GO:0052716(hydroquinone:oxygen oxidoreductase activity);  GO:0008471(laccase activity);  GO:0009809(lignin biosynthetic process);  GO:0046274(lignin catabolic process);  GO:0009834(secondary cell wall biogenesis) | ko00910(Nitrogen metabolism) |
|  | comp25234_c0 | Laccase-13 | 3 | GO:0048046(apoplast);  GO:0005507(copper ion binding);  GO:0052716(hydroquinone:oxygen oxidoreductase activity);  GO:0008471(laccase activity);  GO:0046274(lignin catabolic process) | ko00910(Nitrogen metabolism) |
|  | comp59901_c0 | Laccase-7 | 4 | GO:0048046(apoplast);  GO:0005507(copper ion binding);  GO:0052716(hydroquinone:oxygen oxidoreductase activity);  GO:0008471(laccase activity);  GO:0046274(lignin catabolic process) | ko00910(Nitrogen metabolism) |
|  | comp55145_c0 | Probable phenylalanine--tRNA ligase beta subunit | 4 | GO:0005829(cytosol);  GO:0005524(ATP binding);  GO:0000287(magnesium ion binding);  GO:0004826(phenylalanine-tRNA ligase activity);  GO:0003723(RNA binding);  GO:0006432(phenylalanyl-tRNA aminoacylation) | ko00970(Aminoacyl-tRNA biosynthesis) |
| mul-miR398a | comp744225_c0 | Copper transporter 3 | 3 | GO:0016021(integral to membrane);  GO:0005375(copper ion transmembrane transporter activity) | |
| mul-miR399b | comp47988_c0 | Double-stranded RNA-binding protein 1 | 3.5 | GO:0010445(nuclear dicing body);  GO:0003725(double-stranded RNA binding);  GO:0035198(miRNA binding);  GO:0005515(protein binding);  GO:0035279(mRNA cleavage involved in gene silencing by miRNA);  GO:0031054(pre-miRNA processing);  GO:0010267(production of ta-siRNAs involved in RNA interference);  GO:0009737(response to abscisic acid stimulus);  GO:0009733(response to auxin stimulus);  GO:0009735(response to cytokinin stimulus) | |
|  | comp744021_c0 | MLO-like protein 3 | 3.5 | GO:0016021(integral to membrane);  GO:0005516(calmodulin binding);  GO:0008219(cell death);  GO:0006952(defense response);  GO:0009607(response to biotic stimulus) | |
|  | comp55781_c0 | ATP-dependent helicase rhp16 | 4 | GO:0000109(nucleotide-excision repair complex);  GO:0005524(ATP binding);  GO:0003677(DNA binding);  GO:0004386(helicase activity);  GO:0005515(protein binding);  GO:0008270(zinc ion binding);  GO:0034644(cellular response to UV);  GO:0006289(nucleotide-excision repair) | ko00230(Purine metabolism) |
|  | comp54037_c0 | Histone deacetylase 5 | 4 | GO:0005737(cytoplasm);  GO:0005634(nucleus);  GO:0034739(histone deacetylase activity );  GO:0032041(NAD-dependent histone deacetylase activity );  GO:0046969(NAD-dependent histone deacetylase activity );  GO:0046970(NAD-dependent histone deacetylase activity );  GO:0006355(regulation of transcription, DNA-dependent);  GO:0006351(transcription, DNA-dependent) | |
|  | comp53221_c0 | Protein SCAI | 4 | GO:0005737(cytoplasm);  GO:0016021(integral to membrane);  GO:0005634(nucleus);  GO:0005515(protein binding);  GO:0003714(transcription corepressor activity);  GO:0030336(negative regulation of cell migration);  GO:0035024(negative regulation of Rho protein signal transduction);  GO:0006355(regulation of transcription, DNA-dependent);  GO:0006351(transcription, DNA-dependent) | |
|  | comp26650_c0 | Heat stress transcription factor A-4b | 4 | GO:0005737(cytoplasm);  GO:0005634(nucleus);  GO:0043565(sequence-specific DNA binding);  GO:0003700(sequence-specific DNA binding transcription factor activity);  GO:0006950(response to stress) | |
|  | comp37234_c0 | ABC transporter B family member 1 | 4 | GO:0016021(integral to membrane);  GO:0005886(plasma membrane);  GO:0009506(plasmodesma);  GO:0005524(ATP binding);  GO:0042626(ATPase activity, coupled to transmembrane movement of substances);  GO:0010329(auxin efflux transmembrane transporter activity);  GO:0005515(protein binding);  GO:0043481(anthocyanin accumulation in tissues in response to UV light);  GO:0009926(auxin polar transport);  GO:0009640(photomorphogenesis);  GO:0009958(positive gravitropism);  GO:0008361(regulation of cell size);  GO:0009637(response to blue light);  GO:0009624(response to nematode);  GO:0048443(stamen development) | ko02010(ABC transporters) |
| mul-miR4414a-5p | comp51481_c0 | UPF0369 protein | 4 |  |  |
| mul-miR472b | comp53434_c0 | Probable anion transporter 3 | 1 | GO:0031969(chloroplast membrane);  GO:0016021(integral to membrane);  GO:0005315(inorganic phosphate transmembrane transporter activity) | |
|  | comp29827_c0 | UDP-glycosyltransferase 88A1 | 3 | GO:0005829(cytosol);  GO:0080045(quercetin 3'-O-glucosyltransferase activity);  GO:0080043(quercetin 3-O-glucosyltransferase activity);  GO:0080046(quercetin 4'-O-glucosyltransferase activity);  GO:0080044(quercetin 7-O-glucosyltransferase activity) | |
|  | comp56979_c1 | Chaperone protein ClpB3 | 3 | GO:0009570(chloroplast stroma);  GO:0005524(ATP binding);  GO:0017111(nucleoside-triphosphatase activity);  GO:0009658(chloroplast organization);  GO:0016485(protein processing);  GO:0009408(response to heat) | |
|  | comp54632_c0 | Uncharacterized protein | 3 | GO:0005886(plasma membrane) |  |
|  | comp49947_c0 | Mediator of RNA polymerase II transcription subunit 4 | 3 |  | ko04622(RIG-I-like receptor signaling pathway) |
|  | comp211350_c0 | Cytochrome b5 isoform B | 3.5 | GO:0005789(endoplasmic reticulum membrane);  GO:0016021(integral to membrane);  GO:0005792(microsome);  GO:0020037(heme binding);  GO:0005515(protein binding);  GO:0022900(electron transport chain);  GO:0006810(transport) | ko00520(Amino sugar and nucleotide sugar metabolism) |
|  | comp21909_c0 | Transcription initiation factor TFIID subunit 5 | 3.5 | GO:0015629(actin cytoskeleton);  GO:0005669(transcription factor TFIID complex);  GO:0033276(transcription factor TFTC complex);  GO:0046983(protein dimerization activity);  GO:0003700(sequence-specific DNA binding transcription factor activity);  GO:0044212(transcription regulatory region DNA binding);  GO:0016573(histone acetylation);  GO:0044419(interspecies interaction between organisms);  GO:0006368(transcription elongation from RNA polymerase II promoter);  GO:0006367(transcription initiation from RNA polymerase II promoter);  GO:0016032(viral reproduction) | ko03022(Basal transcription factors) |
|  | comp625687_c0 | Alternative NAD(P)H dehydrogenase 2 | 3.5 |  | ko00190(Oxidative phosphorylation) |
|  | comp45014_c0 | Dehydration-responsive protein RD22 | 3.5 | GO:0009651(response to salt stress) |  |
|  | comp45246_c0 | Transcription factor bHLH1371 | 3.5 | GO:0005634(nucleus);  GO:0003677(DNA binding);  GO:0006355(regulation of transcription, DNA-dependent);  GO:0009739(response to gibberellin stimulus);  GO:0006351(transcription, DNA-dependent) | |
|  | comp1190680_c0 | Proline-rich receptor-like protein kinase PERK2 | 4 | GO:0016021(integral to membrane);  GO:0005886(plasma membrane);  GO:0005524(ATP binding);  GO:0004674(protein serine/threonine kinase activity);  GO:0004872(receptor activity) | |
|  | comp1362776_c0 | Acyl-CoA--sterol O-acyltransferase 1 | 4 | GO:0016021(integral to membrane);  GO:0005515(protein binding);  GO:0016746(transferase activity, transferring acyl groups);  GO:0016127(sterol catabolic process);  GO:0034434(sterol esterification) | |
|  | comp41533_c0 | Probable phenylalanine--tRNA ligase alpha subunit | 4 | GO:0005829(cytosol);  GO:0005524(ATP binding);  GO:0004826(phenylalanine-tRNA ligase activity);  GO:0000049(tRNA binding);  GO:0006432(phenylalanyl-tRNA aminoacylation) | ko00970(Aminoacyl-tRNA biosynthesis) |
|  | comp38455_c0 | Cyanidin-3-O-glucoside 2-O-glucuronosyltransferase | 4 | GO:0005737(cytoplasm);  GO:0016758(transferase activity, transferring hexosyl groups) | |
|  | comp51546_c0 | Aldehyde dehydrogenase family 3 member H1 | 4 | GO:0005783(endoplasmic reticulum);  GO:0016020(membrane);  GO:0009506(plasmodesma);  GO:0005773(vacuole);  GO:0004029(aldehyde dehydrogenase );  GO:0004030(aldehyde dehydrogenase [NAD);  GO:0006081(cellular aldehyde metabolic process);  GO:0009737(response to abscisic acid stimulus);  GO:0009269(response to desiccation);  GO:0009651(response to salt stress) | ko00010(Glycolysis / Gluconeogenesis);  ko00053(Ascorbate and aldarate metabolism);  ko00071(Fatty acid metabolism);  ko00280(Valine,ko leucine and isoleucine degradation);  ko00310(Lysine degradation);  ko00330(Arginine and proline metabolism);  ko00340(Histidine metabolism);  ko00380(Tryptophan metabolism);  ko00410(beta-Alanine metabolism);  ko00561(Glycerolipid metabolism);  ko00620(Pyruvate metabolism);  ko00631(1,ko2-Dichloroethane degradation);  ko00640(Propanoate metabolism);  ko00641(3-Chloroacrylic acid degradation);  ko00650(Butanoate metabolism);  ko00903(Limonene and pinene degradation) |
|  | comp221180_c0 | Pathogenesis-related genes transcriptional activator PTI5 | 4 | GO:0005634(nucleus);  GO:0003677(DNA binding);  GO:0003700(sequence-specific DNA binding transcription factor activity);  GO:0006952(defense response) | |
|  | comp58312_c0 | Probable methyltransferase PMT23 | 4 | GO:0000139(Golgi membrane);  GO:0016021(integral to membrane);  GO:0008168(methyltransferase activity) | |
|  | comp53766_c0 | Anthocyanidin 3-O-glucosyltransferase 2 | 4 |  | ko00944(Flavone and flavonol biosynthesis) |
|  | comp16063_c0 | Probable lysine-specific demethylase JMJ14 | 4 | GO:0005654(nucleoplasm);  GO:0003677(DNA binding);  GO:0032453(histone demethylase activity );  GO:0016702(oxidoreductase activity);  GO:0010216(maintenance of DNA methylation);  GO:0009910(negative regulation of flower development);  GO:0048573(photoperiodism, flowering);  GO:0006355(regulation of transcription, DNA-dependent);  GO:0006351(transcription, DNA-dependent) | |
|  | comp19230_c0 | U2 small nuclear ribonucleoprotein auxiliary factor 35 kDa subunit-related protein 2 | 4 | GO:0005689(U12-type spliceosomal complex);  GO:0000166(nucleotide binding);  GO:0030628(pre-mRNA 3'-splice site binding);  GO:0008270(zinc ion binding);  GO:0000245(spliceosome assembly) | |
|  | comp57520_c0 | Protein transport protein Sec24-like | 4 | GO:0030127(COPII vesicle coat);  GO:0005789(endoplasmic reticulum membrane);  GO:0033116(endoplasmic reticulum-Golgi intermediate compartment membrane);  GO:0000139(Golgi membrane);  GO:0008270(zinc ion binding);  GO:0080119(ER body organization);  GO:0006888(ER to Golgi vesicle-mediated transport);  GO:0006886(intracellular protein transport) | |
|  | comp25434_c0 | Beta-adaptin-like protein A | 4 | GO:0030131(clathrin adaptor complex);  GO:0030665(clathrin coated vesicle membrane);  GO:0005794(Golgi apparatus);  GO:0043424(protein histidine kinase binding);  GO:0008565(protein transporter activity);  GO:0006897(endocytosis);  GO:0006886(intracellular protein transport) | ko04142(Lysosome) |
|  | comp55754_c0 | Pentatricopeptide repeat-containing protein | 4 | GO:0005739(mitochondrion) |  |
|  | comp60239_c1 | NADH-ubiquinone oxidoreductase chain 5 | 4 | GO:0016021(integral to membrane);  GO:0005743(mitochondrial inner membrane);  GO:0070469(respiratory chain);  GO:0008137(NADH dehydrogenase );  GO:0042773(ATP synthesis coupled electron transport);  GO:0006810(transport) | ko00190(Oxidative phosphorylation);  ko05012(Parkinson's disease) |
|  | comp51324_c0 | Acylphosphatase | 4 | GO:0003998(acylphosphatase activity) | ko00620(Pyruvate metabolism);  ko00632(Benzoate degradation via CoA ligation) |
|  | comp39094_c0 | Lon protease homolog 2, peroxisomal | 4 | GO:0005782(peroxisomal matrix);  GO:0005524(ATP binding);  GO:0004176(ATP-dependent peptidase activity);  GO:0004252(serine-type endopeptidase activity);  GO:0006508(proteolysis) | ko04112(Cell cycle - Caulobacter) |
|  | comp47657_c0 | Putative E3 ubiquitin-protein ligase RING1a | 4 | GO:0035102(PRC1 complex);  GO:0016874(ligase activity);  GO:0005515(protein binding);  GO:0008270(zinc ion binding);  GO:0001709(cell fate determination);  GO:0016568(chromatin modification);  GO:0010076(maintenance of floral meristem identity);  GO:0010077(maintenance of inflorescence meristem identity);  GO:0010492(maintenance of shoot apical meristem identity);  GO:0045814(negative regulation of gene expression, epigenetic);  GO:0045892(negative regulation of transcription, DNA-dependent);  GO:0006351(transcription, DNA-dependent) | |
|  | comp28033_c0 | Heme oxygenase 1 | 4 | GO:0009507(chloroplast);  GO:0020037(heme binding);  GO:0004392(heme oxygenase );  GO:0010019(chloroplast-nucleus signaling pathway);  GO:0006788(heme oxidation);  GO:0015979(photosynthesis);  GO:0010024(phytochromobilin biosynthetic process) | ko00860(Porphyrin and chlorophyll metabolism) |
|  | comp46000_c0 | Formin-like protein 6 | 4 | GO:0005618(cell wall);  GO:0016021(integral to membrane);  GO:0005730(nucleolus);  GO:0009524(phragmoplast);  GO:0005886(plasma membrane);  GO:0005819(spindle);  GO:0003779(actin binding);  GO:0030036(actin cytoskeleton organization) | |
| mul-miR477-5p | comp49609_c0 | UDP-glycosyltransferase 89A2 | 3 | GO:0035251(UDP-glucosyltransferase activity) | ko00940(Phenylpropanoid biosynthesis) |
|  | comp58911_c1 | Retrovirus-related Pol polyprotein from transposon 17.6 | 3.5 | GO:0004190(aspartic-type endopeptidase activity);  GO:0003677(DNA binding);  GO:0004519(endonuclease activity);  GO:0003723(RNA binding);  GO:0003964(RNA-directed DNA polymerase activity);  GO:0015074(DNA integration);  GO:0006278(RNA-dependent DNA replication) | |
|  | comp26454_c0 | ABC transporter B family member 19 | 4 | GO:0016021(integral to membrane);  GO:0005886(plasma membrane);  GO:0005524(ATP binding);  GO:0042626(ATPase activity, coupled to transmembrane movement of substances);  GO:0010329(auxin efflux transmembrane transporter activity);  GO:0005515(protein binding);  GO:0010541(acropetal auxin transport);  GO:0043481(anthocyanin accumulation in tissues in response to UV light);  GO:0010540(basipetal auxin transport);  GO:0048527(lateral root development);  GO:0009640(photomorphogenesis);  GO:0009958(positive gravitropism);  GO:0008361(regulation of cell size);  GO:0009637(response to blue light);  GO:0010218(response to far red light);  GO:0048443(stamen development) | ko02010(ABC transporters) |
|  | comp36865_c0 | 50S ribosomal protein L12 | 4 | GO:0009507(chloroplast);  GO:0005840(ribosome);  GO:0003735(structural constituent of ribosome);  GO:0006412(translation) | ko03010(Ribosome) |
| mul-miR477a-5p | comp60401_c0 | Putative disease resistance RPP13-like protein 1 | 2.5 | GO:0005524(ATP binding);  GO:0005515(protein binding);  GO:0006915(apoptosis);  GO:0006952(defense response) | |
|  | comp54542_c0 | ATP-dependent helicase BRM | 3 | GO:0016585(chromatin remodeling complex);  GO:0005829(cytosol);  GO:0005524(ATP binding);  GO:0003677(DNA binding);  GO:0004386(helicase activity);  GO:0043044(ATP-dependent chromatin remodeling);  GO:0010199(organ boundary specification between lateral organs and the meristem);  GO:0040029(regulation of gene expression, epigenetic);  GO:0006355(regulation of transcription, DNA-dependent);  GO:0006351(transcription, DNA-dependent) | |
|  | comp37049_c0 | Mediator of RNA polymerase II transcription subunit 14 | 3 |  |  |
|  | comp389874_c0 | Photosystem I reaction center subunit XI | 3.5 | GO:0009535(chloroplast thylakoid membrane);  GO:0016021(integral to membrane);  GO:0009538(photosystem I reaction center);  GO:0015979(photosynthesis) | ko00195(Photosynthesis) |
|  | comp12888_c0 | Bidirectional sugar transporter SWEET10 | 3.5 | GO:0005887(integral to plasma membrane);  GO:0051119(sugar transmembrane transporter activity) | |
|  | comp14576_c0 | Cyclic nucleotide-gated ion channel 1 | 3.5 | GO:0016021(integral to membrane);  GO:0005886(plasma membrane);  GO:0005516(calmodulin binding);  GO:0030552(cAMP binding);  GO:0030553(cGMP binding);  GO:0005221(intracellular cyclic nucleotide activated cation channel activity);  GO:0006816(calcium ion transport);  GO:0006813(potassium ion transport) | ko04626(Plant-pathogen interaction) |
|  | comp15782_c0 | Retrovirus-related Pol polyprotein from transposon TNT 1-94 | 4 | GO:0004190(aspartic-type endopeptidase activity);  GO:0003677(DNA binding);  GO:0004519(endonuclease activity);  GO:0003964(RNA-directed DNA polymerase activity);  GO:0008270(zinc ion binding);  GO:0015074(DNA integration) | |
|  | comp49609_c0 | UDP-glycosyltransferase 89A2 | 4 | GO:0035251(UDP-glucosyltransferase activity) | ko00940(Phenylpropanoid biosynthesis) |
|  | comp59620_c0 | Serine/threonine-protein kinase AtPK2/AtPK19 | 4 | GO:0005634(nucleus);  GO:0005524(ATP binding);  GO:0004674(protein serine/threonine kinase activity);  GO:0045727(positive regulation of translation);  GO:0009409(response to cold);  GO:0009408(response to heat);  GO:0009651(response to salt stress) | |
|  | comp32061_c0 | NAC domain-containing protein 8 | 4 | GO:0005634(nucleus);GO:0003677(DNA binding);  GO:0000077(DNA damage checkpoint);  GO:0040020(regulation of meiosis);  GO:0006355(regulation of transcription, DNA-dependent);  GO:0010332(response to gamma radiation);  GO:0006351(transcription, DNA-dependent) | |
| mul-miR482c-3p | comp48821_c0 | Putative disease resistance protein | 3.5 | GO:0009506(plasmodesma);  GO:0005524(ATP binding);  GO:0006915(apoptosis);  GO:0006952(defense response) | |
|  | comp52238_c0 | Putative disease resistance protein | 3.5 | GO:0009506(plasmodesma);  GO:0005524(ATP binding);  GO:0006915(apoptosis);  GO:0006952(defense response) | |
|  | comp52680_c0 | Putative disease resistance protein RGA3 | 4 | GO:0005524(ATP binding);  GO:0006915(apoptosis);  GO:0006952(defense response) | |
|  | comp57508_c0 | Putative disease resistance protein | 4 | GO:0009506(plasmodesma);  GO:0005524(ATP binding);  GO:0006915(apoptosis);  GO:0006952(defense response) | |
| mul-miR482c-3p-1 | comp1371831_c0 | Putative disease resistance protein RGA1? 3 | 3 | GO:0005524(ATP binding);  GO:0006915(apoptosis);  GO:0006952(defense response) | |
|  | comp1102983_c0 | Aldehyde dehydrogenase family 2 member C4 | 4 | GO:0005829(cytosol);  GO:0004029(aldehyde dehydrogenase );  GO:0050269(coniferyl-aldehyde dehydrogenase activity);  GO:0009699(phenylpropanoid biosynthetic process) | ko00940(Phenylpropanoid biosynthesis) |
|  | comp48015_c0 | Disease resistance protein RGA2 | 4 | GO:0005524(ATP binding);  GO:0006915(apoptosis);  GO:0006952(defense response) | |
|  | comp59976_c0 | Putative disease resistance RPP13-like protein 1 | 4 | GO:0005524(ATP binding);  GO:0005515(protein binding);  GO:0006915(apoptosis);  GO:0006952(defense response) | |
|  | comp46685_c0 | Pentatricopeptide repeat-containing protein | 4 | GO:0009507(chloroplast);  GO:0005488(binding);  GO:0031425(chloroplast RNA processing) | |
| mul-miR5077 | comp19453_c0 | UDP-galactose transporter 2 | 2 | GO:0016021(integral to membrane);  GO:0005457(GDP-fucose transmembrane transporter activity);  GO:0005459(UDP-galactose transmembrane transporter activity);  GO:0005460(UDP-glucose transmembrane transporter activity);  GO:0009624(response to nematode) | |
|  | comp43662_c0 | Photosystem I reaction center subunit II | 2.5 | GO:0009535(chloroplast thylakoid membrane);  GO:0009538(photosystem I reaction center);  GO:0015979(photosynthesis) | ko00195(Photosynthesis) |
|  | comp60365_c1 | NADH-ubiquinone oxidoreductase chain 5 | 3 | GO:0016021(integral to membrane);  GO:0005743(mitochondrial inner membrane);  GO:0070469(respiratory chain);  GO:0008137(NADH dehydrogenase );  GO:0042773(ATP synthesis coupled electron transport);  GO:0006810(transport) | ko00190(Oxidative phosphorylation);  ko05012(Parkinson's disease) |
|  | comp50679_c0 | UDP-glycosyltransferase 86A1 | 3 | GO:0016758(transferase activity, transferring hexosyl groups) | |
|  | comp298172_c0 | ATP sulfurylase 2 | 3.5 | GO:0009570(chloroplast stroma);  GO:0005829(cytosol);  GO:0005524(ATP binding);  GO:0004781(sulfate adenylyltransferase );  GO:0009970(cellular response to sulfate starvation);  GO:0000103(sulfate assimilation) | ko00230(Purine metabolism);  ko00450(Selenoamino acid metabolism);  ko00920(Sulfur metabolism) |
|  | comp9030_c0 | Anthranilate N-benzoyltransferase protein 2 | 3.5 | GO:0047672(anthranilate N-benzoyltransferase activity);  GO:0009813(flavonoid biosynthetic process) | |
|  | comp47341_c0 | Proline-rich receptor-like protein kinase PERK2 | 4 | GO:0016021(integral to membrane);  GO:0005886(plasma membrane);  GO:0005524(ATP binding);  GO:0004674(protein serine/threonine kinase activity);  GO:0004872(receptor activity) | |
|  | comp35630_c0 | 50S ribosomal protein L15 | 4 | GO:0009570(chloroplast stroma);  GO:0000311(plastid large ribosomal subunit);  GO:0003735(structural constituent of ribosome);  GO:0006412(translation) | ko03010(Ribosome) |
|  | comp35421_c0 | Transcription factor bHLH30 | 4 | GO:0005634(nucleus);  GO:0003677(DNA binding);  GO:0006355(regulation of transcription, DNA-dependent);  GO:0006351(transcription, DNA-dependent) | |
|  | comp50727_c0 | Kinesin-related protein 13 | 4 | GO:0005813(centrosome);  GO:0005874(microtubule);  GO:0005524(ATP binding);  GO:0003777(microtubule motor activity);  GO:0051301(cell division);  GO:0007018(microtubule-based movement);  GO:0007067(mitosis);  GO:0051231(spindle elongation);  GO:0043146(spindle stabilization);  GO:0006810(transport) | |
|  | comp14910_c0 | UDP-glycosyltransferase 86A1 | 4 | GO:0016758(transferase activity, transferring hexosyl groups) | |
|  | comp58882_c0 | Antiviral helicase SKI2 | 4 | GO:0055087(Ski complex);  GO:0005524(ATP binding);  GO:0008026(ATP-dependent helicase activity);  GO:0003677(DNA binding);  GO:0005515(protein binding);  GO:0003723(RNA binding);  GO:0070478(nuclear-transcribed mRNA catabolic process, 3'-5' exonucleolytic nonsense-mediated decay);  GO:0070481(nuclear-transcribed mRNA catabolic process, non-stop decay);  GO:0006417(regulation of translation);  GO:0009615(response to virus) | ko00230(Purine metabolism) |
| mul-miR5225-3p | comp9071_c0 | DNA helicase INO80 | 4 | GO:0005634(nucleus);  GO:0005524(ATP binding);  GO:0003677(DNA binding);  GO:0004386(helicase activity);  GO:0016568(chromatin modification);  GO:0006281(DNA repair);  GO:0045739(positive regulation of DNA repair);  GO:0006355(regulation of transcription, DNA-dependent);  GO:0016444(somatic cell DNA recombination);  GO:0006351(transcription, DNA-dependent) | |
| mul-miR530a | comp47327_c0 | Putative zinc transporter | 2.5 | GO:0016021(integral to membrane);  GO:0046873(metal ion transmembrane transporter activity);  GO:0006829(zinc ion transport) | |
|  | comp54885_c0 | Copia protein | 3 |  |  |
|  | comp28328_c0 | Potassium transporter 2 | 3.5 | GO:0016021(integral to membrane);  GO:0005886(plasma membrane);  GO:0015079(potassium ion transmembrane transporter activity) | |
|  | comp41927_c0 | NAC domain-containing protein 100 | 3.5 | GO:0005634(nucleus);  GO:0003677(DNA binding);  GO:0006355(regulation of transcription, DNA-dependent);  GO:0006351(transcription, DNA-dependent) | |
|  | comp59750_c1 | GPI inositol-deacylase | 4 | GO:0016021(integral to membrane);  GO:0031227(intrinsic to endoplasmic reticulum membrane);  GO:0016788(hydrolase activity, acting on ester bonds);  GO:0006505(GPI anchor metabolic process);  GO:0006886(intracellular protein transport) | ko00563(Glycosylphosphatidylinositol) |
|  | comp12841_c0 | Translationally-controlled tumor protein homolog | 4 | GO:0005737(cytoplasm) |  |
|  | comp39756_c0 | Ubiquitin-like modifier-activating enzyme 5 | 4 | GO:0005829(cytosol);  GO:0005524(ATP binding);  GO:0048037(cofactor binding);  GO:0046872(metal ion binding);  GO:0016616(oxidoreductase activity, acting on the CH-OH group of donors) | |
| mul-miR827 | comp50344_c0 | SPX domain-containing membrane protein | 2 | GO:0016021(integral to membrane);  GO:0055085(transmembrane transport) | |
| mul-miR828b-3p | comp42435_c0 | Carbon catabolite repressor protein 4 homolog 1 | 3.5 | GO:0005829(cytosol);  GO:0005634(nucleus);  GO:0046872(metal ion binding);  GO:0004535(poly);  GO:0003723(RNA binding);  GO:0006355(regulation of transcription, DNA-dependent);  GO:0006351(transcription, DNA-dependent) | |
|  | comp55756_c0 | DNA repair helicase XPB1 | 3.5 | GO:0005737(cytoplasm);  GO:0005634(nucleus);  GO:0005524(ATP binding);  GO:0004003(ATP-dependent DNA helicase activity);  GO:0003677(DNA binding);  GO:0006289(nucleotide-excision repair);  GO:0006355(regulation of transcription, DNA-dependent);  GO:0009411(response to UV);  GO:0006351(transcription, DNA-dependent) | ko03420(Nucleotide excision repair) |
|  | comp53749_c0 | F-box protein | 4 |  |  |
|  | comp46265_c0 | N(6)-adenine-specific DNA methyltransferase 2 | 4 | GO:0008168(methyltransferase activity);  GO:0003676(nucleic acid binding) | |
|  | comp45423_c0 | EIN3-binding F-box protein 1 | 4 | GO:0005634(nucleus);  GO:0005515(protein binding);  GO:0009873(ethylene mediated signaling pathway);  GO:0010105(negative regulation of ethylene mediated signaling pathway);  GO:0006511(ubiquitin-dependent protein catabolic process) | |
| mul-miR858b | comp31103_c0 | Myb-related protein P | 3.5 | GO:0005634(nucleus);  GO:0003677(DNA binding);  GO:0006355(regulation of transcription, DNA-dependent);  GO:0006351(transcription, DNA-dependent) | |
|  | comp43581_c0 | 50S ribosomal protein 5 | 4 | GO:0009507(chloroplast);  GO:0005840(ribosome) |  |
| mul-miR894 | comp686928_c0 | Phospholipase D delta | 2.5 | GO:0005886(plasma membrane);  GO:0009506(plasmodesma);  GO:0005773(vacuole);  GO:0005509(calcium ion binding);  GO:0070290(NAPE-specific phospholipase D activity);  GO:0004630(phospholipase D activity);  GO:0016042(lipid catabolic process);  GO:0046473(phosphatidic acid metabolic process);  GO:0046470(phosphatidylcholine metabolic process);  GO:0012501(programmed cell death);  GO:0009409(response to cold) | ko00564(Glycerophospholipid metabolism);  ko00565(Ether lipid metabolism);  ko04144(Endocytosis);  ko04666(Fc gamma R-mediated phagocytosis);  ko04912(GnRH signaling pathway) |
|  | comp24839_c0 | Putative glycerol-3-phosphate transporter 1 | 3 | GO:0016021(integral to membrane);  GO:0008643(carbohydrate transport);  GO:0055085(transmembrane transport) | ko02020(Two-component system) |
|  | comp41654_c0 | Nucleolar GTP-binding protein 2 | 3 | GO:0005730(nucleolus);  GO:0005525(GTP binding);  GO:0003924(GTPase activity);  GO:0042254(ribosome biogenesis) | |
|  | comp53683_c0 | Protein phosphatase 2C 16 | 3.5 | GO:0005737(cytoplasm);  GO:0005634(nucleus);  GO:0008287(protein serine/threonine phosphatase complex);  GO:0046872(metal ion binding);  GO:0005515(protein binding);  GO:0004722(protein serine/threonine phosphatase activity);  GO:0009738(abscisic acid mediated signaling pathway);  GO:0006470(protein dephosphorylation) | |
|  | comp57012_c0 | Protein RAFTIN 1B | 3.5 | GO:0043668(exine);  GO:0009555(pollen development) |  |
|  | comp37031_c0 | Copper transporter 3 | 4 | GO:0016021(integral to membrane);  GO:0005375(copper ion transmembrane transporter activity) | |
|  | comp19453_c0 | UDP-galactose transporter 2 | 4 | GO:0016021(integral to membrane);  GO:0005457(GDP-fucose transmembrane transporter activity);  GO:0005459(UDP-galactose transmembrane transporter activity);  GO:0005460(UDP-glucose transmembrane transporter activity);  GO:0009624(response to nematode) | |
|  | comp16431_c0 | ATP-dependent zinc metalloprotease FTSH 2 | 4 | GO:0009535(chloroplast thylakoid membrane);  GO:0016021(integral to membrane);  GO:0005524(ATP binding);  GO:0046872(metal ion binding);  GO:0004222(metalloendopeptidase activity);  GO:0017111(nucleoside-triphosphatase activity);  GO:0030163(protein catabolic process);  GO:0006508(proteolysis) | |
|  | comp56555_c0 | Elongation factor G | 4 | GO:0005739(mitochondrion);  GO:0005524(ATP binding);  GO:0005525(GTP binding);  GO:0003924(GTPase activity);  GO:0003746(translation elongation factor activity);  GO:0046686(response to cadmium ion) | |
|  | comp298172_c0 | ATP sulfurylase 2 | 4 | GO:0009570(chloroplast stroma);  GO:0005829(cytosol);  GO:0005524(ATP binding);  GO:0004781(sulfate adenylyltransferase );  GO:0009970(cellular response to sulfate starvation);  GO:0000103(sulfate assimilation) | ko00230(Purine metabolism);  ko00450(Selenoamino acid metabolism);  ko00920(Sulfur metabolism) |
|  | comp38889_c0 | Primary amine oxidase (Fragment) | 4 | GO:0052595(aliphatic-amine oxidase activity);  GO:0052594(aminoacetone:oxygen oxidoreductase);  GO:0005507(copper ion binding);  GO:0052596(phenethylamine:oxygen oxidoreductase );  GO:0008131(primary amine oxidase activity);  GO:0048038(quinone binding);  GO:0052593(tryptamine:oxygen oxidoreductase );  GO:0009308(amine metabolic process) | ko00260(Glycine,ko serine and threonine metabolism);  ko00350(Tyrosine metabolism);  ko00360(Phenylalanine metabolism);  ko00410(beta-Alanine metabolism);  ko00950(Isoquinoline alkaloid biosynthesis);  ko00960(Tropane,ko piperidine and pyridine alkaloid biosynthesis) |
|  | comp57598_c0 | Heterogeneous nuclear ribonucleoprotein 1 | 4 |  | ko04320(Dorso-ventral axis formation) |
|  | comp42352_c0 | Probable beta-1,4-xylosyltransferase IRX14 | 4 | GO:0000139(Golgi membrane);  GO:0016021(integral to membrane);  GO:0015018(galactosylgalactosylxylosylprotein 3-beta-glucuronosyltransferase activity);  GO:0042285(xylosyltransferase activity);  GO:0007047(cellular cell wall organization);  GO:0010154(fruit development);  GO:0010417(glucuronoxylan biosynthetic process);  GO:0048367(shoot development);  GO:0010051(xylem and phloem pattern formation) | |
| mul-miRn01 | comp41353_c0 | Cytochrome b-c1 complex subunit 9 | 3.5 | GO:0005743(mitochondrial inner membrane);  GO:0070469(respiratory chain);  GO:0008121(ubiquinol-cytochrome-c reductase activity);  GO:0006122(mitochondrial electron transport, ubiquinol to cytochrome c) | ko00190(Oxidative phosphorylation);  ko04260(Cardiac muscle contraction);  ko05010(Alzheimer's disease);  ko05012(Parkinson's disease);  ko05016(Huntington's disease) |
| mul-miRn08 | comp52399_c0 | Probable xyloglucan endotransglucosylase/hydrolase protein 30 | 4 | GO:0048046(apoplast);  GO:0005618(cell wall);  GO:0004553(hydrolase activity, hydrolyzing O-glycosyl compounds);  GO:0016762(xyloglucan:xyloglucosyl transferase activity);  GO:0007047(cellular cell wall organization);  GO:0006073(cellular glucan metabolic process) | |
|  | comp58396_c0 | Heat shock 70 kDa protein 17 | 4 |  |  |
| mul-miRn09 | comp9605_c0 | Protein yippee-like | 4 |  |  |
|  | comp11989_c0 | Rop guanine nucleotide exchange factor 1 | 4 | GO:0016324(apical plasma membrane);  GO:0005089(Rho guanyl-nucleotide exchange factor activity);  GO:0009860(pollen tube growth) | |
|  | comp59191_c0 | Cyclin-dependent kinase 11A | 4 |  |  |
| mul-miRn14 | comp55271_c0 | Protein disulfide-isomerase | 0 | GO:0005788(endoplasmic reticulum lumen);  GO:0009055(electron carrier activity);  GO:0003756(protein disulfide isomerase activity);  GO:0015035(protein disulfide oxidoreductase activity);  GO:0045454(cell redox homeostasis);  GO:0006662(glycerol ether metabolic process) | |
| mul-miRn15 | comp53389_c1 | Sucrose-binding protein | 0 | GO:0016020(membrane);  GO:0045735(nutrient reservoir activity);  GO:0008643(carbohydrate transport) | |
|  | comp44630_c0 | ATP-dependent zinc metalloprotease FTSH 2 | 4 | GO:0009535(chloroplast thylakoid membrane);  GO:0016021(integral to membrane);  GO:0005524(ATP binding);  GO:0046872(metal ion binding);  GO:0004222(metalloendopeptidase activity);  GO:0017111(nucleoside-triphosphatase activity);  GO:0030163(protein catabolic process);  GO:0006508(proteolysis) | |
| mul-miRn16 | comp53389_c1 | Sucrose-binding protein | 0 | GO:0016020(membrane);  GO:0045735(nutrient reservoir activity);  GO:0008643(carbohydrate transport) | |
|  | comp37287_c0 | Probable LRR receptor-like serine/threonine-protein kinase | 3 | GO:0016021(integral to membrane);  GO:0005524(ATP binding);  GO:0004674(protein serine/threonine kinase activity);  GO:0004872(receptor activity) | ko04210(Apoptosis);  ko04620(Toll-like receptor signaling pathway);  ko04722(Neurotrophin signaling pathway);  ko05140(Leishmaniasis);  ko05142(Chagas disease) |
| mul-miRn18 | comp55778_c0 | DEAD-box ATP-dependent RNA helicase 52A | 4 | GO:0005524(ATP binding);  GO:0008026(ATP-dependent helicase activity);  GO:0003723(RNA binding) | ko04622(RIG-I-like receptor signaling pathway) |
|  | comp14105_c0 | Sodium/hydrogen exchanger 2 | 4 | GO:0016021(integral to membrane);  GO:0005774(vacuolar membrane);  GO:0015385(sodium:hydrogen antiporter activity);  GO:0006885(regulation of pH) | |
|  | comp51878_c0 | UNC93-like protein 3 | 4 | GO:0016021(integral to membrane) |  |
| mul-miRn19 | comp45700_c0 | Protein IQ-DOMAIN 31 | 3 | GO:0005829(cytosol);  GO:0005886(plasma membrane) |  |
|  | comp39695_c0 | Histone deacetylase 5 | 3.5 | GO:0005737(cytoplasm);  GO:0005634(nucleus);  GO:0034739(histone deacetylase activity );  GO:0032041(NAD-dependent histone deacetylase activity );  GO:0046969(NAD-dependent histone deacetylase activity );  GO:0046970(NAD-dependent histone deacetylase activity );  GO:0006355(regulation of transcription, DNA-dependent);  GO:0006351(transcription, DNA-dependent) | |
|  | comp48536_c0 | Amidase 1 | 4 |  | ko00330(Arginine and proline metabolism);  ko00360(Phenylalanine metabolism);  ko00380(Tryptophan metabolism);  ko00460(Cyanoamino acid metabolism);  ko00632(Benzoate degradation via CoA ligation);  ko00643(Styrene degradation) |
| mul-miRn23 | comp59262_c0 | Proline-rich receptor-like protein kinase PERK2 | 2 | GO:0016021(integral to membrane);  GO:0005886(plasma membrane);  GO:0005524(ATP binding);  GO:0004674(protein serine/threonine kinase activity);  GO:0004872(receptor activity) | |
|  | comp40360_c0 | Probable fructokinase-1 | 4 | GO:0005829(cytosol);  GO:0005886(plasma membrane);  GO:0005524(ATP binding);  GO:0008865(fructokinase activity);  GO:0004747(ribokinase activity);  GO:0006014(D-ribose metabolic process) | ko00051(Fructose and mannose metabolism);  ko00500(Starch and sucrose metabolism);  ko00520(Amino sugar and nucleotide sugar metabolism) |
| mul-miRn24 | comp59262_c0 | Proline-rich receptor-like protein kinase PERK2 | 0 | GO:0016021(integral to membrane);  GO:0005886(plasma membrane);  GO:0005524(ATP binding);  GO:0004674(protein serine/threonine kinase activity);  GO:0004872(receptor activity) | |
| mul-miRn25 | comp60064_c0 | Cytochrome c oxidase subunit 1 | 3.5 | GO:0016021(integral to membrane);  GO:0005743(mitochondrial inner membrane);  GO:0005886(plasma membrane);  GO:0070469(respiratory chain);  GO:0005507(copper ion binding);  GO:0004129(cytochrome-c oxidase activity);  GO:0009055(electron carrier activity);  GO:0020037(heme binding);  GO:0009060(aerobic respiration);  GO:0022900(electron transport chain) | ko00190(Oxidative phosphorylation);  ko04260(Cardiac muscle contraction);  ko05010(Alzheimer's disease);  ko05012(Parkinson's disease);  ko05016(Huntington's disease) |
| mul-miRn26 | comp57944_c0 | Protein VERNALIZATION INSENSITIVE 3 | 3.5 | GO:0005634(nucleus);  GO:0003677(DNA binding);  GO:0005515(protein binding);  GO:0008270(zinc ion binding);  GO:0006355(regulation of transcription, DNA-dependent);  GO:0001666(response to hypoxia);  GO:0006351(transcription, DNA-dependent);  GO:0010048(vernalization response) | |
| mul-miRn27 | comp59071_c0 | Putative metallophosphoesterase | 4 | GO:0016021(integral to membrane);  GO:0016787(hydrolase activity);  GO:0046872(metal ion binding) | |
| mul-miRn30 | comp22735_c0 | Auxin-responsive protein IAA13 | 3 | GO:0005634(nucleus);  GO:0046983(protein dimerization activity);  GO:0009734(auxin mediated signaling pathway);  GO:0006355(regulation of transcription, DNA-dependent);  GO:0006351(transcription, DNA-dependent) | |
|  | comp38832_c0 | Probable LRR receptor-like serine/threonine-protein kinase | 4 | GO:0016021(integral to membrane);  GO:0005886(plasma membrane);  GO:0005524(ATP binding);  GO:0004674(protein serine/threonine kinase activity);  GO:0004872(receptor activity) | |
|  | comp47913_c0 | Glucan endo-1,3-beta-glucosidase-like protein | 4 | GO:0031225(anchored to membrane);  GO:0005886(plasma membrane);  GO:0009506(plasmodesma) | ko00500(Starch and sucrose metabolism) |
| mul-miRn32 | comp44458_c0 | Ribonuclease 3 | 4 | GO:0033897(ribonuclease T2 activity);  GO:0003723(RNA binding) | |
|  | comp49937_c0 | ATP-citrate synthase beta chain protein 2 | 4 | GO:0009346(citrate lyase complex);  GO:0005829(cytosol);  GO:0005886(plasma membrane);  GO:0005524(ATP binding);  GO:0003878(ATP citrate synthase activity);  GO:0046872(metal ion binding);  GO:0004775(succinate-CoA ligase );  GO:0006085(acetyl-CoA biosynthetic process);  GO:0044262(cellular carbohydrate metabolic process);  GO:0008610(lipid biosynthetic process) | ko00020(Citrate cycle );  ko00720(Reductive carboxylate cycle ) |
| mul-miRn34 | comp11844_c0 | Ribosome production factor 2 homolog | 4 | GO:0005730(nucleolus) |  |
| mul-miRn37 | comp52137_c0 | Actin-depolymerizing factor 2 | 4 | GO:0005622(intracellular);  GO:0003779(actin binding) | ko04360(Axon guidance);  ko04666(Fc gamma R-mediated phagocytosis);  ko04810(Regulation of actin cytoskeleton) |
|  | comp59708_c0 | Brefeldin A-inhibited guanine nucleotide-exchange protein 5 | 4 |  | ko04144(Endocytosis) |
| mul-miRn38 | comp55138_c0 | Serine/threonine-protein phosphatase 4 regulatory subunit 3A | 4 | GO:0005815(microtubule organizing center);  GO:0005634(nucleus);  GO:0005488(binding) | |
| mul-miRn42 | comp46804_c0 | Calcium-dependent protein kinase 32 | 3 | GO:0005737(cytoplasm);  GO:0005634(nucleus);  GO:0005886(plasma membrane);  GO:0009506(plasmodesma);  GO:0005524(ATP binding);  GO:0005509(calcium ion binding);  GO:0004698(calcium-dependent protein kinase C activity);  GO:0005515(protein binding);  GO:0009738(abscisic acid mediated signaling pathway);  GO:0009651(response to salt stress) | |
|  | comp46565_c0 | Pentatricopeptide repeat-containing protein | 4 | GO:0005739(mitochondrion);  GO:0005488(binding) |  |
| mul-miRn43 | comp60289_c0 | Chaperone protein ClpB3 | 3.5 | GO:0005739(mitochondrion);  GO:0005524(ATP binding);  GO:0017111(nucleoside-triphosphatase activity) | |
| mul-miRn52 | comp59557_c0 | NAD(P)-specific glutamate dehydrogenase | 0 | GO:0005488(binding);  GO:0004353(glutamate dehydrogenase [NAD);  GO:0006520(cellular amino acid metabolic process) | ko00250(Alanine,ko aspartate and glutamate metabolism);  ko00330(Arginine and proline metabolism);  ko00910(Nitrogen metabolism) |
|  | comp50812_c0 | Shikimate O-hydroxycinnamoyltransferase | 3.5 | GO:0005829(cytosol);  GO:0016787(hydrolase activity);  GO:0047205(quinate O-hydroxycinnamoyltransferase activity);  GO:0047172(shikimate O-hydroxycinnamoyltransferase activity);  GO:0010252(auxin homeostasis);  GO:0009809(lignin biosynthetic process);  GO:0009963(positive regulation of flavonoid biosynthetic process) | |
|  | comp41559_c0 | Laccase-15 | 3.5 | GO:0048046(apoplast);  GO:0005507(copper ion binding);  GO:0052716(hydroquinone:oxygen oxidoreductase activity);  GO:0008471(laccase activity);  GO:0009809(lignin biosynthetic process);  GO:0046274(lignin catabolic process);  GO:0010023(proanthocyanidin biosynthetic process);  GO:0046688(response to copper ion) | ko00910(Nitrogen metabolism) |
|  | comp45213_c0 | Bifunctional aspartate aminotransferase and glutamate/aspartate-prephenate aminotransferase | 4 |  | ko00250(Alanine,ko aspartate and glutamate metabolism);  ko00270(Cysteine and methionine metabolism);  ko00330(Arginine and proline metabolism);  ko00350(Tyrosine metabolism);  ko00360(Phenylalanine metabolism);  ko00400(Phenylalanine,ko tyrosine and tryptophan biosynthesis);  ko00401(Novobiocin biosynthesis);  ko00710(Carbon fixation in photosynthetic organisms);  ko00950(Isoquinoline alkaloid biosynthesis);  ko00960(Tropane,ko piperidine and pyridine alkaloid biosynthesis) |
| mul-miRn53 | comp59557_c0 | NAD(P)-specific glutamate dehydrogenase | 1 | GO:0005488(binding);  GO:0004353(glutamate dehydrogenase [NAD);  GO:0006520(cellular amino acid metabolic process) | ko00250(Alanine,ko aspartate and glutamate metabolism);  ko00330(Arginine and proline metabolism);  ko00910(Nitrogen metabolism) |
|  | comp46088_c0 | G-type lectin S-receptor-like serine/threonine-protein kinase | 4 | GO:0016021(integral to membrane);  GO:0044459(plasma membrane part);  GO:0005524(ATP binding);  GO:0005516(calmodulin binding);  GO:0004674(protein serine/threonine kinase activity);  GO:0004872(receptor activity);  GO:0005529(sugar binding);  GO:0048544(recognition of pollen) | |
|  | comp56367_c0 | Galacturonosyltransferase 8 | 4 | GO:0000139(Golgi membrane);  GO:0016021(integral to membrane);  GO:0005739(mitochondrion);  GO:0016757(transferase activity, transferring glycosyl groups);  GO:0007155(cell adhesion);  GO:0007047(cellular cell wall organization);  GO:0010289(homogalacturonan biosynthetic process) | ko00051(Fructose and mannose metabolism) |
| mul-miRn55 | comp25180_c0 | Protein NAP1 | 4 | GO:0031209(SCAR complex);  GO:0005515(protein binding);  GO:0045010(actin nucleation);  GO:0009825(multidimensional cell growth);  GO:0045893(positive regulation of transcription, DNA-dependent);  GO:0010090(trichome morphogenesis) | ko04810(Regulation of actin cytoskeleton) |
|  | comp56045_c0 | Polyphenol oxidase | 4 | GO:0009543(chloroplast thylakoid lumen);  GO:0004097(catechol oxidase activity);  GO:0046872(metal ion binding) | |
| mul-miRn56 | comp55537_c0 | Metacaspase-1 | 3.5 | GO:0004197(cysteine-type endopeptidase activity);  GO:0006952(defense response);  GO:0006917(induction of apoptosis);  GO:0006508(proteolysis) | |
|  | comp50125_c0 | Serine/threonine-protein kinase Nek6 | 4 | GO:0005524(ATP binding);  GO:0004674(protein serine/threonine kinase activity) | |
| mul-miRn58 | comp53731_c0 | GMP synthase [glutamine-hydrolyzing] | 4 | GO:0005524(ATP binding);  GO:0003922(GMP synthase );  GO:0006541(glutamine metabolic process);  GO:0006177(GMP biosynthetic process) | ko00230(Purine metabolism);  ko00983(Drug metabolism - other enzymes) |
| mul-miRn60 | comp51369_c0 | 1-acylglycerol-3-phosphate O-acyltransferase ABHD5 | 2.5 | GO:0005829(cytosol);  GO:0005811(lipid particle);  GO:0003841(1-acylglycerol-3-phosphate O-acyltransferase activity);  GO:0030154(cell differentiation);  GO:0006631(fatty acid metabolic process);  GO:0010891(negative regulation of sequestering of triglyceride);  GO:0006654(phosphatidic acid biosynthetic process);  GO:0010898(positive regulation of triglyceride catabolic process) | |
| mul-miRn71 | comp49775_c0 | Peptidyl-tRNA hydrolase 2 | 3 | GO:0005739(mitochondrion);  GO:0004045(aminoacyl-tRNA hydrolase activity) | |
| mul-miRn73 | comp59348_c1 | Nudix hydrolase 2 | 1 | GO:0047631(ADP-ribose diphosphatase activity);  GO:0046872(metal ion binding);  GO:0051287(NAD binding);  GO:0000210(NAD+ diphosphatase activity);  GO:0006979(response to oxidative stress) | |
|  | comp58150_c0 | ABC transporter D family member 2 | 2.5 | GO:0009507(chloroplast);  GO:0016021(integral to membrane);  GO:0005524(ATP binding);  GO:0042626(ATPase activity, coupled to transmembrane movement of substances) | ko02010(ABC transporters) |
|  | comp50144_c0 | Acid phosphatase 1 | 3 | GO:0003993(acid phosphatase activity) | ko03010(Ribosome) |
|  | comp52587_c0 | Auxin response factor 1 | 4 | GO:0005634(nucleus);  GO:0003677(DNA binding);  GO:0046983(protein dimerization activity);  GO:0009734(auxin mediated signaling pathway);  GO:0010150(leaf senescence);  GO:0045892(negative regulation of transcription, DNA-dependent);  GO:0006351(transcription, DNA-dependent) | |
| mul-miRn74 | comp55138_c0 | Serine/threonine-protein phosphatase 4 regulatory subunit 3A | 0 | GO:0005815(microtubule organizing center);  GO:0005634(nucleus);  GO:0005488(binding) | |
| mul-miRn77 | comp28478_c0 | Glyceraldehyde-3-phosphate dehydrogenase | 0 | GO:0005737(cytoplasm);  GO:0004365(glyceraldehyde-3-phosphate dehydrogenase );  GO:0051287(NAD binding);  GO:0006096(glycolysis) | ko00010(Glycolysis / Gluconeogenesis);  ko05010(Alzheimer's disease) |
|  | comp51845_c0 | E3 ubiquitin-protein ligase UPL3 | 4 | GO:0005622(intracellular);  GO:0005886(plasma membrane);  GO:0016881(acid-amino acid ligase activity);  GO:0005488(binding);GO:0042023(DNA endoreduplication);  GO:0006464(protein modification process);  GO:0010091(trichome branching) | ko04120(Ubiquitin mediated proteolysis) |
| mul-miRn78 | comp59348_c1 | Nudix hydrolase 2 | 2.5 | GO:0047631(ADP-ribose diphosphatase activity);  GO:0046872(metal ion binding);  GO:0051287(NAD binding);  GO:0000210(NAD+ diphosphatase activity);  GO:0006979(response to oxidative stress) | |
|  | comp58150_c0 | ABC transporter D family member 2 | 3.5 | GO:0009507(chloroplast);  GO:0016021(integral to membrane);  GO:0005524(ATP binding);  GO:0042626(ATPase activity, coupled to transmembrane movement of substances) | ko02010(ABC transporters) |
| mul-miRn79 | comp38478_c0 | Myosin-J heavy chain | 4 | GO:0042641(actomyosin);  GO:0000331(contractile vacuole);  GO:0016459(myosin complex);  GO:0051015(actin filament binding);  GO:0030898(actin-dependent ATPase activity);  GO:0043531(ADP binding);  GO:0005524(ATP binding);  GO:0005516(calmodulin binding);  GO:0000146(microfilament motor activity);  GO:0033275(actin-myosin filament sliding) | ko04530(Tight junction);  ko05416(Viral myocarditis) |
|  | comp59292_c1 | ABC transporter B family member 26 | 4 | GO:0031969(chloroplast membrane);  GO:0016021(integral to membrane);  GO:0005524(ATP binding);  GO:0042626(ATPase activity, coupled to transmembrane movement of substances) | ko02010(ABC transporters);  ko04142(Lysosome) |
| mul-miRn81 | comp28478_c0 | Glyceraldehyde-3-phosphate dehydrogenase | 0 | GO:0005737(cytoplasm);  GO:0004365(glyceraldehyde-3-phosphate dehydrogenase );  GO:0051287(NAD binding);  GO:0006096(glycolysis) | ko00010(Glycolysis / Gluconeogenesis);  ko05010(Alzheimer's disease) |
| mul-miRn86 | comp1624736_c0 | Cyclic dof factor 2 | 3 | GO:0005634(nucleus);  GO:0003677(DNA binding);  GO:0005515(protein binding);  GO:0008270(zinc ion binding);  GO:0006355(regulation of transcription, DNA-dependent);  GO:0006351(transcription, DNA-dependent) | |
|  | comp41360_c0 | Reticuline oxidase-like protein | 3.5 | GO:0031225(anchored to membrane);  GO:0048046(apoplast);  GO:0005829(cytosol);  GO:0005739(mitochondrion);  GO:0009505(plant-type cell wall);  GO:0005886(plasma membrane);  GO:0009506(plasmodesma);  GO:0005773(vacuole);  GO:0050660(flavin adenine dinucleotide binding);  GO:0008762(UDP-N-acetylmuramate dehydrogenase activity);  GO:0006979(response to oxidative stress) | |
|  | comp57017_c0 | 5'-adenylylsulfate reductase-like 5 | 4 | GO:0016021(integral to membrane);  GO:0045454(cell redox homeostasis) | |
|  | comp37818_c0 | Nascent polypeptide-associated complex subunit alpha-like protein 1 | 4 | GO:0022626(cytosolic ribosome);  GO:0009506(plasmodesma);  GO:0015031(protein transport);  GO:0009651(response to salt stress) | |
|  | comp54616_c0 | Quinone oxidoreductase-like protein | 4 | GO:0048046(apoplast);  GO:0009941(chloroplast envelope);  GO:0009570(chloroplast stroma);  GO:0010319(stromule);  GO:0009579(thylakoid);  GO:0035798(2-alkenal reductase );  GO:0035671(enone reductase activity);  GO:0008270(zinc ion binding);  GO:0009409(response to cold) | ko00010(Glycolysis / Gluconeogenesis);  ko00071(Fatty acid metabolism);  ko00350(Tyrosine metabolism);  ko00624(1- and 2-Methylnaphthalene degradation);  ko00641(3-Chloroacrylic acid degradation);  ko00830(Retinol metabolism);  ko00980(Metabolism of xenobiotics by cytochrome P450);  ko00982(Drug metabolism - cytochrome P450) |
|  | comp54806_c1 | Ethylene response sensor 1 | 4 | GO:0005789(endoplasmic reticulum membrane);  GO:0016021(integral to membrane);  GO:0005524(ATP binding);  GO:0051740(ethylene binding);  GO:0046872(metal ion binding);  GO:0005515(protein binding);  GO:0004872(receptor activity);  GO:0000155(two-component sensor activity);  GO:0009873(ethylene mediated signaling pathway);  GO:0010105(negative regulation of ethylene mediated signaling pathway);  GO:0018106(peptidyl-histidine phosphorylation) | |
