# Supplementary material for: MicroRNA Profiling During Mulberry (Morus atropurpurea Roxb) Fruit Development and Regulatory Pathway of miR477 for Anthocyanin Accumulation
Source: Front Plant Sci. 2021 Sep 8;12:687364. doi: 10.3389/fpls.2021.687364 (PMC8455890; doi:10.3389/fpls.2021.687364)
Supplement: Supplementary Table 3 — Forward primers used in miRNA qRT-PCR analysis. [file Table_3.DOC]

**Supplementary Table 3. Forward primers used in miRNA qRT-PCR analysis.** Uni-miR qPCR Primer supplied by the SYBR® PrimeScript® miRNA qPCR Starter Kit was used as reverse primer.

| **MiRNA-name** | **Primer sequence (5′-3′)** |
| --- | --- |
| mul-miR156a-5p | TTGACAGAAGAGAGTGAGCACTT |
| mul-miR157c-3p | GCTCTCTATGCTTCTGTCATCC |
| mul-miR319a | TTGGACTGAAGGGAGCTCCTC |
| mul-miR477 | ACTCTCCCTCAAGGGCTTC |
| mul-miRn37 | AATGAGGTTTGATCCGAAATC |
| mul-miRn58 | TTCGCCCCATTCATGATTAGA |
| mul-miRn81 | GACAAGAAGATCAAGATCGGAATC |
| Ath-U6 | ATGGCCCCTGCGTAAGGATG |
| Mul-U6 | ATGGCCCCTGCGTAAGGATGA |
