# Supplementary material for: MicroRNA Profiling During Mulberry (Morus atropurpurea Roxb) Fruit Development and Regulatory Pathway of miR477 for Anthocyanin Accumulation
Source: Front Plant Sci. 2021 Sep 8;12:687364. doi: 10.3389/fpls.2021.687364 (PMC8455890; doi:10.3389/fpls.2021.687364)
Supplement: Supplementary Table 4 — Primers used for mRNA qRT-PCR analysis. [file Table_4.DOC]

**Supplementary Table 4. Primers used in mRNA qRT-PCR analysis.**

| **Gene names** | **Sequence (5'-3')** | |
| --- | --- | --- |
|  | **Forward primers** | **Reverse primers** |
| Squamosa promoter-binding-like protein 16 | CCACAGACGGTTCCAAATAA | CAGAAGATAGAGAGCACGGC |
| Transcription factor TCP2 | CATTGTTCAAGGGACGAGA | GAGAGAGGAGAGAGGAGTGTGA |
| Putative disease resistance protein RGA1 | TGAGAGAGGGAAGGGTTGATA | ACCGAGCAAGTGTCGTCTT |
| Auxin response factor 1 | CTGGTCTGTTTCGCTATGG | AGGCATTTGCTGCTCTTG |
| Probable LRR receptor-like serine/threonine-protein kinase | GACCGAAGAATAGCATTTGG | CTGCCACACTAATCATCTCAAC |
| MulABCB19AS | ACCACCCCTGATGATCTC | CTCTCTACGCCTCTGAAG |
| Mul-ABCB19 | CATGATTTCTGGTAGTGTTG | CAGCCTCTAAATACTTCTTC |
| Ath-CHS | TACATGGCTCCTTCTCTGGA | GGTAGTGCAGAAGACGACAT |
| Mul-actin | CACTGAGGCTCCTTTGAACCC | AGGTCGAGACGGAGAATAGCATG |
| Ath-actin | GCACCCTGTTCTTCTTACCG | AACCCTCGTAGATTGGCACA |
